# ﻿Metabolic rate, sleep duration, and body temperature in evolution of mammals and birds: the influence of geological time of principal groups divergence

**DOI:** 10.3897/zookeys.1148.93458

**Published:** 2023-02-14

**Authors:** Valery M. Gavrilov, Tatiana B. Golubeva, Andrey V. Bushuev

**Affiliations:** 1 Department of Vertebrate Zoology, M.V. Lomonosov Moscow State University, 119991, Moscow, Russia M.V. Lomonosov Moscow State University Moscow Russia; 2 Zvenigorod Biological Station, M.V. Lomonosov Moscow State University, 119991, Moscow, Russia M.V. Lomonosov Moscow State University Moscow Russia

**Keywords:** Activity, basal metabolic rate, birds, body temperature, mammals, phylogeny, scaling, sleep duration, time of divergence

## Abstract

This study contains an analysis of basal metabolic rate (BMR) in 1817 endothermic species. The aim was to establish how metabolic scaling varies between the main groups of endotherms during evolution. The data for all the considered groups were combined and the common exponent in the allometric relationship between the BMR and body weight was established as *b* = 0.7248. Reduced to the common slope, the relative metabolic rate forms the following series: Neognathae – Passeriformes – 1.00, Neognathae – Non-Passeriformes – 0.75, Palaeognathae – 0.53, Eutheria – 0.57, Marsupialia – 0.44, and Monotremata – 0.26. The main finding is that the metabolic rate in the six main groups of mammals and birds consistently increases as the geological time of the group’s divergence approaches the present. In parallel, the average body temperature in the group rises, the duration of sleep decreases and the duration of activity increases. BMR in a taxon correlates with its evolutionary age: the later a clade diverged, the higher is its metabolic rate and the longer is its activity period; group exponents decrease as group divergence nears present times while with increase metabolic rate during activity, they not only do not decrease but can increase. Sleep duration in mammals was on average 40% longer than in birds while BMR, in contrast, was 40% higher in birds. The evolution of metabolic scaling, body temperature, sleep duration, and activity during the development of endothermic life forms is demonstrated, allowing for a better understanding of the underlying principles of endothermy formation.

## ﻿Introduction

Bennett and Ruben (1979) were the first to link the development of endothermy with the increased activity and endurance of endothermic animals. The study stated that increasing aerobic potentials causes increased activity, which is the main selective factor that formed the basis for the development of endothermy. Potentially, a resting animal is able to achieve a level of metabolism, which would enable the functioning of its basic systems and an instantaneous start of its activities, if necessary. This phenomenon is known as basal metabolic rate (BMR) (McNab 1997). Basal metabolic rate enables endothermy, but its origin is due to the necessity to support a high level of activity rather than for thermoregulation. Thermoregulation, like endothermy itself, is a by-product of increasing aerobic power in the process of the development of homoeothermic endothermic animals. Clarke and Pörtner (2010), Nespolo et al. (2017) and Ballesteros et al. (2018) developed this hypothesis, demonstrating the role of increased body temperature in enabling higher muscle efficiency, due to the better system of oxygen and nutrient transport, high nutrient assimilation rate and, consequently, increased growth rate. High BMR is considered to be the cause of high body temperature in endotherms, and represents an irreplaceable cost of self-maintenance (Kendeigh et al. 1977; Clarke and Pörtner 2010; Hayes 2010). In a recent article, using relevant data and modern phylogenetic methods, it was shown that both BMR and body temperature in mammals and birds independently change over geological time, depending on the ambient temperature (Avaria-Llautureo et al. 2019). Uyeda et al. (2021) reanalysed the data from the work of Avaria-Llautureo et al. (2019) using more biologically appropriate models, and found support for the directly opposite conclusions, corroborating previous evidence from the fields of physiology and palaeontology. In the present study we show the relationship between BMR and body temperature, and the geological time of taxon divergence on the principal groups of endotherms.

The aim was to verify the Bennett and Ruben hypothesis that the level of BMR determines the duration of activity of an animal. We wished to consider the increased duration of activity in evolution, and establish how this relates to BMR levels. Duration activity obviously varies between the groups of endotherms, how do we show the change in activity duration in different groups of animals in evolution? We suggest, in general terms, to determine the duration of activity in endothermic animals through the duration of sleep. Overall, the duration of sleep is inversely related to the overall activity, i.e., activity duration equals 24 hours minus sleep duration. Sleep (at least in endotherms) is a natural recurrent state in animals that decreases sensory activity, inhibits most voluntary muscle activity, and causes low levels of interaction with the environment (McNamara et al. 2009). In endothermic vertebrates, specifically birds and mammals (and only in these two groups), two distinct modes of sleep are alternated: rapid eye movement sleep (REM, or fast-wave [FWS] sleep) and non-REM sleep (or slow-wave sleep [SWS]) (Capellini et al. 2008). Overall, the duration of sleep and non-REM sleep is directly related to the intensity of metabolism, body mass and brain size (Aristakesyan 2016). It has been shown that, within vertebrates, the duration of sleep decreases from fishes and amphibians to birds and mammals (Karmanova 1982). In general, the duration of REM sleep in more deeply divergent (‘ancient’) taxa, both within mammals (e.g., platypus) and birds (e.g., ostrich), is much higher than in evolutionary younger groups (Lesku et al. 2011). Sleep is a very conserved behaviour within the whole evolution of animals (Joiner 2016); therefore, the duration of sleep is a good parameter that is the opposite of activity. We intend to find correlations between the average duration of sleep and the dimensionless BMR ratio for selected groups of endothermic animals. The development of the main existing groups of birds and mammals was not synchronous on a geological time scale. The first goal of this contribution was to assess changes in the scale parameters that associate BMR with body size in groups of mammals and birds which, in geological time, consistently diverged from the main stem of vertebrates. To begin with, we compared scaling indices for taxa of the infraclass rank in mammals and birds. In the present study, in accordance with the work of McNab (2008), we believe that the subclass Monotremata, infraclasses Metatheria and Eutheria of the subclass Theria will be such taxa. Phylogeny and classification of birds are still in the process of formation (Hackett et al. 2008; Gill et al. 2020; Kuhl et al. 2021). Most recently, new fossils and molecular evidence are providing a clear picture of the evolution of modern bird orders, but no strong consensus has emerged (Gill et al. 2020; Kuhl et al. 2021; Sangster et al. 2022). At the same time, Lasiewski and Dawson (1967) demonstrated that the basal metabolic rate of passerines (Aves: Passeriformes) is 40% higher than in other birds. Although this phenomenon remains a subject of debate, McNab (2009, 2016) convincingly demonstrated that passerine birds and non-passerine birds have different basal metabolic rates. We propose that neognath passerine and neognath non-passerine birds, and Palaeognathae, can be considered as subclasses of birds. These groups then provide a suitable model created by evolution that can be used to study the development of bird energetics.

We hypothesised that the above groups of endothermic animals which evolved in different geological times should exhibit varying BMRs, body temperature and sleep duration. The timescale is important because it enables the comparison of phylogenesis directly with the evolution of other organisms, and with the events of planetary histories, such as geological or climatic events. Revolutionary advances in molecular biology have permitted the estimation of times of taxon divergence on the basis of molecular clocks. After analysing these data, we found the most realistic the geological time divergence of these groups from the main trunk of vertebrates (see Benton 2005, 2020; Meng 2014; Brusatte et al. 2015; Beck and Baillie 2018; Phillips and Fruciano 2018; Benton et al. 2019).

Basal metabolic rate (BMR) is the minimal metabolic rate of endotherms. Comparative studies in animal energetics are based on the allometric equation between BMR and body mass (m):

BMR = *am^b^* or log BMR = log (*a*) + *b* * log (*m*)

where *b* is a scaling exponent (the slope of the regression line) and *a* is the allometric coefficient (antilog of the regression intercept). Analysis of studies on shifts in metabolic scaling across different evolutionary transitions in both exponential (Atanasov and Dimitrov 2002; DeLong et al. 2010; Uyeda et al. 2017; White et al. 2019) and allometric coefficients (Schmidt-Nielsen 1984; Zotin 1990, 2018a, b), led us to suggest that in the main groups of endothermic animals, the exponent will decrease depending on the geological time of divergence of the clades, while the value of *a* in the equation above will increase (Gavrilov et al. 2022a). The metabolic-level boundaries hypothesis (Glazier 2010) predicts that the scaling slope should vary mostly between 2/3 and 1, and that it should be related to metabolic rate (activity). The correlations of the geological time of divergence of taxa with the BMR level makes it possible to estimate the evolutionary norms for many phenotypic characteristics, including BMR. How body temperature and sleep duration are related to BMR level and the geological time of divergence of the group allows the establishment of more reliable correlations between historical and biological processes, and the development of a standardised framework for biological classifications which, in general, enables further progress in the field of comparative evolutionary biology.

Serious difficulties in discussing the functional meaning of the allometric coefficient *a* in different groups of animals are created by its strong dependence on the units of measurement and, especially, on the scaling exponent, which varies greatly between taxa, with respect to both different and the same taxonomic rank. In this study, we aimed to develop an effective way to compare BMR across groups, regardless of body size. We believe that the dimensionless ratio of BMR in different clades of endothermic animals will assist comparative studies. It can be obtained if the BMR dependencies on *m* are held to a common scaling index. We conducted a statistical test of the hypothesis that all six groups can be calculated with one common exponent. The inclusion of such a test is crucial, because only then is it possible to establish a dimensionless BMR ratio for the main selected groups of birds and mammals.

In this paper, we used a macroevolutionary perspective to look for the tipping points at which the principal clades of endotherms transitioned to new evolutionary allometric scaling relationships. We analysed in detail the parameters of metabolic scaling in six groups of endotherms: Monotremata, Marsupialia and Eutheria from mammals, and Palaeognathae, Neognathae-Non-Passeriformes and Neognathae-Passeriformes from birds. We then aimed to establish the dimensionless BMR ratio for the main groups of birds and mammals. We correlated this dimensionless relationship with the divergence time of the group and other parameters.

We compared the regressions obtained by the phylogenetic method of generalised least squares (PGLS) with the regressions obtained using ordinary least squares (OLS), to determine how phylogeny does affect the dimensionless BMR ratio for the main groups of birds and mammals.

## ﻿Materials and methods

### ﻿BMR dataset

We calculated BMR in the main groups of endotherms from the data published by Genoud et al. (2018) and Gavrilov et al. (2022a). As a result, we analysed the database comprising metabolic rates of 1817 endothermic species, which is available in the online Suppl. materials 1–3.

### ﻿Body temperature data

To correlate BMR levels and body temperature (***T*_B_**), we used Clarke’s data (table 10.2, Clarke 2017), Maloney’s data on Ratites (Maloney 2008) for body temperatures, and our own data for the BMR level.

### ﻿Sleep duration data

We expect that the duration of activity varies between the main groups of endotherms. Overall sleep duration is inversely related to the overall activity duration. We calculated sleep duration in the main groups of endotherms from the data in the work of Campbell and Tobler (1984), with the addition of some later sources for monotremes and palaeognaths (McNamara et al. 2009; Aristakesyan 2016).

### ﻿Date of time of divergence of taxa

In spite of some inconsistencies between the palaeontological record and molecular evidence, the sequence of emergence of the extant groups of endotherms may be presented as follows: monotremes 271 mya (Van Rheede at al. 2006; Zhou et al. 2013), marsupials 193 mya, eutherians 115 mya (Meng 2014; Phillips and Fruciano 2018), nearly simultaneous with the palaeognath birds 110 mya, followed by all non-passerine neognaths 90 mya, and finally passerine neognaths ca. 50 mya (Brusatte et al. 2015; Beck and Baillie 2018).

### ﻿Statistical analysis

The body mass and BMR data were log_10_-transformed before analysis to account for allometric scale. All scaling exponents in allometric equations that are used in our study, were based on ordinary least squares (**OLS**), weighted least squares (**WLS**) or phylogenetic generalised least squares (**PGLS**) regressions of log_10_ (**BMR**) ~ log_10_ (m). Because our sample of different endothermic groups varied greatly in the number of species, in some cases we augmented the OLS analysis with WLS by incorporating the sample size information into the regression. Both OLS and WLS regressions were conducted using the basic R function ‘lm’ (R Core Team 2019). The p value for the regressions of the OLS is shown in the figures, the p values for the WLS regressions in all cases were p < 0.05 and they are not shown. To test for difference in allometric coefficients of regressions in different principal groups of endotherms, we used an ANCOVA with log (BMR) as the dependent variable, and log (*m*) as the covariate. To test for differences in the slopes of the two regression lines, we tested the model with the interaction term of log (*m*) and the grouping factor versus the model without interaction using the ‘Anova’ function. The differences between observed slopes and the theoretical slope of 3/4 were tested using Welch’s *t*-test. To estimate standard errors of allometric coefficient *a*, we used the ‘delta method’ function from the ‘car’ package in R (Fox and Weisberg 2019). The significance level in all analyses was set as p = 0.05.

### ﻿Level of BMR and dimensionless ratio of BMR

We applied a test of the homogeneity of the slopes, which in our case tests the null hypothesis H_0_: b1 = b2 = b3 = b4 = b5 = b6. Using the R lm procedure (Fox and Weisberg 2019) and the Akaike and Bayesian information criteria (**AIC** and **BIC**), we compared three OLS models for analysing the data: simple regression without group factor (taxon), regression with separate intercepts, and regression with separate slopes and intercepts. We found that our data were best described by the model with separate intercepts and a fixed slope. We suggest that the model with one common slope (0.7248) and a separate intercept for each of the six groups of endotherms is best, in the sense that it is the simplest model which explains the data. Further, of the three models, the model with separate intercepts and fixed slope has the lowest BIC -1778.22. Further information can be found in Gavrilov et al. (2022b).

### ﻿Phylogenetic analysis

The avian phylogeny was extracted from the birdtree.org database (http://www.birdtree.org) using the study by Hackett et al. (2008) as the basis for phylogenetic reconstruction. The avian tree construction method is detailed in Bushuev et al. (2018). The phylogenetic tree and data on BMR of mammals were obtained from the study by Genoud et al. (2018). We used the phylogenetic generalised least squares model (**PGLS**) to take the phylogenetic signal into account in allometric analyses (Freckleton et al. 2002; Grafen 1989), using the ‘pgls’ function from the ‘caper’ package v. 1.0.1 (Orme et al. 2018). Phylogenetic signal in mass-independent BMR was estimated with Pagel’s lambda (λ) (Pagel 1999) via a maximum likelihood (**ML**) approach, using the same function. To test the differences in intercepts and slopes of phylogenetic regressions in different groups of endothermic animals, we determined the significance of the group term and its interaction using the same function.

## ﻿Results

### ﻿Allometry of metabolic rate in major groups of mammals and birds

**Figure 1.**
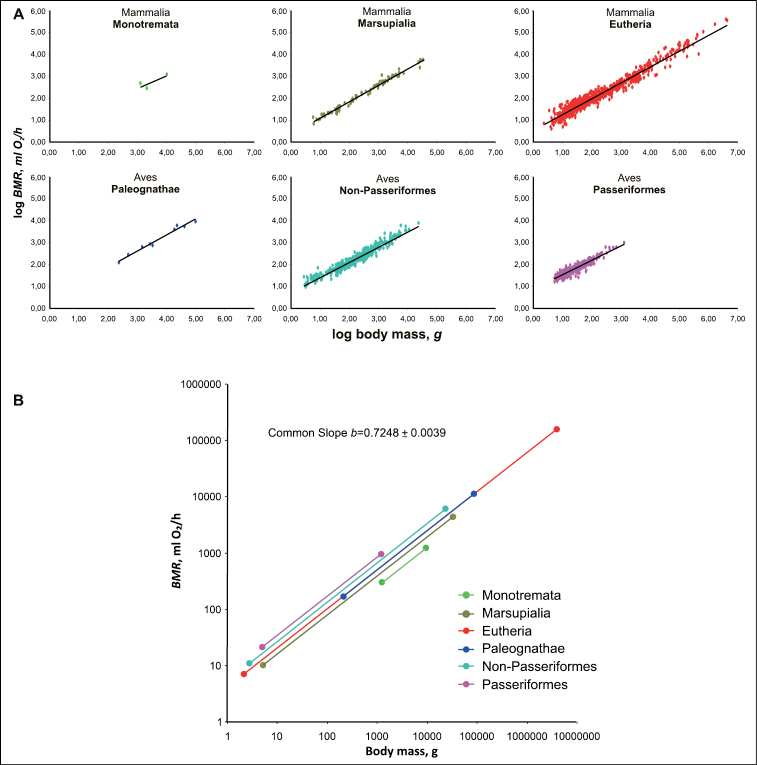
BMR as function of body mass in principal groups of endotherms **A** original regressions **B** regression normalised at common slope 0.7248 and the lines are drawn in accordance with the size range of the group.

We calculated the allometric equations for BMR of all major groups of mammals and birds. The regression lines in these groups differ slightly in slope, but differ significantly in intercepts (Fig. 1, Tables 1, 2).

**Table 1.** Parameters of allometric equation for basal metabolic rate in principal groups of endothermic animals obtained from OLS analyses.

Group	Number of species	Body mass range, g	OLS: a ± SE	OLS: b ± SE	OLS: R^2^
Mammalia	817	2.2–4037500	3.248±0.107	0.735±0.006	0.956
Monotremata	3	1284–10300	5.861± 0.512	0.565±0.387	0.681
Marsupialia	84	5.4–32490	2.300±0.152	0.753±0.011	0.983
Eutheria	730	2.2–4037500	3.326±0.115	0.736±0.006	0.956
Aves	1000	2.8–92400	7.435±0.167	0.648±0.005	0.940
Palaeognathae	9	220.8–92400	3.221±1.147	0.727±0.041	0.978
Non-Passeriformes	404	3.2–23370	5.507±0.262	0.691±0.009	0.939
Passeriformes	587	5.1–1203	7.379±0.256	0.668±0.010	0.871

**Table 2.** Parameters of allometric equation for basal metabolic rate in principal groups of endothermic animals obtained from PGLS analysis.

Group	Pagel’s λ	PGLS: a ± SE	PGLS: b ± SE	PGLS: R^2^
Mammalia	0.870	2.357±0.632	0.735±0.009	0.888
Monotremata	0.000	5.861±NA	0.565±0.387	0.681
Marsupialia	0.214	2.407±0.222	0.746±0.013	0.976
Eutheria	0.813	2.910±0.393	0.733±0.011	0.874
Aves	0.664	5.514±0.605	0.679±0.010	0.830
Palaeognathae	0.000	3.221±0.871	0.727±0.041	0.978
Non-Passeriformes	0.630	4.833±0.589	0.708±0.014	0.865
Passeriformes	0.443	7.818±0.610	0.642±0.014	0.780

**Notes.** Allometric equation: BMR = *a*m*^b^*, where BMR is basal metabolic rate in ml O_2_/hour, m – body mass in g, *a* – allometric coefficient, *b* – scaling exponent, obtained from OLS and PGLS analyses.

### ﻿The effect of phylogeny

It is noteworthy that we are not aiming at revisiting numerous previous research works concerning the effect of phylogeny on metabolic scaling. We are interested in how the results obtained by regressions PGLS and OLS are reflected in the dimensionless ratio BMR, in groups and on scaling options. Here, we present comparisons of slopes and intercepts from both OLS and PGLS regressions of log (BMR) ~ log (*m*), obtained for several major groups of endotherms.

Using OLS, coefficient *a* was significantly different for two of the vertebrate classes (p < 0.001); the slopes of the regression lines also differed (p < 0.001). Using PGLS, for birds and mammals, both *a* and *b* were also significantly different (p < 0.001).

For all major groups, the calculation using PGLS had more influence on *a*, the allometric coefficient, than on the slopes of the regression lines. Paleognathae and Neognathae did not differ in the intercepts of PGLS regressions, but rather they differed at the tendency level (i.e., tending to differ): neognaths tend to have higher BMR. The same can be said about the differences between Passeriformes and non-Passeriformes: the former tend to have higher BMR (more precisely, they tend to differ) and the BMR of Passeriformes is higher.

Pagel’s lambda is a potential measure of “phylogenetic signal”, the extent to which correlations in common traits reflect their shared evolutionary history. The effect of phylogeny was greater for both *a* and *b*, if the value of Pagel’s λ for the group was greater. The effect of lambda on scaling options is as follows:

*a*OLS-*a*PGLS = – 0.591+ 1.8993 λ, R^2^ = 0.3113;

*b*OLS-*b*PGLS = 0.0504-0.0497 λ, R^2^ = 0.1397.

With an increase in lambda, the difference in the allometric coefficient *a* between the OLS and PGLS measurements increases, while the difference in exponent *b* decreases. The higher the value of Pagel’s λ for the group, the more its effect of phylogeny was greater for this group (Table 2).

### ﻿Average level of BMR and dimensionless ratio of BMR

We determined that of the three models, according to the BIC criterion, the best model is the one with a common slope and separate intercepts. To obtain the ability to compare the BMR in different groups, we recalculated the equations transformed with a common average *b* = 0.7248 using the standard OLS procedure.

**Figure 2.**
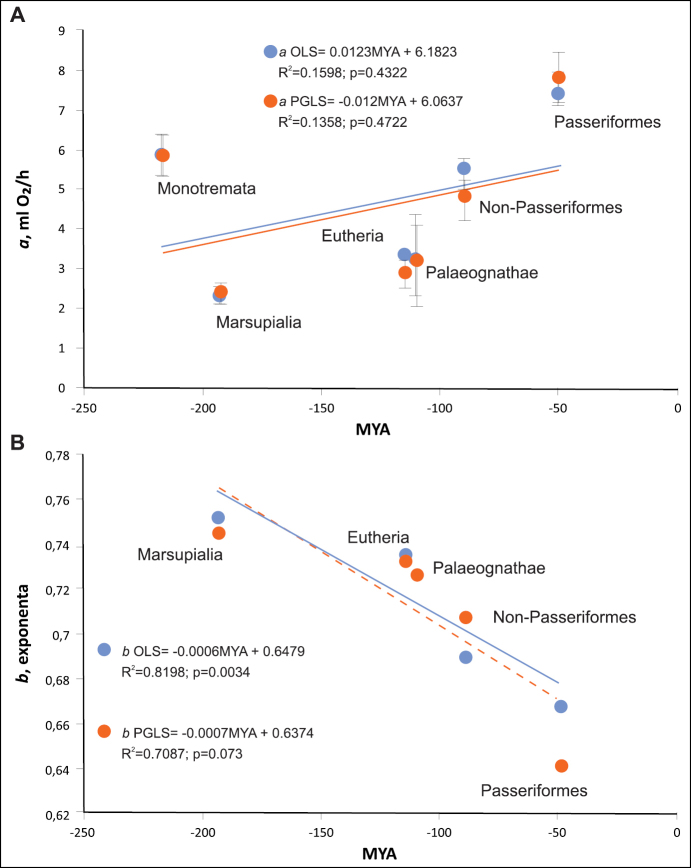
**A** the allometric coefficient in six groups of endothermic animals, depending on the geological time of divergence of the clades: by the phylogenetic generalised least squares (PGLS) and ordinary least squares (OLS). *a* (mlO_2_/h) is the allometric coefficient (antilog of the regression intercept) **B** the exponents in six groups of endothermic animals, depending on the geological time of divergence of the clades: by the phylogenetic generalised least squares (PGLS) and ordinary least squares (OLS). *b* is a scaling exponent (the slope of the regression line).

Reduced to the common slope *b* = 0.7248, the value of *a* gradually and understandably increases from Monotremata to Passeriformes, and it is between 1.63 for Monotremata and 6.18 for Passeriformes (Table 3, Fig. 2), reflecting the increase in the level of energetic organisation. The relative metabolic level forms the following series: Passeriformes – 1.00, Non-Passeriformes – 0.75, Palaeognathae – 0.53, Eutheria – 0.57, Marsupialia – 0.44, and Monotremata – 0.26.

**Table 3.** Changes in allometric coefficient *a* when reduced to common slope *b* = 0.7248 ± 0.0039.

Group	*a* at original scaling exponent (slope)	R^2^	*a* at common slope *b* = 0.7248	R^2^	*a*/*a*_Passeriformes_, BMR ratio
Monotremata	5.861	0.681	1.63	0.665	0.264
Marsupialia	2.3	0.983	2.69	0.980	0.435
Eutheria	3.326	0.956	3.53	0.956	0.571
Paleognathae	3.221	0.940	3.29	0.978	0.532
Non-Passeriformes	5.507	0.9395	4.65	0.939	0.752
Passeriformes	7.379	0.79070	6.18	0.871	1.000

**Notes.** New equation options for allometric equation: BMR = *am^b^*, where BMR is basal metabolic rate in ml O_2_ / hour, *m* – body mass in g, *a* – allometric coefficient, obtained after recalculation with scaling exponent *b* = 0.7248; *a* / *a*Passeriformes ratio *a* relative Passeriformes, i.e., dimensionless ratio of BMR of selected groups.

### BMR and divergence time of various groups of endotherms

The analyses of metabolic scaling in the six groups illustrate that the variation in metabolic scaling relationships is systematically related to metabolic level. Metabolic scaling in the main groups of endothermic animals correlates with their evolutionary age: the younger the group is, the higher is its metabolic rate, but it increases more slowly with increasing body weight (Fig. 2).

**Figure 3.**
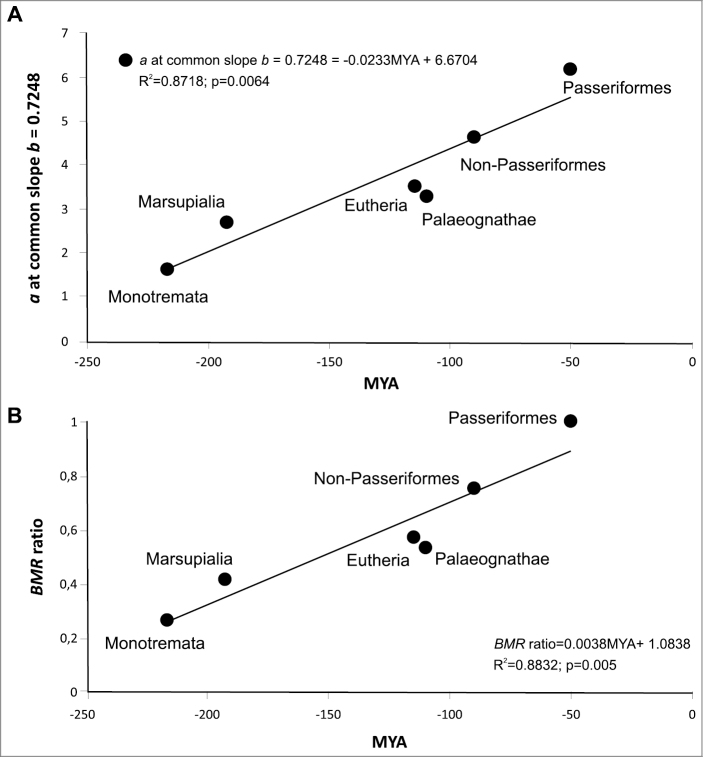
**A** the allometric coefficient *a* (mlO_2_/h) in different groups, is formulated by recalculating the equations and transforming with a common average *b* = 0.7248, depending on the geological time of divergence of the clades **B**BMR ratio in the six principal groups of endothermic animals are expressed in relation to BMR of Passeriformes (a non-dimensional coefficient of the intercept), dependent on the time of appearance in evolution.

BMR increases in evolutionarily younger groups (see also Table 3). Equations that relate BMR (relative to Passeriformes) to the divergence time of the group use the following formula: BMR ratio = 0.0038MYA + 1.0838 (Fig. 3)

Applying regressions with a common slope b = 0.7248 sharply increase both R^2^ and the reliability of the regressions, to a higher degree when using the dimensionless BMR ratio than in units of ml O_2_/h per g (Fig. 3). The metabolic rate increases as the time of group divergence approaches present, represented as BMR ratio and in units of ml O_2_/h per g. BMR ratio regressions have significantly higher R^2^ and higher confidence levels.

### ﻿Body temperature, dimensionless ratio of BMR, and average level of BMR

**Figure 4.**
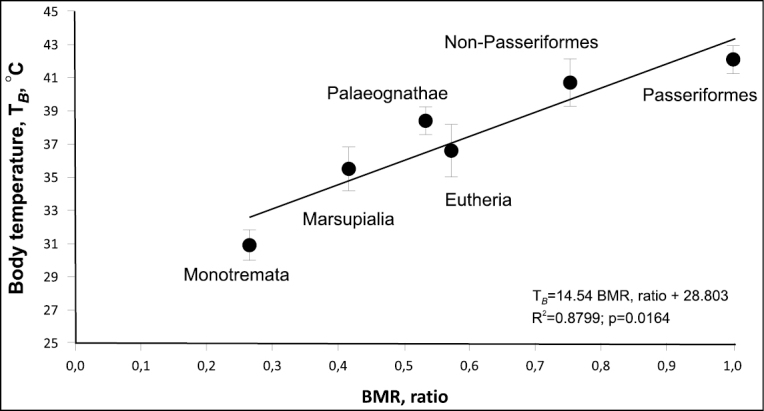
Average body temperature in different groups of mammals and birds as a function of BMR ratio.

High BMR is correlated with high body temperature in groups of endotherms (Fig. 4)

### ﻿Sleep duration, activity duration, and the geological time of appearance of the group in evolution

**Figure 5.**
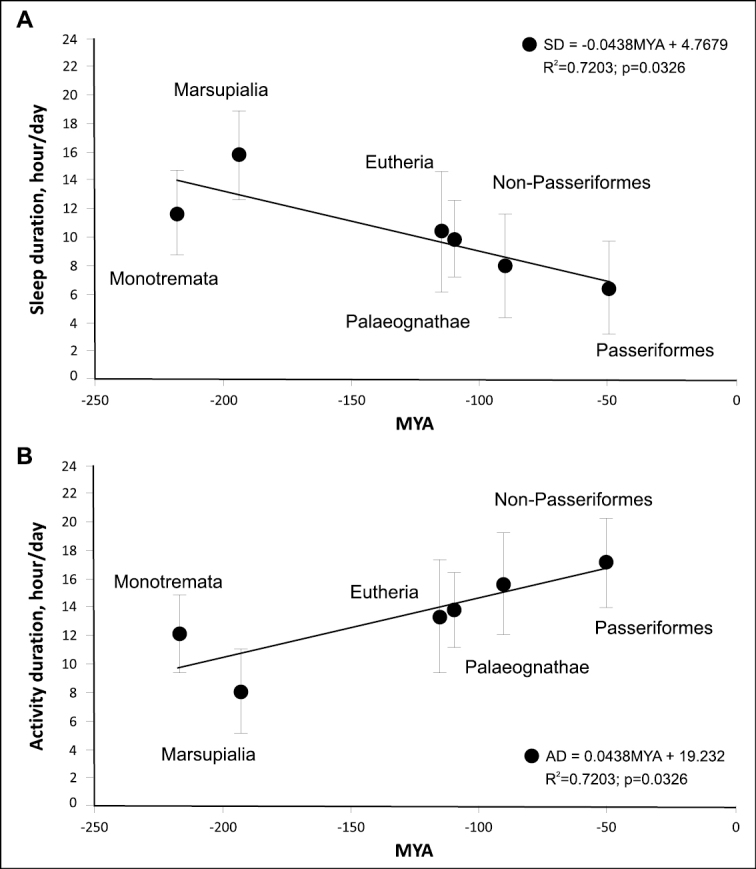
**A** sleep duration in the six main groups of endothermic animals depending on the geological time of appearance of the group in evolution. Regression lines and statistics in the figure were calculated using the OLS method. Using the weighted least squares (WLS) method, p = 0.01548 **B** activity duration (24 minus sleep duration) as a function of geological time since taxon’s divergence, in mya, in different groups of endotherms. Regression lines and statistics in the figure were calculated using the OLS method. Using the weighted least squares (WLS) method, p = 0.01548. The total duration of sleep is an indicator of the reverse value of total activities.

We performed a meta-analysis of sleep duration in the main taxa of endotherms (Fig. 5A) and calculated the activity duration (24 h minus sleep duration, Fig. 5B).

### ﻿Activity duration and the BMR level

**Figure 6.**
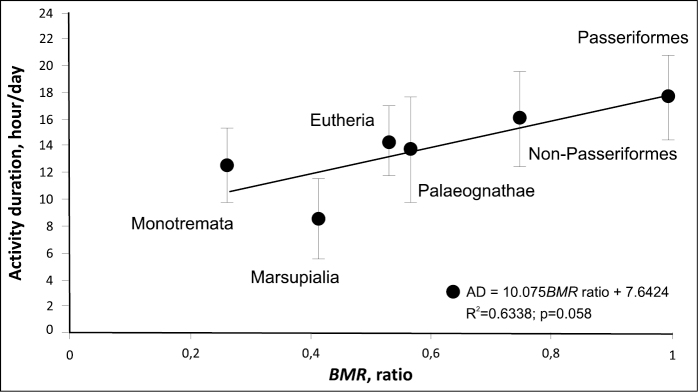
Activity duration as a function of the relative level of BMR.

The size-corrected BMR in the six groups of endothermic animals is correlated with the duration of their activity, with this parameter increasing from monotremes to Passeriformes (Fig. 6).

The greater the proportion of the day when animals are active, the higher their BMR is. Placental mammals and palaeognath birds exhibit similar BMR; i.e., terrestrial animals that do not fly have the same BMR level and the same duration of activity.

## ﻿Discussion

In this study, we established correlations of various biological traits, with the development of the metabolic level adjusted for body-size effects, by using an analysis of covariance. We determined the effect by comparing the elevations (indicated by Y-intercepts) of multiple size-scaling relationships, which are preliminarily brought to a common slope. This statistical method thus allows comparison of metabolic rate using the allometric coefficient “*a*” among different groups, which is reasonable since exponent “*b*” is fixed. We also found the dimensionless ratio of *BMP* levels in these groups. We found correlations of these levels with the average body temperature at group level; the relationship between the level *BMP* group and sleep duration; the relationship between levels of *BMP* in different taxa with the time of their divergence from the main clade of vertebrates; and the relationship between the metabolic levels and duration of sleep-in different groups. Now, we intend to look at how the level of BMR relates to the state of these groups at the present time. We correlate BMR levels and body temperature with known events in the history of the Earth.

### BMR and the diversity of species

Endothermic taxa with higher BMR contain more species. Palaeognaths include 57 extant species. Passeriformes are the largest order of birds and include ca. 6000 species or 60% of the 10,000 extant avian species. The body sizes of passerines vary from the Raven (*Corvuscorax*), whose mass can reach 1.5 kg, to the tiny Short-tailed pygmy tyrant (*Myiornisecaudatus*) (4.2 g). However, most birds from this order have body mass between 10 and 80 g, and a mean passerine is smaller than a mean bird of any other order. Neognath non-passerines include ca. 4000 species of 25 orders (Del Hoyo and Collar 2014, 2016). The reasons behind the specific diversity of passerines in space and time have been recently discussed in several studies (Wiens 2011; Gavrilov 2014; Jønsson et al. 2015; Pigot et al. 2016).

Most extant mammals (99%) belong to the subclass Theria, which consists of 5136 eutherian species and 346 marsupial species. Just five species of monotremes survive today (Jones and Safi 2011; Flannery et al. 2022). If all extant vertebrates are considered (data from five vertebrate groups by John P. Rafferty of Encyclopaedia Britannica: fishes, amphibians, reptiles, birds, mammals), the amphibian size ranges from *Paedophryneamanuensis*, the smallest frog in the world weighing 0.15 g to *Mastodonsaurusgiganteus*, the Chinese giant salamander, which weighs up to 70 kg. Of the living reptiles, the smallest is the dwarf gecko (*Sphaerodactylusariasae*), 16 mm in length and with a maximum weight of 0.14 g whereas the largest is the combed crocodile which is up to 7 m in length and weighing up to two tons. In mammals, the smallest is the Pygmy manytooth (*Suncusetruscus*): its average weight is ~ 1.8 grams (1.3–2.4 grams) and the largest is the Blue whale (*Balaenopteramusculus*) which reaches 33 m in length, and has a mass that can exceed 150 tons. The smallest bird is the Bee hummingbird (*Mellisugahelenae*, endemic to Cuba): its body length is 5.51 cm and its weight is 1.95 g. The largest bird, a flightless paleognath, is the African ostrich (*Struthiocamelus*), which weighs 150 kg. The largest flying Neognathae bird is the Andean condor (*Vúltur gryphus*) which may weigh up to 15 kg. The largest flightless Neognathae bird is the emperor penguin (*Aptenodytesforsteri*) which weighs 40 kg. The smallest fish, and at the same time the smallest of all vertebrates, is the Pandaka goby (*Pandakapygmaea*), which is found in lakes in the Philippine Islands. The length of the body of this species is only 1 cm and its weight is 4–5 mg. The Whale shark (*Rhincodontypus*) is the largest fish. The length of the body is fifteen meters, and its weight can reach up to twelve tons.

We combined data on ectotherms and endotherms using our data from previous works (Gavrilov et al. 2022a, b) on reptiles and amphibians on scaling of metabolic rates and literature data (White and Seymour 2003; White et al. 2006) also as on the number of species in the taxon and its size range (Table 4).

**Table 4.** How taxon divergence geological time and metabolic rate affect the metabolic scaling in nine groups of vertebrates, the number of extant species in the taxon and their size ranges.

Taxa	Taxon divergence geological time, *M*YA	*a* at common slope, mlO_2_/h for m = 1 g	*b*	Number of extant species	Body mass range of extant species, g
Fishes	465	0.264	0.88	25000	0.004–12000000
Amphibia	365	0.366	0.88	4000	0.15–70000
Reptilia	322	0.398	0.76	8000	0.14–2000000
Monotremata	217	1.63		4	2000–10000
Marsupialia	193	2.69	0.75	346	20–100000
Eutheria	115	3.53	0.74	5136	1.8–150000000
Palaeognathae	110	3.29	0.73	57	30–156000
Non-Passeriformes	90	4.65	0.69	4000	1.95–40000
Passeriformes	50	6.18	0.67	6000	4.2–1500

**Notes.** Allometric equation: BMR or SMR = *a*m*^b^*, where BMR is basal metabolic rate for endotherms, SMR is standard metabolic rate for ectotherms in ml O_2_/hour, m – body mass in g, *a* – allometric coefficient, *b* – scaling exponent, obtained from OLS analyses. Coefficient *a* for Fishes, Amphibia and Reptilia obtained with common slope *b* = 0.840 and for all endotherms obtained with common slope *b* = 0.7248.

May we emphasise that the patterns of metabolic scaling found by us for endotherms (Gavrilov et al. 2022b and present study) regarding the change in indicators *a* and *b* depending on the geological time of the origin of the group are fully confirmed by the data on all vertebrates. The allometric coefficient *a* increases as the origin of the group approaches. to date *a* = 5.4465-0.0135mya, R^2^ = 0.8477, p = 0.0004, *n* = 9 groups, exponent *b* decreases on the contrary *b* = 0.6581+0.0005 mya, R^2^ = 0.8677, p = 0.0008, *n* = 8 groups (we exclude from further discussion the results of the allometric analysis of Monotremes regarding the slope of the regression line, as this group is represented in the database by only three species): *b* = 0.8487 – 0.0322*a*, R^2^ = 0.7838, p = 0.0034. We previously found a negative linear relationship for scaling exponents and the allometric coefficient in 5 groups of endothermic vertebrates in units of ml O_2_/h per g: *b* = 0.79 – 0.0185*a*, R^2^ = 0.987 (Gavrilov et al. 2022b). This correlation is confirmed for all 8 groups of vertebrates: *b* = 0.8487 – 0.0322*a*, R^2^ = 0.7838, p = 0.0034.

As for the number of species in the taxon and their size range, there is no distinct correlation with the time of divergence. The oldest group, fish, have the highest species diversity, the number of species in this paraphyletic group of vertebrate aquatic jawed animals, which differ in gill breathing throughout the postembryonic development of the organism, is comparable to the number of species in other groups of vertebrates together. They are distinguished by the largest size range and the smallest sizes among vertebrates (Table 4). The largest modern vertebrates are Eutheria. The sizes of the largest Eutheria are quite comparable with the largest animals in the history of the Earth. The minimum size of ectotherms is an order of magnitude smaller than that of endotherms.

From our point of view, the acquisition of endothermy increases the minimum size of animals (apparently only in animals of ~ 2 g and above endothermy can be established), while the maximum sizes are controlled by other factors (for example, flying birds obey the rules of aerodynamics). Variation in the scaling exponent (slope) was caused primarily by the fact that mammal and bird datasets included species from the six major groups in various proportions. Different slopes appear due to different BMR values across taxonomic groups, sample sizes, range of body mass across taxa, and different representations of species within each group. For example, the overall slope for all birds, *b* = 0.674, is obtained due to the highest BMR of passerines, which are concentrated in the lower part of the size range, and the lowest BMR of palaeognaths, which form the upper part of the size range. In addition, the higher the exponent of body weight (*m*) in the BMR equation, the larger the range of sizes group will be. In this case, the lack of forces for movement (which grows ∝ *m*^0.83^) will not be a size limiter. The size range in mammals with an empirical exponent for body weight in the equation for BMR*b* = 0.735 is two orders of magnitude higher than in birds with an exponent of 0.668. If we consider our results in the broader context of the metabolic optimum of life (Makarieva et al. 2008), then only when comparing mammals and birds is a significant difference in size revealed. The size range of birds is two orders of magnitude smaller, and hence the mass-specific increase in the metabolic rate of birds associated with a decrease in size occurs. This is especially pronounced in Passeriformes, which is of evolutionary importance (Wiens 2011; Gavrilov 2014; Jønsson et al. 2015; Pigot et al. 2016).

### ﻿Body temperature, BMR, and maintaining thermal balance

To maintain a constant body temperature, endothermic birds and mammals must maintain a balance between heat production and dissipation. Heat dissipation is the main thermodynamic problem that animals need to solve. To lose the heat produced during activity, the animals need efficient mechanisms based on a well-developed blood circulation system, and the ability to regulate thermal isolation of the skin. Body temperature of extant birds and mammals is similar, which is explained by the thermal conditions that are typical on Earth, protein properties and thermal optimum of biochemical reactions. We suggest that it was not until the mid-Cretaceous that birds and mammals, with a modern level of BMR, formed. Apparently, only a particular level of BMR makes it possible to maintain body temperature that is necessary for homeothermic endothermy. It should be emphasised that King (1974) demonstrated as early as the 1970s, that permanent endothermy on the Earth is not possible with body temperature below 37 °C. We also showed that existence of animals under ambient temperatures, similar to their body temperature, is only possible due to evaporative heat loss (Gavrilov 2014). Obviously, body temperature should be higher than ambient temperature that is observed for any protracted period of time. Otherwise, if body temperature is lower than ambient temperature for considerable periods, no productive work will be possible, because all available energy will be spent for evaporation, which is necessary to avoid overheating. Both currently and in the Mesozoic, when endothermic animals evolved, the limit of this temperature has been ca. 35 °C. Therefore, to be able to dissipate heat through the skin, endothermic animals had to have body temperature above 35 °C, and the higher the temperature, the more effective the process would be. The upper limit of possible body temperature is governed by protein biochemical properties, and not just by thermodynamics. Therefore, body temperature of extant mammals is 37 °C (with the notable exception of monotremes, whose body temperature is 32 °C, which may hint at their emergence under the conditions of ambient temperature not exceeding 30 °C), and of birds ~ 40 °C. Permanent homeothermy with body temperature of 15, 20 or 30 °C is not possible if ambient temperature, even seasonally, was sustained above these values. Unlike body temperature, increased aerobic metabolism could develop gradually and be supported by the selection, because increased activity is beneficial at any stage. It is possible that, with or without taking into account this factor, the authors came to different conclusions regarding how both BMR and body temperature in mammals and birds, independently or synchronously, changes over geological time, depending on the ambient temperature (Avaria-Llautureo et al. 2019; Uyeda et al. 2021).

### ﻿Cretaceous thermal maximum and development of endothermy

We suggest that Triassic and Jurassic mammals and birds, whose fossil remains are fragmentarily known, did not possess fully developed endothermy, and in this regard were similar to monotremes or those even less advanced. The Mesozoic was the warmest and most stable period in the history of the Earth (Zalasiewicz and Williams 2015). If the first mammals and birds attempted to establish homeothermy with body temperature below 37 °C, they had difficulty maintaining thermal balance in the warm climate of the Mesozoic. This is the most probable reason why the first mammals were nocturnal (Benton 2005, 2020; Clarke and Pörtner 2010; Nespolo et al. 2017). Only when the evolutionary development of blood circulation and respiratory systems allowed them to maintain their body temperature at 37 °C, could they switch to a diurnal lifestyle. The aforementioned systems and isolating skin allowed them to control heat dissipation without increasing evaporation and to develop homeothermy with the obligatory basal metabolic rate, similar to the BMR of extant marsupials and palaeognaths. Ancient mammals with imperfect homeothermy likely experienced the most serious thermoregulatory problems during a thermal maximum in the Cretaceous (~ 85–90 mya), which was the warmest period on the Earth in the most recent 200 million years (Norris 2018). Ambient temperature in the tropical areas reached 35–40 °C which made it difficult to maintain thermal balance for the animals with imperfect homeothermy. It might have been the reason for the extinction of Trituberculata, a subclass of ancient mammals that lived 215–85 mya. This group is believed to be sister to the placentals and marsupials, i.e., to all extant mammals with the exception of monotremes.

We believe that during the entire Mesozoic era, various clades of mammals and birds evolved, in which homeothermic endothermy developed. In these clades, morpho-physiological traits originated that led to the development of full endothermy. It should be emphasised that all extant orders of birds and mammals evolved after the Cretaceous thermal maximum (Phillips and Fruciano 2018; Benton 2020). The idea that mammals evolved true endothermy through heterothermy (Lovegrove 2017) is probably correct because it explains how the metabolic rate can gradually increase. In this sense, we support the perspective of Lovegrove (2017), and agree that early mammals were not homeothermic, and instead had low body temperature, which means heterothermy.

### BMR and activity level

The BMR level in the group correlates with the duration of activity. The BMR level and duration of the group’s activity increase as the geological time of the group’s appearance approaches the present time. Since Brody (1945), it is considered that the energy expenditure for standard work in different groups of animals is a multiple of BMR (referred to as Brody’s principle). Let us consider how the BMR level is related to the known levels of energy expenditure in different groups of endotherms. The mean daily energy expenditures for life supporting activities, measured under natural conditions at optimal ambient temperatures in extant animals by the doubly labelled water technique, the so-called field metabolic rate ( FMR), suggest the following allometric dependences on the body mass (Nagy 2005): for mammals, FMR = 10.04*m*^0.734^ (N = 79, r^2^ = 0.950), and for birds, FMR = 21.85*m*^0.681^ (N = 95, r^2^ = 0.938), where FMR is in ml O_2_ hour^–1^ and *m* are body mass in g. These equations demonstrate that the amount of energy spent by birds for their life supporting activities is greater by 2-fold of magnitude than mammals and, correspondingly, they consume more energy. At the same time, the differences in the BMR level in birds and mammals do not exceed 40%. Increased energy expenditure in birds for life supporting activities is also due to the longer duration of bird activity.

Maximum energy consumption has also been studied in birds and mammals. In mammals, maximal oxygen consumption during exercise MMR (ml O_2_ hour^–1^) = 17.187m^0.8598^ R^2^ = 0.691 (Dlugosz et al. 2013; Albuquerque et al. 2015), in birds, MMR of flying birds, or maximum aerobic metabolic rates during treadmill exercise MMR (ml O_2_ hour^–1^) = 83.84m^0.6974^ R^2^ = 0.7266 (White et al. 2019). Consequently, the maximum oxygen consumption in mammals increases, on average, 5.5× compared to BMR; moreover, it is higher in large species, and in birds on average, 11.5×compared to BMR. The ratio of maximum oxygen consumption in birds and mammals is 1: 0.48, which is lower than the ratio of BMR.

Other well-documented levels of energy expenditure have been reported in birds. This is averages daily energy expenditure of caged birds for long-term sustained rate of biological activity and the expenditure of energy in flight. In this case, we can compare the ratio of energy expenditure for these activities in passerine and non-passerine birds. Further, the existing energy averages daily energy expenditure of caged birds for long-term sustained rate of biological activity and the expenditure (Kendeigh et al. 1977; Peters 1983; Calder 1984) and the expenditure of energy for flight (Berger and Hart 1974; Tucker 1974; Kendeigh et al. 1977; Ward et al. 2004; Schmidt-Wellenburg et al. 2008) is correlated in these groups as 1: 0.73–0.75, which corresponds well to the difference in the BMR in passerine and non-passerine birds.

### ﻿Advent of angiosperms and development of endothermy

We suggest that the advent of angiosperms triggered the rapid development of endothermic animals, i.e., animals are able to maintain at a certain metabolic rate for sustained homeothermy. This process, endothermisation of vertebrates, took place in the branches leading to the mammalian and avian clades (Gavrilov, 2013). Angiosperms evolved in the early Cretaceous (Krassilov 1997; Doyle and Endress 2014). The advent of these plants, which were initially entomophilic (i.e., they attracted insects for pollination), caused profound evolutionary changes among insects and helped form the current insect fauna, resulting in ‘angiospermisation of the world’ (Ponomarenko 1998). Flowering plants changed ecosystems. The biomass of both angiosperms and the invertebrates that utilised them increased. Ecosystems became patchier. The natural succession of gymnosperm ecosystems was disrupted, causing significant changes in local faunas (Krassilov 1997; Feilda et al. 2011; Rasnitsyn et al. 2016; Benton 2020). Angiosperms utilise many macro- and microelements more actively than gymnosperms do, resulting in more advanced mineral circulation. The ability of flowering plants to bind calcium deposited in the soil after the death of these plants and to deacidify soils was especially important. This process enabled the formation of highly fertile soils that accumulated large stores of biogeochemical energy (Ponge 2003; Feilda et al. 2011). Mesozoic vegetation was a poor food source for large terrestrial vertebrates (Ponomarenko 1998) and did not allow the survival of large plant-eaters characterised by high energy turnover. In the Cretaceous, community production first declined and subsequently increased significantly when angiosperms expanded over new areas, due to both the plant and invertebrate components of the communities (Rasnitsyn et al. 2016). Herbaceous flowering plants produced abundant protein-rich and quite sustainable biomass. This enabled the development of a considerable biomass of plant-eating animals, which in turn allowed large increases in the numbers of carnivores. When flowering plants became abundant in the mid-Cretaceous, leaf consumers boomed nearly immediately, among which leaf miners increased first. However, mass consumption of the green parts of plants did not start until grass biomes became abundant. Their advent enabled the development of ecosystems based on large biomasses of diverse plant-eating animals, their predators and parasites, coprophages and necrophages (Ponomarenko 1998; Rasnitsyn et al. 2016).

It was not until the mid-to-late Cretaceous that birds and mammals started to play the leading roles in the communities that they have at present. Birds lost their teeth in the late Cretaceous, and the existing mammals already included both marsupials and placentals. These developments were related to the general evolution of the organic world, which caused the advent of flowering plants in the Mesozoic, along with the related fauna of invertebrates and a high diversity of flying insects. Only the development of angiosperms and the related diversity of invertebrates provided a sufficient food base to supply for the development of endothermic animals. Angiosperms and insects provided food resources that were necessary for endotherms but were not suitable for many reptiles adapted to the utilisation of Mesozoic flora and fauna, which enabled the ecological expansion of endotherms. Birds and mammals replaced reptiles in many mainstream ecological niches, adapted to various habitats and quickly expanded to a broad range of size classes (mammals – eight orders of magnitude by size, birds – six orders of magnitude). This development was further facilitated by the cooling of the planet at that time.

Endothermy has formed in birds and mammals independently and in different morpho-physiological bases. However, in both groups, endothermy originated as an effect of selection for aerobic metabolism improvement, that provided a higher level of activity. As a result, these groups developed a basal metabolic rate that increased over time. The advantages of having a high and stable body temperature, which is inevitably related to metabolism intensification, led to the development of thermoregulatory adaptations, such as fur and feathers. Thus, metabolic heat production may be retained and heat absorption may be reduced in hot environments. The emergence of endothermy with an aerobic supply of motion activity, which regulates the level of metabolism and heat loss, has created many opportunities for endothermic animals. Achieving such a level of energy utilisation has allowed these animals to maintain activity for a long period, whereas its sensory support led to the complication and diversification of the behavioural repertoire of birds and mammals. All of these adaptations lead to the penetration of animals into those places on the planet that were previously unsuitable for them.

## ﻿Conclusions

Our analysis of the most complete data on standardised energetic costs of endothermic animals, available from the literature based on 1817 measurements of species (817 data points from mammals and 1000 data points from birds). We analysed factors that are responsible for energy expenditure by birds and mammals, and look for important life history aspects that are common for a large number of species. Ecological and behavioural factors that govern BMR in birds and mammals are nearly identical, even though each of the two clades possessed some specific features that influenced their energetics, including evolution of endothermy and flight in birds, and endothermy and viviparity in mammals. We used these data to estimate scaling coefficients and intercepts for the main groups. In all groups, BMR varies with body size but with significantly different intercepts. We provide two ways to compare BMR levels, regardless of body size. We determined the common scaling slope for all groups, recalculated the original data with this slope, and obtained the new intercept values, which were then correlated with the intercept to passerines that have the highest BMR and obtained the dimensionless BMR ratio for the selected groups. BMR in groups increase and group exponents decrease as group divergence nears present times while with increase metabolic rate during activity, group scaling exponents not only do not decrease but can increase. We considered BMR variation and the duration of activity in three mammalian subclasses: monotremes, marsupials and eutherians, and in three groups of birds: Palaeognathae, non-Passeriformes and Passeriformes, depending on the evolutionary age of these groups. Activity duration varies between the main groups of endotherms. A high level of activity is related to high BMR. Eutheria and Palaeognathes have similar BMR, i.e., terrestrial lifestyle without flight is based on nearly equal BMR and these groups evolved at practically almost the same time. We calculated sleep duration in the main groups of endotherms on the basis of data from the literature. BMR in a taxon correlates with its evolutionary age: the later a clade diverged, the higher is its metabolic rate and the longer is its activity period. We suggest that each group formed its taxon-specific BMR, depending on the ability to maintain thermal homeostasis under the environmental conditions that prevailed during its emergence. Monotremes were the first to branch off from the basal mammals and have the lowest BMR and the lowest *T*_B_. The next level allows marsupials to maintain thermal homeostasis under a broader range of conditions, and have a more protracted period of activity. Finally, the metabolic rate and *T*_B_ typical of placentals and Palaeognathae formed in the mid Cretaceous, and allowed these groups to occupy a broader range of terrestrial niches. Immediately when the development of blood circulation and respiratory systems made it possible to reach the BMR that allowed maintaining *T*_B_ of 37 °C, the explosive radiation of mammals and birds started. In the mid and late Cretaceous, birds and mammals started to occupy the leading positions in the ecosystems. Further, at last, some 50 mya passerines that have the highest BMR (nearly 50% higher than eutherians and palaeognath birds), adapted to the forest habitats and gained *T*_B_ of ca. 40 °C, which is at the upper physiological limit. The duration of activity grew in parallel to the BMR. Ecological expansion of birds and mammals resulted in their worldwide geographic distribution.

## Supplementary materials

Supplementary material 1 Mammalian Basal metabolic rate (BMR) dafrom Genoud et al. (2017) Data type table (excel document) https://binary.pensoft.net/file/808699This dataset is made available under the Open Database License (http://opendatacommons.org/licenses/odbl/1.0/). The Open Database License (ODbL) is a license agreement intended to allow users to freely share, modify, and use this Dataset while maintaining this same freedom for others, provided that the original source and author(s) are credited.Valery M. Gavrilov, Tatiana B. Golubeva, Andrey V. Bushuev

Supplementary material 2
Aves BMR
Data type table (PDF file) https://binary.pensoft.net/file/808700This dataset is made available under the Open Database License (http://opendatacommons.org/licenses/odbl/1.0/). The Open Database License (ODbL) is a license agreement intended to allow users to freely share, modify, and use this Dataset while maintaining this same freedom for others, provided that the original source and author(s) are credited.Valery M. Gavrilov, Tatiana B. Golubeva, Andrey V. Bushuev

Supplementary material 3 Sleep duration for endothermic species (by review Cambell & Tobler, 1984) and others Data type table (PDF file) https://binary.pensoft.net/file/808701This dataset is made available under the Open Database License (http://opendatacommons.org/licenses/odbl/1.0/). The Open Database License (ODbL) is a license agreement intended to allow users to freely share, modify, and use this Dataset while maintaining this same freedom for others, provided that the original source and author(s) are credited.Valery M. Gavrilov, Tatiana B. Golubeva, Andrey V. Bushuev
