# Supplementary material for: ﻿Metabolic rate, sleep duration, and body temperature in evolution of mammals and birds: the influence of geological time of principal groups divergence
Source: Zookeys. 2023 Feb 14;1148:1–27. doi: 10.3897/zookeys.1148.93458 (PMC10208811; doi:10.3897/zookeys.1148.93458)
Supplement: Supplementary material 2 — Aves BMR [file zookeys-1148-001_article-93458__-s002.pdf]

Supplementary information Table 2

Avian BMR

Class	HighTaxon	Taxon	Species	body mass, g	BMR, mlO_2/h	References
Aves	Paleognathae	Apterygi	*Apteryx australis*	3137.00	830.00	McNab 1996
Aves	Paleognathae	Apterygi	*Apteryx haastii*	2529.00	951.00	McNab 1996
Aves	Paleognathae	Apterygi	*Apteryx owenii*	1377.00	710.50	McNab 1996
Aves	Paleognathae	Casuari	*Casuarius bennetti*	17600.00	4501.00	Benedict, Fox 1927
Aves	Paleognathae	Casuari	*Dromaius novaehollandiae*	38900.00	6302.00	Maloney & Dawson 1994
Aves	Paleognathae	Rhea	*Rhea americana*	21700.00	6900.00	Grawford, Lasiewski 1968
Aves	Paleognathae	Struthio	*Struthio camelus*	92400.00	10483.00	Withers 1983
Aves	Paleognathae	Tinami	*Crypturellus soui*	220.80	140.00	Londono et al. 2015
Aves	Paleognathae	Tinami	*Nothoprocta perdicaria*	458.00	317.50	Withers et al. 1987
Aves	Non-Passer	Accipitri	*Accipiter cooperii*	452.00	383.00	Wasser 1986
Aves	Non-Passer	Accipitri	*Accipiter nisus*	135.00	171.00	Kendeigh et al. 1977
Aves	Non-Passer	Accipitri	*Accipiter striatus*	173.40	94.55	Londono et al. 2015
Aves	Non-Passer	Accipitri	*Accipiter striatus*	83.00	126.00	Wasser 1986
Aves	Non-Passer	Accipitri	*Aquila chrysaetos*	3000.00	890.00	Giaja, Males 1928
Aves	Non-Passer	Accipitri	*Aquila rapax*	2398.00	813.50	Wasser 1986
Aves	Non-Passer	Accipitri	*Buteo buteo*	1012.00	676.00	Prinzinger & Hanssler 1980
Aves	Non-Passer	Accipitri	*Buteo jamaicensis*	1475.00	571.50	Wasser 1986
Aves	Non-Passer	Accipitri	*Buteo lineatus*	658.00	380.00	Wasser 1986
Aves	Non-Passer	Accipitri	*Geranoaetus melanoleucus*	2860.00	924.50	Benedict, Fox 1927
Aves	Non-Passer	Accipitri	*Gypaetus barbatus*	5070.00	1989.00	Benedict, Fox 1927_
Aves	Non-Passer	Accipitri	*Ictinia mississippiensis*	232.00	164.50	Wasser 1986
Aves	Non-Passer	Accipitri	*Pandion haliaetus*	1495.00	994.00	Wasser 1986
Aves	Non-Passer	Accipitri	*Parabuteo unicinctus*	572.00	253.50	Wasser 1986
Aves	Non-Passer	Accipitri	*Pernis apivorus*	652.00	420.50	Kendeigh et al. 1977
Aves	Non-Passer	Anseri	*Aix sponsa*	448.00	404.50	Kendeigh et al. 1977
Aves	Non-Passer	Anseri	*Anas acuta*	721.00	785.50	Prinzinger & Hanssler 1980
Aves	Non-Passer	Anseri	*Anas aucklandica*	373.10	337.50	McNab 2003
Aves	Non-Passer	Anseri	*Anas castanea*	483.30	343.00	McNab 2003
Aves	Non-Passer	Anseri	*Anas chlorotis*	528.80	417.50	McNab 2003
Aves	Non-Passer	Anseri	*Anas crecca*	250.00	300.00	Prinzinger & Hanssler 1980
Aves	Non-Passer	Anseri	*Anas gracilis*	393.70	599.00	McNab 2003
Aves	Non-Passer	Anseri	*Anas nesiotis*	371.10	297.00	McNab 2003
Aves	Non-Passer	Anseri	*Anas platyrhynchos*	1020.00	732.00	Kendeigh et al. 1977
Aves	Non-Passer	Anseri	*Anser anser*	3250.00	1950.50	Kendeigh et al. 1977
Aves	Non-Passer	Anseri	*Anser caerulescens*	2930.00	1395.00	Boisemena et al. 1992
Aves	Non-Passer	Anseri	*Anser canagicus*	2609.00	1612.50	Gavrilov & Dolnik 1985
Aves	Non-Passer	Anseri	*Aythya affinis*	561.50	516.50	McNab 2003
Aves	Non-Passer	Anseri	*Aythya collaris*	681.50	592.00	McNab 2003
Aves	Non-Passer	Anseri	*Aythya ferina*	816.00	1047.00	Prinzinger & Hanssler 1980
Aves	Non-Passer	Anseri	*Aythya fuligula*	574.00	486.50	Prinzinger & Hanssler 1980
Aves	Non-Passer	Anseri	*Aythya novaeseelandiae*	488.40	420.00	McNab 2003
Aves	Non-Passer	Anseri	*Aythya nyroca*	440.00	589.00	Prinzinger & Hanssler 1980
Aves	Non-Passer	Anseri	*Branta bernicla*	1253.00	1091.00	Daan et al. 1990
Aves	Non-Passer	Anseri	*Chauna chavaria*	2620.00	1238.50	Benedict, Fox, 1927
Aves	Non-Passer	Anseri	*Cygnus buccinator*	8800.00	3646.50	Benedict, Fox, 1927
Aves	Non-Passer	Anseri	*Cygnus olor*	8538.00	5606.00	Bech 1980
Aves	Non-Passer	Anseri	*Hymenolaimus malacorhynchos*	717.10	565.50	McNab 2003
Aves	Non-Passer	Anseri	*Lophodytes cucullatus*	413.30	351.50	McNab 2003

Aves	Non-Passer	Anseri	*Mareca penelope*	723.00	507.50	Kendeigh et al. 1977
Aves	Non-Passer	Anseri	*Mareca strepera*	791.00	1116.50	Prinzinger & Hanssler 1980
Aves	Non-Passer	Anseri	*Netta rufina*	1237.00	1278.00	Prinzinger & Hanssler 1980
Aves	Non-Passer	Anseri	*Somateria mollissima*	1660.00	1099.50	Jenssen et al. 1989
Aves	Non-Passer	Anseri	*Spatula clypeata*	554.00	698.00	Prinzinger & Hanssler 1980
Aves	Non-Passer	Anseri	*Spatula querquedula*	289.00	402.00	Prinzinger & Hanssler1980
Aves	Non-Passer	Anseri	*Spatula rhynchotis*	508.00	473.50	McNab 2003
Aves	Non-Passer	Anseri	*Tadorna variegata*	1193.60	602.00	McNab 2003
Aves	Non-Passer	Apodi	*Adelomyia melanogenys*	3.50	14.55	Londono et al. 2015
Aves	Non-Passer	Apodi	*Aerodramus germani*	10.80	40.04	Bushuev et al. (unpublished)
Aves	Non-Passer	Apodi	*Aerodramus vanikorensis*	11.60	21.50	McNab & Bonaccorso 1995
Aves	Non-Passer	Apodi	*Aglaeactis castelnaudii*	6.90	18.18	Londono et al. 2015
Aves	Non-Passer	Apodi	*Aglaeactis cupripennis*	7.20	21.82	Londono et al. 2015
Aves	Non-Passer	Apodi	*Apus apus*	44.90	78.50	Kendeigh et al. 1977
Aves	Non-Passer	Apodi	*Archilochus alexandri*	3.30	11.50	Lassiewski, 1963
Aves	Non-Passer	Apodi	*Boissonneaua matthewsii*	7.50	36.36	Londono et al., 2015
Aves	Non-Passer	Apodi	*Calypte anna*	4.80	21.00	Lassiewski 1963
Aves	Non-Passer	Apodi	*Calypte costae*	3.20	9.50	Lassiewski 1963
Aves	Non-Passer	Apodi	*Chaetocercus mulsant*	3.60	12.73	Londono et al. 2015
Aves	Non-Passer	Apodi	*Chalcostigma ruficeps*	3.60	27.27	Londono et al., 2015
Aves	Non-Passer	Apodi	*Coeligena coeligena*	7.10	34.55	Londono et al. 2015
Aves	Non-Passer	Apodi	*Coeligena violifer*	7.80	25.45	Londono et al. 2015
Aves	Non-Passer	Apodi	*Colibri coruscans*	8.40	27.27	Londono et al. 2015
Aves	Non-Passer	Apodi	*Colibri thalassinus*	6.20	29.09	Londono et al., 2015
Aves	Non-Passer	Apodi	*Collocalia esculenta*	6.80	14.50	McNab & Bonaccorso 1995
Aves	Non-Passer	Apodi	*Doryfera ludovicae*	5.90	29.09	Londono et al. 2015
Aves	Non-Passer	Apodi	*Eugenes fulgens*	6.60	18.50	Lassiewski, Lassiewski 1963
Aves	Non-Passer	Apodi	*Eulampis jugularis*	8.40	69.50	Hainsworth, Wolf 1970
Aves	Non-Passer	Apodi	*Eutoxeres condamini*	10.00	40.00	Londono et al. 2015
Aves	Non-Passer	Apodi	*Glaucis hirsutus*	6.90	32.73	Londono et al. 2015
Aves	Non-Passer	Apodi	*Haplophaedia aureliae*	4.80	25.45	Londono et al. 2015
Aves	Non-Passer	Apodi	*Heliangelus amethysticollis*	5.80	27.27	Londono et al. 2015
Aves	Non-Passer	Apodi	*Heliodoxa leadbeateri*	7.50	34.55	Londono et al. 2015
Aves	Non-Passer	Apodi	*Lafresnaya lafresnayi*	5.70	25.45	Londono et al. 2015
Aves	Non-Passer	Apodi	*Lampornis clemenciae*	7.90	18.50	Lassiewski, Lassiewski 1963
Aves	Non-Passer	Apodi	*Metallura tyrianthina*	3.80	18.18	Londono et al. 2015
Aves	Non-Passer	Apodi	*Ocreatus underwoodii*	3.30	14.55	Londono et al. 2015
Aves	Non-Passer	Apodi	*Oreotrochilus estella*	8.40	34.50	Carpenter 1976
Aves	Non-Passer	Apodi	*Patagona gigas*	19.10	51.50	Lasiewski et al. 1967
Aves	Non-Passer	Apodi	*Phaethornis guy*	5.90	23.64	Londono et al. 2015
Aves	Non-Passer	Apodi	*Phaethornis hispidus*	4.90	25.45	Londono et al. 2015
Aves	Non-Passer	Apodi	*Phaethornis koepckeae*	5.40	23.64	Londono et al. 2015
Aves	Non-Passer	Apodi	*Phaethornis superciliosus*	5.20	25.45	Londono et al. 2015
Aves	Non-Passer	Apodi	*Phlogophilus harterti*	2.80	27.27	Londono et al. 2015
Aves	Non-Passer	Apodi	*Pterophanes cyanopterus*	10.80	20.00	Londono et al. 2015
Aves	Non-Passer	Apodi	*Ramphomicron microrhynchum*	3.90	14.55	Londono et al. 2015
Aves	Non-Passer	Apodi	*Schistes geoffroyi*	4.80	32.73	Londono et al. 2015
Aves	Non-Passer	Apodi	*Selasphorus calliope*	3.00	12.00	Lasiewski 1963
Aves	Non-Passer	Apodi	*Selasphorus rufus*	3.80	12.50	Lassiewski 1963
Aves	Non-Passer	Apodi	*Selasphorus sasin*	3.70	12.50	Lassiewski 1963
Aves	Non-Passer	Apodi	*Sephanoides sephaniodes*	5.70	18.50	Lopez-Calleja & Bozinovic 1995

Aves	Non-Passer	Apodi	*Streptoprocne rutila*	16.10	43.64	Londono et al. 2015
Aves	Non-Passer	Apodi	*Taphrospilus hypostictus*	7.50	23.64	Londono et al. 2015
Aves	Non-Passer	Apodi	*Thalurania furcata*	3.80	23.64	Londono et al. 2015
Aves	Non-Passer	Apodi	*Threnetes leucurus*	9.40	27.27	Londono et al. 2015
Aves	Non-Passer	Caprimulgi	*Caprimulgus europaeus*	77.40	116.00	Gavrilov, Dolnik 1985
Aves	Non-Passer	Caprimulgi	*Caprimulgus macrurus*	73.43	73.49	Bushuev et al. (unpublished)
Aves	Non-Passer	Caprimulgi	*Caprimulgus macrurus*	68.60	56.00	McNab & Bonaccorso 1995
Aves	Non-Passer	Caprimulgi	*Chordeiles minor*	72.00	79.50	Lasiewski & Dawson 1964
Aves	Non-Passer	Caprimulgi	*Eurostopodus argus*	88.00	73.50	Dawson & Fisher 1969
Aves	Non-Passer	Caprimulgi	*Eurostopodus argus*	88.00	73.50	Dawson & Fisher 1969
Aves	Non-Passer	Caprimulgi	*Eurostopodus mystacalis*	162.00	86.50	McNab & Bonaccorso 1995
Aves	Non-Passer	Caprimulgi	*Lyncornis macrotis*	162.80	147.40	Bushuev et al. (unpublished)
Aves	Non-Passer	Caprimulgi	*Nyctidromus albicollis*	43.00	83.00	Scholander et al.1950
Aves	Non-Passer	Caprimulgi	*Phalaenoptilus nuttallii*	40.00	32.00	Bartholomew et al. 1962
Aves	Non-Passer	Caprimulgi	*Podargus ocellatus*	145.00	102.00	Lasiewski et al. 1970
Aves	Non-Passer	Caprimulgi	*Podargus papuensis*	314.60	192.50	McNab & Bonaccorso 1995
Aves	Non-Passer	Caprimulgi	*Podargus strigoides*	380.30	186.00	Bech & Nichol 1999
Aves	Non-Passer	Caprimulgi	*Uropsalis lyra*	77.50	94.55	Londono et al. 2015
Aves	Non-Passer	Caprimulgi	*Uropsalis segmentata*	34.70	98.18	Londono et al. 2015
Aves	Non-Passer	Catharti	*Cathartes aura*	1100.00	515.00	Enger 1957
Aves	Non-Passer	Catharti	*Coragyps atratus*	1700.00	798.00	Enger 1957
Aves	Non-Passer	Catharti	*Coragyps atratus*	2120.00	1171.50	Larochelle et al. 1982
Aves	Non-Passer	Charadrii	*Alca torda*	589.00	648.00	Bryant & Furness 1995
Aves	Non-Passer	Charadrii	*Alle alle*	152.50	370.00	Gabrielsen et al. 1991
Aves	Non-Passer	Charadrii	*Arenaria interpres*	90.00	166.00	Kersten & Piersma 1987
Aves	Non-Passer	Charadrii	*Calidris canutus*	130.00	158.50	Piersma et al. 1995
Aves	Non-Passer	Charadrii	*Cepphus grylle*	342.20	546.00	Gabrielsen et al. 1991
Aves	Non-Passer	Charadrii	*Charadrius dubius*	36.00	75.00	Kendeigh et al. 1977
Aves	Non-Passer	Charadrii	*Chroicocephalus ridibundus*	306.00	335.00	Kendeigh et al. 1977
Aves	Non-Passer	Charadrii	*Fratercula arctica*	329.00	462.50	Bryant & Furness 1995
Aves	Non-Passer	Charadrii	*Haematopus ostralegus*	554.00	524.00	Kersten & Piersma 1987
Aves	Non-Passer	Charadrii	*Jacana jacana*	107.00	181.00	Wiersma et al. 2007
Aves	Non-Passer	Charadrii	*Larus argentatus*	924.00	891.50	Bryant & Furness 1995
Aves	Non-Passer	Charadrii	*Larus canus*	431.00	405.00	Kendeigh et al. 1977
Aves	Non-Passer	Charadrii	*Larus hyperboreus*	1600.00	2664.00	Scholander et al. 1950
Aves	Non-Passer	Charadrii	*Larus pacificus*	1210.00	1107.50	Benedict, Fox 1927
Aves	Non-Passer	Charadrii	*Limosa lapponica*	240.00	273.50	Daan et al. 1990
Aves	Non-Passer	Charadrii	*Pluvialis apricaria*	151.00	223.50	Daan et al. 1990
Aves	Non-Passer	Charadrii	*Pluvialis squatarola*	226.00	320.50	Kersten & Piersma 1987
Aves	Non-Passer	Charadrii	*Rhodostethia rosea*	155.00	191.50	Gavrilov 1996
Aves	Non-Passer	Charadrii	*Rissa tridactyla*	305.00	494.00	Bryant & Furness 1995
Aves	Non-Passer	Charadrii	*Scolopax minor*	156.70	192.50	Vander Haegen et al. 1994
Aves	Non-Passer	Charadrii	*Scolopax rusticola*	430.00	389.00	Kendeigh et al. 1977
Aves	Non-Passer	Charadrii	*Stercorarius maccormicki*	1130.00	1547.50	Ricklefs, Mattew 1983
Aves	Non-Passer	Charadrii	*Stercorarius parasiticus*	351.00	414.50	Bryant & Furness 1995
Aves	Non-Passer	Charadrii	*Stercorarius skua*	970.00	855.00	Benedict, Fox 1927
Aves	Non-Passer	Charadrii	*Stercorarius skua*	1159.00	1121.00	Bryant & Furness 1995
Aves	Non-Passer	Charadrii	*Thinocorus rumicivorus*	55.50	56.00	Ehlers & Morton 1982
Aves	Non-Passer	Charadrii	*Tringa ochropus*	90.00	166.00	Prinzinger, Hanssler 1980
Aves	Non-Passer	Charadrii	*Turnix suscitator*	66.00	73.25	Bushuev et al. (unpublished)
Aves	Non-Passer	Charadrii	*Uria aalge*	771.00	812.50	Bryant & Furness 1995

Aves	Non-Passer	Charadrii	*Uria lomvia*	819.00	912.50	Bryant & Furness 1995
Aves	Non-Passer	Ciconii	*Jabiru mycteria*	5470.00	2372.50	Benedict, Fox 1927
Aves	Non-Passer	Ciconii	*Leptoptilos javanicus*	5710.00	2678.00	Benedict, Fox 1927
Aves	Non-Passer	Ciconii	*Mycteria americana*	2500.00	1753.50	Kahl 1962
Aves	Non-Passer	Colii	*Colius castanotus*	69.00	83.00	Prinzinger et al. 1981
Aves	Non-Passer	Colii	*Colius colius*	35.10	32.00	McKechnie & Lovegrove 2001b
Aves	Non-Passer	Colii	*Colius striatus*	51.00	42.50	McKechnie & Lovegrove 2001a
Aves	Non-Passer	Colii	*Urocolius indicus*	53.50	129.00	Hoffmann, Prinzinger 1984
Aves	Non-Passer	Colii	*Urocolius macrourus*	51.30	43.00	Prinzinger 1988
Aves	Non-Passer	Columbi	*Caloenas nicobarica*	613.00	326.50	McNab 2000
Aves	Non-Passer	Columbi	*Chalcophaps indica*	115.95	132.76	Bushuev et al. (unpublished)
Aves	Non-Passer	Columbi	*Chalcophaps indica*	124.00	142.50	Schleucher & Withers 2002
Aves	Non-Passer	Columbi	*Columba leucomela*	456.00	438.50	Schleucher & Withers 2002
Aves	Non-Passer	Columbi	*Columba livia*	368.00	298.50	Gavrilov & Dolnik 1985
Aves	Non-Passer	Columbi	*Columba palumbus*	493.00	357.00	Gavrilov, Dolnik 1985
Aves	Non-Passer	Columbi	*Columba vitiensis*	467.90	260.00	McNab 2000
Aves	Non-Passer	Columbi	*Columbina inca*	41.50	47.00	MacMillen & Trost 1967
Aves	Non-Passer	Columbi	*Columbina inca*	40.50	45.50	MacMillen, Trost, 1965
Aves	Non-Passer	Columbi	*Columbina talpacoti*	45.70	74.50	Wiersma et al. 2007
Aves	Non-Passer	Columbi	*Drepanoptila holosericea*	198.00	148.50	Schluecher 2002
Aves	Non-Passer	Columbi	*Ducula pacifica*	333.40	143.00	McNab 2000
Aves	Non-Passer	Columbi	*Ducula pinon*	583.80	320.50	McNab 2000
Aves	Non-Passer	Columbi	*Ducula pistrinaria*	394.20	193.00	McNab 2000
Aves	Non-Passer	Columbi	*Ducula radiata*	333.60	253.00	McNab 2000
Aves	Non-Passer	Columbi	*Ducula rubricera*	418.80	245.50	McNab 2000
Aves	Non-Passer	Columbi	*Ducula rufigaster*	376.70	229.00	McNab 2000
Aves	Non-Passer	Columbi	*Ducula spilorrhoa*	453.60	247.00	McNab 2000
Aves	Non-Passer	Columbi	*Ducula zoeae*	456.20	306.50	McNab 2000
Aves	Non-Passer	Columbi	*Geopelia cuneata*	39.00	48.00	Schleucher & Withers 2002
Aves	Non-Passer	Columbi	*Geopelia placida*	52.00	64.00	Schleucher & Withers 2002
Aves	Non-Passer	Columbi	*Geopelia striata*	51.56	70.37	Bushuev et al. (unpublished)
Aves	Non-Passer	Columbi	*Geophaps plumifera*	81.00	71.50	Dawson, Bennett 1973
Aves	Non-Passer	Columbi	*Geophaps plumifera*	81.00	76.00	Withers & Williams 1990
Aves	Non-Passer	Columbi	*Geophaps smithii*	198.00	157.00	Schleucher & Withers 2002
Aves	Non-Passer	Columbi	*Geotrygon montana*	117.90	149.09	Londono et al. 2015
Aves	Non-Passer	Columbi	*Geotrygon montana*	107.30	121.50	Wiersma et al. 2007
Aves	Non-Passer	Columbi	*Goura cristata*	2313.40	768.00	McNab 2000
Aves	Non-Passer	Columbi	*Gymnophaps albertisii*	241.60	170.00	McNab 2000
Aves	Non-Passer	Columbi	*Hemiphaga novaeseelandiae*	435.60	339.00	McNab 2000
Aves	Non-Passer	Columbi	*Leptotila rufaxilla*	150.60	125.45	Londono et al., 2015
Aves	Non-Passer	Columbi	*Leptotila verreauxi*	131.00	159.50	Vleck & Vleck 1979
Aves	Non-Passer	Columbi	*Leucosarcia melanoleuca*	468.00	301.00	McNab 2000
Aves	Non-Passer	Columbi	*Ocyphaps lophotes*	187.00	195.50	Schleucher & Withers 2002
Aves	Non-Passer	Columbi	*Oena capensis*	36.00	44.00	Schleucher 2001
Aves	Non-Passer	Columbi	*Pampusana beccarii*	68.80	96.00	McNab, 2009
Aves	Non-Passer	Columbi	*Patagioenas leucocephala*	251.90	242.00	McNab 2000
Aves	Non-Passer	Columbi	*Phaps chalcoptera*	304.00	275.00	Schleucher & Withers 2002
Aves	Non-Passer	Columbi	*Phaps elegans*	190.00	223.00	Schleucher & Withers 2002
Aves	Non-Passer	Columbi	*Phaps histrionica*	257.00	233.50	Schleucher & Withers 2002
Aves	Non-Passer	Columbi	*Ptilinopus melanospilus*	98.00	88.00	Schleucher & Withers 2002
Aves	Non-Passer	Columbi	*Ptilinopus perlatus*	196.00	191.00	McNab 2000

Aves	Non-Passer	Columbi	*Ptilinopus superbus*	120.40	136.00	Schleucher 2002
Aves	Non-Passer	Columbi	*Spilopelia chinensis*	143.80	166.63	Bushuev et al. (unpublished)
Aves	Non-Passer	Columbi	*Spilopelia senegalensis*	108.00	152.50	Kendeigh et al. 1977
Aves	Non-Passer	Columbi	*Streptopelia decaocto*	170.00	171.00	Daan et al. 1990
Aves	Non-Passer	Columbi	*Streptopelia roseogrisea*	150.00	159.00	Benedict, Riddle 1928
Aves	Non-Passer	Columbi	*Streptopelia tranquebarica*	96.37	125.65	Bushuev et al. (unpublished)
Aves	Non-Passer	Columbi	*Streptopelia turtur*	154.00	205.00	Kendeigh et al. 1977
Aves	Non-Passer	Columbi	*Zenaida macroura*	91.40	116.50	Hudson & Brush 1964
Aves	Non-Passer	Columbi	*Zentrygon frenata*	322.40	160.00	Londono et al. 2015
Aves	Non-Passer	Coracii	*Alcedo atthis*	24.10	46.82	Bushuev et al. (unpublished)
Aves	Non-Passer	Coracii	*Alcedo atthis*	34.30	68.00	Kendeigh et al. 1977
Aves	Non-Passer	Coracii	*Alcedo meninting*	18.28	40.04	Bushuev et al. (unpublished)
Aves	Non-Passer	Coracii	*Baryphthengus martii*	127.10	100.00	Londono et al. 2015
Aves	Non-Passer	Coracii	*Ceryle rudis*	70.30	103.21	Bushuev et al. (unpublished)
Aves	Non-Passer	Coracii	*Ceyx erithaca*	12.89	30.33	Bushuev et al. (unpublished)
Aves	Non-Passer	Coracii	*Chloroceryle aenea*	11.80	31.50	Wiersma et al. 2007
Aves	Non-Passer	Coracii	*Dacelo novaeguineae*	336.00	201.00	Buttemer et al. 2003
Aves	Non-Passer	Coracii	*Halcyon coromanda*	66.50	92.17	Bushuev et al. (unpublished)
Aves	Non-Passer	Coracii	*Halcyon smyrnensis*	71.59	80.59	Bushuev et al. (unpublished)
Aves	Non-Passer	Coracii	*Lacedo pulchella*	49.28	57.00	Bushuev et al. (unpublished)
Aves	Non-Passer	Coracii	*Merops leschenaulti*	23.53	55.34	Bushuev et al. (unpublished)
Aves	Non-Passer	Coracii	*Merops viridis*	33.80	53.50	Bryant et al. 1984
Aves	Non-Passer	Coracii	*Momotus aequatorialis*	158.50	105.45	Londono et al. 2015
Aves	Non-Passer	Coracii	*Momotus momota*	123.10	94.55	Londono et al. 2015
Aves	Non-Passer	Coracii	*Momotus momota*	102.40	88.50	Wiersma et al. 2007
Aves	Non-Passer	Coracii	*Nyctyornis athertoni*	89.32	117.86	Bushuev et al. (unpublished)
Aves	Non-Passer	Coracii	*Pelargopsis capensis*	161.39	135.42	Bushuev et al. (unpublished)
Aves	Non-Passer	Coracii	*Phoeniculus purpureus*	74.10	62.50	Boix-Hinzen & Lovegrove 1998
Aves	Non-Passer	Coracii	*Rhyticeros plicatus*	1781.60	816.50	McNab 2001
Aves	Non-Passer	Coracii	*Todus mexicanus*	6.30	20.50	Merola-Zwartjes & Ligon 2000
Aves	Non-Passer	Coracii	*Upupa epops*	67.00	99.50	Prinzinger & Hanssler 1980
Aves	Non-Passer	Cuculi	*Cacomantis merulinus*	24.63	41.73	Bushuev et al. (unpublished)
Aves	Non-Passer	Cuculi	*Cacomantis sonneratii*	31.30	54.07	Bushuev et al. (unpublished)
Aves	Non-Passer	Cuculi	*Cacomantis variolosus*	23.80	22.00	Hails 1983
Aves	Non-Passer	Cuculi	*Centropus bengalensis*	104.50	101.96	Bushuev et al. (unpublished)
Aves	Non-Passer	Cuculi	*Centropus senegalensis*	175.00	271.50	Prinzinger & Hanssler 1980
Aves	Non-Passer	Cuculi	*Centropus sinensis*	240.40	200.22	Bushuev et al. (unpublished)
Aves	Non-Passer	Cuculi	*Clamator coromandus*	54.80	57.41	Bushuev et al. (unpublished)
Aves	Non-Passer	Cuculi	*Cuculus canorus*	111.60	151.00	Kendeigh et al. 1977
Aves	Non-Passer	Cuculi	*Eudynamys scolopaceus*	188.00	296.00	Prinzinger & Hanssler 1980
Aves	Non-Passer	Cuculi	*Geococcyx californianus*	294.60	227.50	Ohmart & Lasiewski 1971
Aves	Non-Passer	Cuculi	*Opisthocomus hoazin*	598.00	288.00	Grajal 1991
Aves	Non-Passer	Cuculi	*Phaenicophaeus tristis*	111.00	125.18	Bushuev et al. (unpublished)
Aves	Non-Passer	Falconi	*Daptrius ater*	362.00	216.00	Wasser 1986
Aves	Non-Passer	Falconi	*Falco mexicanus*	430.00	349.50	Wasser 1986
Aves	Non-Passer	Falconi	*Falco sparverius*	116.00	119.00	Wasser 1986
Aves	Non-Passer	Falconi	*Falco subbuteo*	208.00	233.50	Kendeigh et al. 1977
Aves	Non-Passer	Falconi	*Falco tinnunculus*	131.00	139.50	Gavrilov & Dolnik 1985
Aves	Non-Passer	Falconi	*Micrastur gilvicollis*	170.50	189.09	Londono et al., 2015
Aves	Non-Passer	Falconi	*Micrastur ruficollis*	304.10	130.91	Londono et al., 2015
Aves	Non-Passer	Falconi	*Microhierax caerulescens*	39.10	74.82	Bushuev et al. (unpublished)

Aves	Non-Passer	Gabuli	*Micromonacha lanceolata*	21.90	65.45	Londono et al. 2015
Aves	Non-Passer	Gabuli	*Notharchus macrorhynchos*	43.20	57.00	Wiersma et al. 2007
Aves	Non-Passer	Galli	*Alectoris chukar*	475.00	353.00	Marder & Bernstein 1983
Aves	Non-Passer	Galli	*Alectoris graeca*	633.00	456.00	Kendeigh et al. 1977
Aves	Non-Passer	Galli	*Arborophila chloropus*	251.90	200.88	Bushuev et al. (unpublished)
Aves	Non-Passer	Galli	*Bonasa umbellus*	644.00	429.00	Rasmussen, Brander 1973
Aves	Non-Passer	Galli	*Callipepla californica*	137.10	138.00	Hudson & Brush 1964
Aves	Non-Passer	Galli	*Callipepla gambelii*	126.10	136.00	Weathers 1981
Aves	Non-Passer	Galli	*Colinus virginianus*	194.00	200.50	Lasiewski, Dawson 1967
Aves	Non-Passer	Galli	*Coturnix coturnix*	97.00	160.50	Kendeigh et al. 1977
Aves	Non-Passer	Galli	*Coturnix japonica*	115.00	176.00	Prinzinger & Hanssler 1980
Aves	Non-Passer	Galli	*Coturnix pectoralis*	95.80	114.50	Roberts & Baudinette 1986
Aves	Non-Passer	Galli	*Crax alberti*	2800.00	1186.50	Benedict, Fox 1927
Aves	Non-Passer	Galli	*Dendragapus obscurus*	1131.00	892.50	Pekins et al. 1992
Aves	Non-Passer	Galli	*Excalfactoria chinensis*	44.90	66.00	Roberts & Baudinette 1986
Aves	Non-Passer	Galli	*Francolinus pintadeanus*	286.80	166.21	Bushuev et al. (unpublished)
Aves	Non-Passer	Galli	*Gallus gallus*	835.30	566.04	Bushuev et al. (unpublished)
Aves	Non-Passer	Galli	*Lagopus lagopus*	658.00	505.00	Mortensen & Blix 1986
Aves	Non-Passer	Galli	*Lagopus leucura*	326.00	425.00	Johnson 1968
Aves	Non-Passer	Galli	*Lagopus muta*	465.00	493.00	Mortensen & Blix 1986
Aves	Non-Passer	Galli	*Odontophorus stellatus*	296.50	209.09	Londono et al. 2015
Aves	Non-Passer	Galli	*Penelope purpurascens*	2040.00	977.00	Benedict, Fox 1927
Aves	Non-Passer	Galli	*Perdix perdix*	501.00	387.50	Kendeigh et al. 1977
Aves	Non-Passer	Galli	*Tetrao urogallus*	3900.00	2146.00	Kendeigh et al. 1977
Aves	Non-Passer	Grui	*Amaurornis phoenicurus*	195.20	142.85	Bushuev et al. (unpublished)
Aves	Non-Passer	Grui	*Antigone canadensis*	3890.00	1465.50	Benedict, Fox 1927
Aves	Non-Passer	Grui	*Aramides cajaneus*	374.30	277.50	McNab & Ellis 2006
Aves	Non-Passer	Grui	*Atlantisia rogersi*	39.40	40.50	Ryan et al. 1989
Aves	Non-Passer	Grui	*Crex crex*	96.00	141.50	Kendeigh et al. 1977
Aves	Non-Passer	Grui	*Fulica atra*	412.00	368.00	Kendeigh et al. 1977
Aves	Non-Passer	Grui	*Gallinula tenebrosa*	512.40	421.00	McNab & Ellis 2006
Aves	Non-Passer	Grui	*Gallirallus australis*	813.50	329.00	McNab & Ellis 2006
Aves	Non-Passer	Grui	*Gallirallus owstoni*	198.80	165.00	McNab & Ellis 2006
Aves	Non-Passer	Grui	*Gallirallus philippensis*	171.70	175.50	McNab & Ellis 2006
Aves	Non-Passer	Grui	*Grus paradisea*	4030.00	1919.00	Benedict, Fox, 1927
Aves	Non-Passer	Grui	*Megacrex inepta*	856.60	241.50	McNab & Ellis 2006
Aves	Non-Passer	Grui	*Porphyrio hochstetteri*	2758.30	1239.50	McNab & Ellis 2006
Aves	Non-Passer	Grui	*Porphyrio porphyrio*	919.30	665.50	McNab & Ellis 2006
Aves	Non-Passer	Grui	*Porzana cinerea*	47.90	89.00	McNab & Ellis 2006
Aves	Non-Passer	Grui	*Tribonyx mortierii*	954.40	573.50	McNab & Ellis 2006
Aves	Non-Passer	Grui	*Tribonyx ventralis*	309.20	290.50	McNab & Ellis 2006
Aves	Non-Passer	Grui	*Turnix suscitator*	58.10	69.50	Prinzinger et al. 1993
Aves	Non-Passer	Pelecani	*Ardea herodias*	1870.00	1116.50	Benedict, Fox 1927
Aves	Non-Passer	Pelecani	*Ardeola bacchus*	202.40	176.63	Bushuev et al. (unpublished)
Aves	Non-Passer	Pelecani	*Botaurus lentiginosus*	600.00	488.50	Benedict, Fox 1927
Aves	Non-Passer	Pelecani	*Bubulcus ibis*	299.20	237.00	Ellis 1980
Aves	Non-Passer	Pelecani	*Egretta caerulea*	290.30	179.50	Ellis 1980
Aves	Non-Passer	Pelecani	*Egretta thula*	314.00	244.50	Ellis 1980
Aves	Non-Passer	Pelecani	*Egretta tricolor*	309.00	306.00	Ellis 1980
Aves	Non-Passer	Pelecani	*Eudocimus albus*	940.00	741.50	Benedict, Fox 1927
Aves	Non-Passer	Pelecani	*Ixobrychus cinnamomeus*	121.60	115.26	Bushuev et al. (unpublished)

Aves	Non-Passer	Pelecani	*Ixobrychus sinensis*	70.10	61.64	Bushuev et al. (unpublished)
Aves	Non-Passer	Pelecani	*Pelecanus conspicillatus*	5090.00	3262.50	Benedict, Fox 1927
Aves	Non-Passer	Pelecani	*Pelecanus occidentalis*	3510.00	2303.00	Benedict, Fox 1927
Aves	Non-Passer	Phoenicop	*Phoenicopterus roseus*	3040.00	1875.50	Benedict, Fox 1927
Aves	Non-Passer	Pici	*Andigena hypoglauca*	282.90	310.91	Londono et al. 2015
Aves	Non-Passer	Pici	*Aulacorhynchus prasinus*	137.00	178.18	Londono et al. 2015
Aves	Non-Passer	Pici	*Aulacorhynchus prasinus*	174.70	149.50	McNab 2001
Aves	Non-Passer	Pici	*Aulacorhynchus sulcatus*	131.70	122.00	McNab 2001
Aves	Non-Passer	Pici	*Bucco macrodactylus*	23.70	32.73	Londono et al., 2015
Aves	Non-Passer	Pici	*Celeus loricatus*	83.10	123.50	Wiersma et al. 2007
Aves	Non-Passer	Pici	*Celeus spectabilis*	113.00	178.18	Londono et al. 2015
Aves	Non-Passer	Pici	*Chrysocolaptes lucidus*	146.45	185.23	Bushuev et al. (unpublished)
Aves	Non-Passer	Pici	*Colaptes rivolii*	81.50	169.09	Londono et al., 2015
Aves	Non-Passer	Pici	*Dendrocopos major*	98.00	161.00	Kendeigh et al. 1977
Aves	Non-Passer	Pici	*Dinopium javanense*	85.14	123.65	Bushuev et al. (unpublished)
Aves	Non-Passer	Pici	*Dryobates pubescens*	23.30	59.50	Liknes & Swanson 1996
Aves	Non-Passer	Pici	*Galbula cyanescens*	24.00	41.82	Londono et al. 2015
Aves	Non-Passer	Pici	*Gecinulus grantia*	72.10	104.16	Bushuev et al. (unpublished)
Aves	Non-Passer	Pici	*Hemicircus canente*	41.05	77.98	Bushuev et al. (unpublished)
Aves	Non-Passer	Pici	*Jynx torquilla*	31.80	64.50	Kendeigh et al. 1977
Aves	Non-Passer	Pici	*Malacoptila fulvogularis*	49.60	96.36	Londono et al. 2015
Aves	Non-Passer	Pici	*Malacoptila semicincta*	47.10	60.00	Londono et al. 2015
Aves	Non-Passer	Pici	*Melanerpes rubricapillus*	48.70	96.50	Wiersma et al. 2007
Aves	Non-Passer	Pici	*Micropternus brachyurus*	79.37	114.30	Bushuev et al. (unpublished)
Aves	Non-Passer	Pici	*Monasa morphoeus*	67.50	81.82	Londono et al. 2015
Aves	Non-Passer	Pici	*Monasa nigrifrons*	86.00	83.64	Londono et al. 2015
Aves	Non-Passer	Pici	*Picumnus rufiventris*	21.90	47.27	Londono et al. 2015
Aves	Non-Passer	Pici	*Picus canus*	152.13	183.98	Bushuev et al. (unpublished)
Aves	Non-Passer	Pici	*Picus chlorolophus*	62.00	91.06	Bushuev et al. (unpublished)
Aves	Non-Passer	Pici	*Picus vittatus*	119.75	155.55	Bushuev et al. (unpublished)
Aves	Non-Passer	Pici	*Psilopogon australis*	28.30	61.70	Bushuev et al. (unpublished)
Aves	Non-Passer	Pici	*Psilopogon faiostrictus*	75.30	166.85	Bushuev et al. (unpublished)
Aves	Non-Passer	Pici	*Psilopogon lineatus*	116.70	131.65	Bushuev et al. (unpublished)
Aves	Non-Passer	Pici	*Pteroglossus aracari*	200.70	160.00	McNab 2001
Aves	Non-Passer	Pici	*Pteroglossus bailloni*	133.00	126.50	McNab 2001
Aves	Non-Passer	Pici	*Ramphastos dicolorus*	328.90	227.50	McNab 2001
Aves	Non-Passer	Pici	*Ramphastos toco*	582.00	436.00	McNab 2001
Aves	Non-Passer	Pici	*Ramphastos tucanus*	420.30	307.50	McNab 2001
Aves	Non-Passer	Pici	*Sasia ochracea*	8.35	33.59	Bushuev et al. (unpublished)
Aves	Non-Passer	Pici	*Selenidera reinwardtii*	173.60	218.18	Londono et al. 2015
Aves	Non-Passer	Pici	*Trachyphonus darnaudii*	36.60	39.50	McNab 2001
Aves	Non-Passer	Pici	*Veniliornis nigriceps*	43.40	89.09	Londono et al., 2015
Aves	Non-Passer	Podicipedi	*Podiceps nigricollis*	317.00	385.50	Ellis & Jehl 2003
Aves	Non-Passer	Procellarii	*Aphrodroma brevirostris*	315.00	319.00	Brown & Adams 1984
Aves	Non-Passer	Procellarii	*Diomedea exulans*	8130.00	3658.50	Brown & Adams 1984
Aves	Non-Passer	Procellarii	*Fulmarus glacialis*	728.00	687.50	Bryant & Furness 1995
Aves	Non-Passer	Procellarii	*Halobaena caerulea*	191.00	319.00	Brown 1988
Aves	Non-Passer	Procellarii	*Macronectes giganteus*	4780.00	2404.00	Brown & Adams 1984
Aves	Non-Passer	Procellarii	*Oceanodroma furcata*	44.60	79.50	Vleck & Kenagy 1980
Aves	Non-Passer	Procellarii	*Oceanodroma leucorhoa*	42.00	110.50	Iversen, Krog, 1972; Ricklefs et 1980

Aves	Non-Passer	Procellarii	*Pelecanoides georgicus*	127.00	177.00	Adams, Brown 1984
Aves	Non-Passer	Procellarii	*Phoebastria immutabilis*	2522.00	1345.00	Grant, Whittow 1983
Aves	Non-Passer	Procellarii	*Phoebetria fusca*	2875.00	1489.50	Adams & Brown 1984
Aves	Non-Passer	Procellarii	*Procellaria aequinoctialis*	1287.00	1135.50	Brown & Adams 1984
Aves	Non-Passer	Procellarii	*Procellaria cinerea*	1014.00	902.00	Brown & Adams 1984
Aves	Non-Passer	Procellarii	*Pterodroma hypoleuca*	169.00	228.00	Grant, Whittow 1983
Aves	Non-Passer	Procellarii	*Pterodroma macroptera*	479.00	485.50	Brown 1988
Aves	Non-Passer	Procellarii	*Pterodroma mollis*	274.00	314.50	Adams & Brown 1984
Aves	Non-Passer	Procellarii	*Puffinus puffinus*	367.00	419.00	Bryant & Furness 1995
Aves	Non-Passer	Procellarii	*Thalassarche chrysostoma*	3753.00	1531.50	Adams & Brown 1984
Aves	Non-Passer	Psittaci	*Agapornis fischeri*	56.70	95.00	Gavrilov & Dolnik 1985
Aves	Non-Passer	Psittaci	*Agapornis roseicollis*	48.10	83.50	Kendeigh et al. 1977
Aves	Non-Passer	Psittaci	*Barnardius zonarius*	131.80	142.50	Williams et al. 1991
Aves	Non-Passer	Psittaci	*Bolborhynchus lineola*	54.50	106.50	Bucher 1981
Aves	Non-Passer	Psittaci	*Bolborhynchus orbygnesius*	46.40	69.09	Londono et al. 2015
Aves	Non-Passer	Psittaci	*Brotogeris jugularis*	63.50	103.50	Wiersma et al. 2007
Aves	Non-Passer	Psittaci	*Cyanoramphus auriceps*	52.90	88.50	McNab & Salisbury 1995
Aves	Non-Passer	Psittaci	*Cyanoramphus novaezelandiae*	56.10	112.00	McNab & Salisbury 1995
Aves	Non-Passer	Psittaci	*Cyanoramphus unicolor*	129.40	194.50	McNab & Salisbury 1995
Aves	Non-Passer	Psittaci	*Loriculus galgulus*	27.00	93.00	Prinzinger & Hanssler 1980
Aves	Non-Passer	Psittaci	*Loriculus vernalis*	26.70	58.45	Bushuev et al. (unpublished)
Aves	Non-Passer	Psittaci	*Melopsittacus undulatus*	33.70	66.50	Weathers & Schoenbaechler 1[illegible]
Aves	Non-Passer	Psittaci	*Myiopsitta monachus*	80.40	94.50	Weathers & Caccamise 1978
Aves	Non-Passer	Psittaci	*Neophema petrophila*	48.40	114.00	Williams et al. 1991
Aves	Non-Passer	Psittaci	*Neophema pulchella*	40.00	104.50	Prinzinger & Hanssler 1980
Aves	Non-Passer	Psittaci	*Neopsephotus bourkii*	40.00	97.00	Prinzinger & Hanssler 1980
Aves	Non-Passer	Psittaci	*Psittacula alexandri*	114.80	151.17	Bushuev et al. (unpublished)
Aves	Non-Passer	Psittaci	*Trichoglossus haematodus*	137.10	215.00	Williams et al. 1991
Aves	Non-Passer	Pteroclidi	*Pterocles orientalis*	386.40	350.50	Hinsley et al. 1993
Aves	Non-Passer	Sphenisci	*Aptenodytes forsteri*	23370.00	7709.50	Pinshow et al. 1976
Aves	Non-Passer	Sphenisci	*Aptenodytes patagonicus*	11080.00	3933.50	Gavrilov 1977
Aves	Non-Passer	Sphenisci	*Eudyptes chrysocome*	2330.00	1050.00	Gavrilov 1977; Brown 1984
Aves	Non-Passer	Sphenisci	*Eudyptes chrysolophus*	3870.00	1556.50	Gavrilov 1977; Brown 1984
Aves	Non-Passer	Sphenisci	*Eudyptes pachyrhynchus*	2600.00	1246.00	Drent & Stonehouse 1971
Aves	Non-Passer	Sphenisci	*Eudyptula minor*	1100.00	574.50	Baudinette et al. 1986
Aves	Non-Passer	Sphenisci	*Eudyptula minor*	1150.00	1190.50	Pinshow et al. 1977
Aves	Non-Passer	Sphenisci	*Megadyptes antipodes*	4800.00	2072.50	Drent & Stonehouse 1971
Aves	Non-Passer	Sphenisci	*Pygoscelis adeliae*	3970.00	2202.00	Kooyman et al. 1976
Aves	Non-Passer	Sphenisci	*Pygoscelis papua*	5775.00	5950.50	Scholander et al. 1940
Aves	Non-Passer	Sphenisci	*Spheniscus humboldti*	3870.00	1707.00	Drent & Stonehouse 1971
Aves	Non-Passer	Strigi	*Aegolius acadicus*	118.10	112.50	Ligon 1969
Aves	Non-Passer	Strigi	*Aegolius funereus*	130.00	203.50	Hohtola et al. 1994
Aves	Non-Passer	Strigi	*Asio flammeus*	406.00	309.50	Graber 1962
Aves	Non-Passer	Strigi	*Asio otus*	252.00	171.50	Wijnandts 1984
Aves	Non-Passer	Strigi	*Athene cunicularia*	146.70	140.00	Coulombe 1970
Aves	Non-Passer	Strigi	*Athene cunicularia*	132.40	139.50	Coulombe 1970
Aves	Non-Passer	Strigi	*Bubo scandiacus*	2026.00	592.00	Gessaman 1972
Aves	Non-Passer	Strigi	*Bubo virginianus*	1000.00	781.00	Ganey et al. 1993
Aves	Non-Passer	Strigi	*Glaucidium brasilianum*	60.60	78.18	Londono et al. 2015
Aves	Non-Passer	Strigi	*Glaucidium cuculoides*	129.90	99.31	Bushuev et al. (unpublished)
Aves	Non-Passer	Strigi	*Glaucidium cuculoides*	163.00	156.50	Johnson, Collins 1975

Aves	Non-Passer	Strigi	*Glaucidium gnoma*	52.00	83.50	Ligon 1969
Aves	Non-Passer	Strigi	*Glaucidium jardinii*	60.60	123.64	Londono et al. 2015
Aves	Non-Passer	Strigi	*Megascops asio*	165.50	108.00	Ligon 1969
Aves	Non-Passer	Strigi	*Megascops trichopsis*	99.80	75.00	Ligon 1969
Aves	Non-Passer	Strigi	*Micrathene whitneyi*	45.00	45.50	Ligon 1968
Aves	Non-Passer	Strigi	*Ninox scutulata*	187.45	166.38	Bushuev et al. (unpublished)
Aves	Non-Passer	Strigi	*Ninox theomacha*	151.40	188.50	McNab, pers. obs.
Aves	Non-Passer	Strigi	*Otus lettia*	108.90	102.41	Bushuev et al. (unpublished)
Aves	Non-Passer	Strigi	*Otus scops*	78.90	85.00	Gavrilov & Dol'nik 1985
Aves	Non-Passer	Strigi	*Otus sunia*	76.70	74.48	Bushuev et al. (unpublished)
Aves	Non-Passer	Strigi	*Strix aluco*	520.00	375.00	Herzog 1930
Aves	Non-Passer	Strigi	*Strix occidentalis*	571.00	481.50	Ganey et al. 1993
Aves	Non-Passer	Strigi	*Surnia ulula*	333.00	306.50	Johnson, Collins 1975
Aves	Non-Passer	Strigi	*Tyto alba*	533.20	323.00	Edwards 1987
Aves	Non-Passer	Strigi	*Tyto tenebricosa*	770.00	518.00	McNab 2009
Aves	Non-Passer	Strigi	*Uroglaux dimorpha*	245.30	263.50	McNab 2009
Aves	Non-Passer	Suli	*Anhinga anhinga*	1040.00	574.50	Hennemann 1983
Aves	Non-Passer	Suli	*Fregata magnificens*	1080.00	501.00	Enger, 1957
Aves	Non-Passer	Suli	*Leucocarbo atriceps*	2600.00	2693.50	Ricklefs, Mattew 1983
Aves	Non-Passer	Suli	*Morus bassanus*	2574.00	2248.00	Bryant & Furness 1995
Aves	Non-Passer	Suli	*Phalacrocorax aristotelis*	1619.00	1539.50	Bryant & Furness 1995
Aves	Non-Passer	Suli	*Phalacrocorax auritus*	1330.00	988.50	Hennemann 1983
Aves	Non-Passer	Trogoni	*Harpactes oreskios*	51.37	89.47	Bushuev et al. (unpublished)
Aves	Non-Passer	Trogoni	*Trogon personatus*	59.00	150.91	Londono et al. 2015
Aves	Non-Passer	Trogoni	*Trogon rufus*	53.00	77.50	Yarbrough 1971
Aves	Passeriformes	Passeri	*Abroscopus superciliaris*	6.71	26.68	Bushuev et al. (unpublished)
Aves	Passeriformes	Passeri	*Acanthagenys rufogularis*	43.40	114.00	Bech et al. 2016
Aves	Passeriformes	Passeri	*Acanthis flammea*	14.70	62.50	Reinertsen & Haftorn 1986
Aves	Passeriformes	Passeri	*Acanthiza chrysorrhoa*	8.30	40.20	Bech et al. 2016
Aves	Passeriformes	Passeri	*Acanthiza lineata*	7.30	28.80	Bech et al. 2016
Aves	Passeriformes	Passeri	*Acanthiza pusilla*	6.40	40.20	Bech et al. 2016
Aves	Passeriformes	Passeri	*Acanthiza uropygialis*	6.60	31.80	Bech et al. 2016
Aves	Passeriformes	Passeri	*Acanthorhynchus tenuirostris*	11.30	49.80	Bech et al. 2016
Aves	Passeriformes	Passeri	*Acridotheres cristatellus*	109.40	217.50	Gohnson, McTaggart 1975
Aves	Passeriformes	Passeri	*Acridotheres tristis*	102.30	159.00	Bech et al. 2016
Aves	Passeriformes	Passeri	*Acrocephalus arundinaceus*	21.90	46.00	Hails 1983
Aves	Passeriformes	Passeri	*Acrocephalus bistrigiceps*	7.01	28.67	Bushuev et al. (unpublished)
Aves	Passeriformes	Passeri	*Acrocephalus bistrigiceps*	7.90	23.00	Hails 1983
Aves	Passeriformes	Passeri	*Acrocephalus palustris*	10.80	36.50	Kendeigh et al. 1977
Aves	Passeriformes	Passeri	*Acrocephalus schoenobaenus*	11.50	39.00	Kendeigh et al. 1977
Aves	Passeriformes	Passeri	*Acrocephalus orientalis*	20.79	46.01	Bushuev et al. (unpublished)
Aves	Passeriformes	Passeri	*Aegithalos caudatus*	8.90	36.00	Gavrilov & Dolnik 1985
Aves	Passeriformes	Passeri	*Aegithina lafresnayei*	18.22	44.51	Bushuev et al. (unpublished)
Aves	Passeriformes	Passeri	*Aegithina tiphia*	12.05	29.09	Bushuev et al. (unpublished)
Aves	Passeriformes	Passeri	*Aethomyias papuensis*	10.40	33.00	McNab 2009
Aves	Passeriformes	Passeri	*Aethomyias perspicillatus*	8.80	39.00	McNab 2009
Aves	Passeriformes	Passeri	*Aethopyga christinae*	5.20	24.00	Prinzinger et al. 1989
Aves	Passeriformes	Passeri	*Aethopyga siparaja*	6.16	24.97	Bushuev et al. (unpublished)
Aves	Passeriformes	Passeri	*Aethopyga siparaja*	6.80	26.50	Prinzinger et al. 1989
Aves	Passeriformes	Passeri	*Agelaius phoeniceus*	43.10	87.00	Weathers 1981
Aves	Passeriformes	Passeri	*Akletos goeldii*	42.40	76.36	Londono et al. 2015

Aves	Passeriformes	Passeri	*Alaemon alaudipes*	37.70	77.00	Tieleman et al. 2002
Aves	Passeriformes	Passeri	*Alauda arvensis*	31.70	130.00	Tieleman et al. 2002
Aves	Passeriformes	Passeri	*Alcippe grotei*	15.40	67.55	Bushuev et al. (unpublished)
Aves	Passeriformes	Passeri	*Aleadryas rufinucha*	38.60	75.00	McNab, pers. obs.
Aves	Passeriformes	Passeri	*Alophoixus bres*	35.00	64.00	Hails 1983
Aves	Passeriformes	Passeri	*Alophoixus ochraceus*	34.18	68.44	Bushuev et al. (unpublished)
Aves	Passeriformes	Passeri	*Amadina erythrocephala*	21.70	38.00	McKechnie & Lovegrove 2003
Aves	Passeriformes	Passeri	*Amadina fasciata*	17.20	38.50	Marschall & Prinzinger 1991
Aves	Passeriformes	Passeri	*Amblycercus holosericeus*	50.40	103.64	Londono et al. 2015
Aves	Passeriformes	Passeri	*Ammodramus aurifrons*	16.40	47.27	Londono et al. 2015
Aves	Passeriformes	Passeri	*Ammodramus savannarum*	13.80	32.00	Yarbrough 1971
Aves	Passeriformes	Passeri	*Amphispiza bilineata*	11.60	35.50	Weathers 1981
Aves	Passeriformes	Passeri	*Anabacerthia striaticollis*	24.20	63.64	Londono et al. 2015
Aves	Passeriformes	Passeri	*Anabazenops dorsalis*	39.20	81.82	Londono et al. 2015
Aves	Passeriformes	Passeri	*Anairetes parulus*	6.60	27.27	Londono et al. 2015
Aves	Passeriformes	Passeri	*Ancistrops strigilatus*	36.00	40.00	Londono et al. 2015
Aves	Passeriformes	Passeri	*Anisognathus igniventris*	35.00	80.00	Londono et al. 2015
Aves	Passeriformes	Passeri	*Anthreptes orientalis*	11.80	27.50	Prinzinger et al. 1989
Aves	Passeriformes	Passeri	*Anthus campestris*	21.80	69.00	Kendeigh et al. 1977
Aves	Passeriformes	Passeri	*Anthus pratensis*	18.90	54.00	Kendeigh et al. 1977
Aves	Passeriformes	Passeri	*Anthus rufulus*	20.10	52.50	Bushuev et al. (unpublished)
Aves	Passeriformes	Passeri	*Anthus trivialis*	19.70	61.00	Kendeigh et al. 1977
Aves	Passeriformes	Passeri	*Arachnothera flavigaster*	36.30	54.00	Hails 1983
Aves	Passeriformes	Passeri	*Arachnothera longirostra*	11.19	36.72	Bushuev et al. (unpublished)
Aves	Passeriformes	Passeri	*Arachnothera longirostra*	13.00	34.00	Hails 1983
Aves	Passeriformes	Passeri	*Arremon castaneiceps*	35.70	96.36	Londono et al. 2015
Aves	Passeriformes	Passeri	*Arremon taciturnus*	27.70	76.36	Londono et al. 2015
Aves	Passeriformes	Passeri	*Arremon torquatus*	42.80	85.45	Londono et al. 2015
Aves	Passeriformes	Passeri	*Arremonops conirostris*	39.70	81.50	Wiersma et al. 2007
Aves	Passeriformes	Passeri	*Artamus maximus*	60.00	99.50	McNab, pers. obs.
Aves	Passeriformes	Passeri	*Arundinax aedon*	19.35	63.26	Bushuev et al. (unpublished)
Aves	Passeriformes	Passeri	*Asthenes helleri*	15.00	34.55	Londono et al., 2015
Aves	Passeriformes	Passeri	*Astrapia stephaniae*	148.20	290.00	McNab 2005
Aves	Passeriformes	Passeri	*Atlapetes melanolaemus*	25.50	61.82	Londono et al. 2015
Aves	Passeriformes	Passeri	*Attila spadiceus*	32.70	25.45	Londono et al. 2015
Aves	Passeriformes	Passeri	*Auriparus flaviceps*	6.80	29.50	Goldstein 1974
Aves	Passeriformes	Passeri	*Automolus melanopezus*	31.60	69.09	Londono et al. 2015
Aves	Passeriformes	Passeri	*Automolus ochrolaemus*	36.70	114.55	Londono et al. 2015
Aves	Passeriformes	Passeri	*Automolus rufipileatus*	36.20	83.64	Londono et al. 2015
Aves	Passeriformes	Passeri	*Automolus subulatus*	30.20	89.09	Londono et al. 2015
Aves	Passeriformes	Passeri	*Basileuterus tristriatus*	12.60	45.45	Londono et al. 2015
Aves	Passeriformes	Passeri	*Bombycilla garrulus*	72.50	171.50	Kendeigh et al. 1977
Aves	Passeriformes	Passeri	*Buthraupis montana*	89.70	185.45	Londono et al. 2015
Aves	Passeriformes	Passeri	*Cacicus chrysonotus*	94.00	154.55	Londono et al. 2015
Aves	Passeriformes	Passeri	*Calamospiza melanocorys*	33.00	79.00	Wunder 1979
Aves	Passeriformes	Passeri	*Calendulauda erythrochlamys*	27.30	75.00	Williams 1999
Aves	Passeriformes	Passeri	*Calliope calliope*	18.34	50.42	Bushuev et al. (unpublished)
Aves	Passeriformes	Passeri	*Camptostoma obsoletum*	12.50	47.50	Wiersma et al. 2007
Aves	Passeriformes	Passeri	*Cantorchilus leucotis*	18.00	38.00	Wiersma et al. 2007
Aves	Passeriformes	Passeri	*Cardinalis cardinalis*	41.00	90.50	Hinds & Calder 1973
Aves	Passeriformes	Passeri	*Cardinalis sinuatus*	32.00	70.50	Hinds & Calder 1973

Aves	Passeriformes	Passeri	*Carduelis carduelis*	16.50	62.50	Kendeigh et al. 1977
Aves	Passeriformes	Passeri	*Carpodacus erythrinus*	21.20	66.50	Kendeigh et al. 1977
Aves	Passeriformes	Passeri	*Catamblyrhynchus diadema*	17.00	45.45	Londono et al. 2015
Aves	Passeriformes	Passeri	*Catharus dryas*	35.90	83.64	Londono et al. 2015
Aves	Passeriformes	Passeri	*Catharus fuscescens*	28.00	90.50	Holmes, Sawyer 1975
Aves	Passeriformes	Passeri	*Catharus guttatus*	27.80	85.50	Holmes, Sawyer, 1975
Aves	Passeriformes	Passeri	*Catharus minimus*	24.80	82.50	Holmes, Sawyer 1975
Aves	Passeriformes	Passeri	*Catharus ustulatus*	27.90	84.00	Holmes, Sawyer 1975
Aves	Passeriformes	Passeri	*Ceratopipra chloromeros*	16.20	52.73	Londono et al. 2015
Aves	Passeriformes	Passeri	*Ceratopipra mentalis*	12.30	35.00	Bartholomew et al. 1983
Aves	Passeriformes	Passeri	*Cercomacroides tyrannina*	15.40	34.00	Wiersma et al. 2007
Aves	Passeriformes	Passeri	*Chalcomitra adelberti*	9.50	31.50	Prinzinger et al. 1989
Aves	Passeriformes	Passeri	*Chalcomitra amethystina*	10.00	31.50	Prinzinger et al. 1989
Aves	Passeriformes	Passeri	*Chalcomitra rubescens*	10.00	33.00	Seavy 2006
Aves	Passeriformes	Passeri	*Chalcomitra senegalensis*	13.70	43.00	Seavy 2006
Aves	Passeriformes	Passeri	*Chalcoparia singalensis*	7.34	26.22	Bushuev et al. (unpublished)
Aves	Passeriformes	Passeri	*Chiroxiphia boliviana*	17.00	54.55	Londono et al. 2015
Aves	Passeriformes	Passeri	*Chiroxiphia lanceolata*	18.40	61.00	Wiersma et al. 2007
Aves	Passeriformes	Passeri	*Chloris chloris*	28.20	85.50	Kendeigh et al. 1977
Aves	Passeriformes	Passeri	*Chlorochrysa calliparaea*	16.90	45.45	Londono et al., 2015
Aves	Passeriformes	Passeri	*Chlorodrepanis virens*	15.30	53.00	MacMillen 1974
Aves	Passeriformes	Passeri	*Chlorodrepanis virens*	10.70	37.50	MacMillen 1981
Aves	Passeriformes	Passeri	*Chloropsis aurifrons*	27.90	62.60	Bushuev et al. (unpublished)
Aves	Passeriformes	Passeri	*Chloropsis cochinchinensis*	21.20	47.58	Bushuev et al. (unpublished)
Aves	Passeriformes	Passeri	*Chloropsis sonnerati*	39.70	68.00	Hails 1983
Aves	Passeriformes	Passeri	*Chlorornis riefferii*	49.80	92.73	Londono et al. 2015
Aves	Passeriformes	Passeri	*Chlorospingus flavigularis*	26.50	60.00	Londono et al. 2015
Aves	Passeriformes	Passeri	*Chlorospingus parvirostris*	23.10	60.00	Londono et al., 2015
Aves	Passeriformes	Passeri	*Cicinnurus regius*	54.00	105.00	McNab 2005
Aves	Passeriformes	Passeri	*Cinclus mexicanus*	50.20	83.00	Murrish 1970
Aves	Passeriformes	Passeri	*Cinnycerthia fulva*	16.30	36.36	Londono et al. 2015
Aves	Passeriformes	Passeri	*Cinnyris bifasciatus*	6.20	16.50	Prinzinger et al. 1989
Aves	Passeriformes	Passeri	*Cinnyris chloropygius*	6.80	25.00	Seavy 2006
Aves	Passeriformes	Passeri	*Cinnyris cupreus*	9.00	29.50	Seavy 2006
Aves	Passeriformes	Passeri	*Cinnyris jugularis*	5.87	23.71	Bushuev et al. (unpublished)
Aves	Passeriformes	Passeri	*Cinnyris venustus*	7.10	25.00	Prinzinger et al. 1989
Aves	Passeriformes	Passeri	*Cissopis leverianus*	61.40	50.91	Londono et al. 2015
Aves	Passeriformes	Passeri	*Cnemophilus loriae*	78.10	119.00	McNab 2005
Aves	Passeriformes	Passeri	*Cnemophilus macgregorii*	89.00	134.00	McNab, pers. obs.
Aves	Passeriformes	Passeri	*Cnipodectes subbrunneus*	20.50	49.50	Wiersma et al. 2007
Aves	Passeriformes	Passeri	*Coccothraustes coccothraustes*	48.30	125.50	Kendeigh et al. 1977
Aves	Passeriformes	Passeri	*Coereba flaveola*	11.00	43.64	Londono et al. 2015
Aves	Passeriformes	Passeri	*Coereba flaveola*	10.00	38.50	Merola-Zwartjes 1998
Aves	Passeriformes	Passeri	*Coloeus monedula*	188.00	309.50	Daan et al. 1990
Aves	Passeriformes	Passeri	*Conirostrum sitticolor*	11.00	32.73	Londono et al. 2015
Aves	Passeriformes	Passeri	*Conopophaga ardesiaca*	27.50	70.91	Londono et al. 2015
Aves	Passeriformes	Passeri	*Conopophaga peruviana*	24.50	61.82	Londono et al. 2015
Aves	Passeriformes	Passeri	*Contopus virens*	13.90	46.50	Yarbrough 1971
Aves	Passeriformes	Passeri	*Copsychus malabaricus*	27.24	57.65	Bushuev et al. (unpublished)
Aves	Passeriformes	Passeri	*Copsychus saularis*	35.61	64.85	Bushuev et al. (unpublished)
Aves	Passeriformes	Passeri	*Copsychus saularis*	33.50	41.50	Hails 1983

Aves	Passeriformes	Passeri	*Corvus brachyrhynchos*	384.80	591.50	Wunder, Trebella 1976
Aves	Passeriformes	Passeri	*Corvus caurinus*	282.00	638.50	Irving et al. 1955
Aves	Passeriformes	Passeri	*Corvus corax*	1203.00	990.00	Kendeigh et al. 1977
Aves	Passeriformes	Passeri	*Corvus corone*	518.00	596.50	Kendeigh et al. 1977
Aves	Passeriformes	Passeri	*Corvus fuscicapillus*	390.00	471.00	Kendeigh et al. 1977
Aves	Passeriformes	Passeri	*Corvus ruficollis*	660.00	611.50	Kendeigh et al. 1977
Aves	Passeriformes	Passeri	*Corydon sumatranus*	115.33	107.75	Bushuev et al. (unpublished)
Aves	Passeriformes	Passeri	*Corythopis torquatus*	18.70	50.91	Londono et al. 2015
Aves	Passeriformes	Passeri	*Cracticus torquatus*	93.70	127.80	Bech et al. 2016
Aves	Passeriformes	Passeri	*Cranioleuca marcapatae*	19.00	50.91	Londono et al. 2015
Aves	Passeriformes	Passeri	*Crypsirina temia*	47.74	82.80	Bushuev et al. (unpublished)
Aves	Passeriformes	Passeri	*Cryptopipo holochlora*	16.00	38.18	Londono et al., 2015
Aves	Passeriformes	Passeri	*Cyanerpes cyaneus*	13.50	49.00	Wiersma et al. 2007
Aves	Passeriformes	Passeri	*Cyanistes caeruleus*	9.60	30.00	Lindstrom & Kvist 1995
Aves	Passeriformes	Passeri	*Cyanocitta cristata*	80.80	150.00	Misch 1960
Aves	Passeriformes	Passeri	*Cyanocompsa cyanoides*	25.00	69.09	Londono et al. 2015
Aves	Passeriformes	Passeri	*Cyanocompsa cyanoides*	27.90	67.50	Wiersma et al. 2007
Aves	Passeriformes	Passeri	*Cyanolyca viridicyanus*	82.60	194.55	Londono et al. 2015
Aves	Passeriformes	Passeri	*Cyanomitra cyanolaema*	15.80	49.00	Seavy 2006
Aves	Passeriformes	Passeri	*Cyanomitra obscura*	11.20	36.00	Seavy 2006
Aves	Passeriformes	Passeri	*Cyanomitra veroxii*	8.40	24.00	Prinzinger et al. 1989
Aves	Passeriformes	Passeri	*Cyanomitra verticalis*	14.10	45.00	Seavy 2006
Aves	Passeriformes	Passeri	*Cymbirhynchus macrorhynchos*	52.64	86.49	Bushuev et al. (unpublished)
Aves	Passeriformes	Passeri	*Cyornis hainanus*	13.32	38.55	Bushuev et al. (unpublished)
Aves	Passeriformes	Passeri	*Cyornis sumatrensis*	12.49	38.87	Bushuev et al. (unpublished)
Aves	Passeriformes	Passeri	*Cyphorhinus thoracicus*	34.40	76.36	Londono et al. 2015
Aves	Passeriformes	Passeri	*Delichon urbicum*	18.00	23.00	Prinzinger & Hanssler 1980
Aves	Passeriformes	Passeri	*Dendrocincla fuliginosa*	33.10	67.27	Londono et al., 2015
Aves	Passeriformes	Passeri	*Dendrocincla fuliginosa*	39.00	88.50	Wiersma et al. 2007
Aves	Passeriformes	Passeri	*Dendrocincla merula*	52.80	112.73	Londono et al. 2015
Aves	Passeriformes	Passeri	*Dendrocincla tyrannina*	58.30	96.36	Londono et al. 2015
Aves	Passeriformes	Passeri	*Dendrocolaptes picumnus*	80.20	130.91	Londono et al. 2015
Aves	Passeriformes	Passeri	*Devioeca papuana*	12.50	55.50	McNab, pers. obs.
Aves	Passeriformes	Passeri	*Dicaeum cruentatum*	5.08	15.56	Bushuev et al. (unpublished)
Aves	Passeriformes	Passeri	*Dicrurus aeneus*	29.84	73.62	Bushuev et al. (unpublished)
Aves	Passeriformes	Passeri	*Dicrurus hottentottus*	76.20	128.14	Bushuev et al. (unpublished)
Aves	Passeriformes	Passeri	*Dicrurus leucophaeus*	41.72	80.32	Bushuev et al. (unpublished)
Aves	Passeriformes	Passeri	*Dicrurus macrocercus*	63.50	94.63	Bushuev et al. (unpublished)
Aves	Passeriformes	Passeri	*Dicrurus paradiseus*	71.60	112.15	Bushuev et al. (unpublished)
Aves	Passeriformes	Passeri	*Diglossa brunneiventris*	10.60	43.64	Londono et al. 2015
Aves	Passeriformes	Passeri	*Diglossa cyanea*	19.10	58.18	Londono et al., 2015
Aves	Passeriformes	Passeri	*Diglossa mystacalis*	15.40	50.91	Londono et al. 2015
Aves	Passeriformes	Passeri	*Diphyllodes magnificus*	82.30	142.00	McNab 2005
Aves	Passeriformes	Passeri	*Drepanis coccinea*	16.90	62.50	MacMi11en, Carpenter 1977
Aves	Passeriformes	Passeri	*Drepanorhynchus reichenowi*	6.70	25.50	Seavy 2006
Aves	Passeriformes	Passeri	*Drymophila caudata*	12.20	25.45	Londono et al. 2015
Aves	Passeriformes	Passeri	*Elaenia albiceps*	16.80	34.55	Londono et al. 2015
Aves	Passeriformes	Passeri	*Elaenia gigas*	30.70	74.55	Londono et al. 2015
Aves	Passeriformes	Passeri	*Elaenia obscura*	13.90	25.45	Londono et al. 2015
Aves	Passeriformes	Passeri	*Elaenia pallatangae*	15.70	41.82	Londono et al. 2015
Aves	Passeriformes	Passeri	*Emberiza citrinella*	26.80	78.50	Gavrilov & Dolnik 1985

Aves	Passeriformes	Passeri	*Emberiza hortulana*	24.30	75.00	Gavrilov & Dolnik 1985
Aves	Passeriformes	Passeri	*Emberiza schoeniclus*	17.60	54.00	Kendeigh et al. 1977
Aves	Passeriformes	Passeri	*Empidonax virescens*	12.30	32.00	Yarbrough 1971
Aves	Passeriformes	Passeri	*Entomodestes leucotis*	60.70	47.27	Londono et al. 2015
Aves	Passeriformes	Passeri	*Eopsaltria australis*	19.20	63.00	Bech et al. 2016
Aves	Passeriformes	Passeri	*Epimachus meyeri*	202.70	331.50	McNab 2005
Aves	Passeriformes	Passeri	*Epinecrophylla leucophthalma*	9.40	34.55	Londono et al. 2015
Aves	Passeriformes	Passeri	*Eremalauda dunni*	20.60	50.00	Tieleman et al. 2002
Aves	Passeriformes	Passeri	*Eremophila alpestris*	26.00	59.50	Trost 1972
Aves	Passeriformes	Passeri	*Erithacus rubecula*	17.60	50.50	Kendeigh et al. 1977
Aves	Passeriformes	Passeri	*Erythrura gouldiae*	15.50	39.00	Marschall & Prinzinger 1991
Aves	Passeriformes	Passeri	*Erythrura trichroa*	15.10	43.00	McNab, pers. obs.
Aves	Passeriformes	Passeri	*Estrilda melpoda*	7.50	23.50	Marschall & Prinzinger 1991
Aves	Passeriformes	Passeri	*Estrilda troglodytes*	7.50	27.00	Kendeigh et al. 1977
Aves	Passeriformes	Passeri	*Eucometis penicillata*	30.70	71.00	Wiersma et al. 2007
Aves	Passeriformes	Passeri	*Eulacestoma nigropectus*	19.90	62.00	McNab, pers. obs.
Aves	Passeriformes	Passeri	*Euodice malabarica*	10.60	36.50	Willoughby 1969
Aves	Passeriformes	Passeri	*Euphonia laniirostris*	13.50	53.00	Wiersma et al. 2007
Aves	Passeriformes	Passeri	*Euphonia xanthogaster*	12.90	40.00	Londono et al. 2015
Aves	Passeriformes	Passeri	*Eurillas curvirostris*	23.00	53.00	Seavy & McNab 2007
Aves	Passeriformes	Passeri	*Eurillas latirostris*	26.60	65.00	Seavy & McNab 2007
Aves	Passeriformes	Passeri	*Eurillas virens*	24.20	59.50	Seavy & McNab 2007
Aves	Passeriformes	Passeri	*Ficedula albicilla*	9.53	42.36	Bushuev et al. (unpublished)
Aves	Passeriformes	Passeri	*Ficedula hypoleuca*	11.70	42.00	Kendeigh et al. 1977
Aves	Passeriformes	Passeri	*Ficedula mugimaki*	12.20	63.98	Bushuev et al. (unpublished)
Aves	Passeriformes	Passeri	*Formicarius analis*	54.90	112.73	Londono et al. 2015
Aves	Passeriformes	Passeri	*Formicarius rufipectus*	70.30	132.73	Londono et al. 2015
Aves	Passeriformes	Passeri	*Fringilla coelebs*	21.00	67.00	Gavrilov & Dolnik 1985
Aves	Passeriformes	Passeri	*Fringilla montifringilla*	21.00	69.00	Kendeigh et al. 1977
Aves	Passeriformes	Passeri	*Garrulax leucolophus*	106.83	148.91	Bushuev et al. (unpublished)
Aves	Passeriformes	Passeri	*Garrulax monileger*	83.50	131.37	Bushuev et al. (unpublished)
Aves	Passeriformes	Passeri	*Garrulus glandarius*	153.00	249.50	Kendeigh et al. 1977
Aves	Passeriformes	Passeri	*Gavicalis virescens*	20.70	63.60	Bech et al. 2016
Aves	Passeriformes	Passeri	*Gavicalis virescens*	25.00	53.50	Collins et al. 1980
Aves	Passeriformes	Passeri	*Gavicalis virescens*	25.00	53.50	Collins et al. 1980
Aves	Passeriformes	Passeri	*Geokichla citrina*	56.60	95.10	Bushuev et al. (unpublished)
Aves	Passeriformes	Passeri	*Gliciphila melanops*	18.80	52.50	Vitali et al. 1999
Aves	Passeriformes	Passeri	*Glyphorynchus spirurus*	15.50	45.45	Londono et al. 2015
Aves	Passeriformes	Passeri	*Glyphorynchus spirurus*	13.50	36.00	Wiersma et al. 2007
Aves	Passeriformes	Passeri	*Grallaria guatimalensis*	87.00	112.73	Londono et al. 2015
Aves	Passeriformes	Passeri	*Grallaria rufula*	33.80	94.55	Londono et al. 2015
Aves	Passeriformes	Passeri	*Gymnopithys bicolor*	27.70	60.00	Wiesma et al. 2007
Aves	Passeriformes	Passeri	*Habia fuscicauda*	40.00	64.00	Wiersma et al. 2007
Aves	Passeriformes	Passeri	*Habia rubica*	36.70	105.45	Londono et al., 2015
Aves	Passeriformes	Passeri	*Haemorhous cassinii*	27.40	61.00	Weathers 1981
Aves	Passeriformes	Passeri	*Haemorhous mexicanus*	20.40	56.00	Weathers 1981
Aves	Passeriformes	Passeri	*Haplospiza rustica*	18.00	25.45	Londono et al. 2015
Aves	Passeriformes	Passeri	*Hedydipna collaris*	8.30	30.50	Prinzinger et al. 1989
Aves	Passeriformes	Passeri	*Hemipus picatus*	9.70	23.96	Bushuev et al. (unpublished)
Aves	Passeriformes	Passeri	*Hemispingus atropileus*	20.40	69.09	Londono et al. 2015
Aves	Passeriformes	Passeri	*Hemispingus melanotis*	17.30	58.18	Londono et al. 2015

Aves	Passeriformes	Passeri	*Hemispingus superciliaris*	14.60	58.18	Londono et al. 2015
Aves	Passeriformes	Passeri	*Hemispingus xanthophthalmus*	13.00	34.55	Londono et al. 2015
Aves	Passeriformes	Passeri	*Hemitriccus granadensis*	8.20	25.45	Londono et al. 2015
Aves	Passeriformes	Passeri	*Henicorhina leucophrys*	15.30	56.36	Londono et al., 2015
Aves	Passeriformes	Passeri	*Hesperiphona vespertina*	54.50	135.50	West & Hart 1966
Aves	Passeriformes	Passeri	*Himatione sanguinea*	13.50	54.00	Weathers et al. 1983
Aves	Passeriformes	Passeri	*Hippolais icterina*	12.50	84.50	Kendeigh et al. 1977
Aves	Passeriformes	Passeri	*Hirundo rustica*	13.79	45.07	Bushuev et al. (unpublished)
Aves	Passeriformes	Passeri	*Hirundo rustica*	18.40	54.00	Gavrilov & Dolnik 1985
Aves	Passeriformes	Passeri	*Hirundo tahitica*	14.10	32.00	Bryant et al. 1984
Aves	Passeriformes	Passeri	*Hydrornis elliotii*	86.95	98.18	Bushuev et al. (unpublished)
Aves	Passeriformes	Passeri	*Hydrornis soror*	96.27	102.59	Bushuev et al. (unpublished)
Aves	Passeriformes	Passeri	*Hylocichla mustelina*	48.30	122.00	Holmes, Sawyer 1975
Aves	Passeriformes	Passeri	*Hylophylax naevioides*	16.10	43.00	Wiersma et al. 2007
Aves	Passeriformes	Passeri	*Hylophylax naevius*	12.80	63.64	Londono et al. 2015
Aves	Passeriformes	Passeri	*Hypocnemis subflava*	15.60	43.64	Londono et al. 2015
Aves	Passeriformes	Passeri	*Hypothymis azurea*	10.53	38.40	Bushuev et al. (unpublished)
Aves	Passeriformes	Passeri	*Hypothymis azurea*	10.80	32.00	Hails 1983
Aves	Passeriformes	Passeri	*Icterus bullockii*	34.00	90.50	Rising 1969
Aves	Passeriformes	Passeri	*Icterus galbula*	34.00	90.50	Rising 1969
Aves	Passeriformes	Passeri	*Ifrita kowaldi*	28.90	56.50	McNab 2009.
Aves	Passeriformes	Passeri	*Iole propinqua*	20.42	49.38	Bushuev et al. (unpublished)
Aves	Passeriformes	Passeri	*Irena puella*	58.12	92.74	Bushuev et al. (unpublished)
Aves	Passeriformes	Passeri	*Iridosornis analis*	27.10	85.45	Londono et al. 2015
Aves	Passeriformes	Passeri	*Iridosornis jelskii*	20.60	69.09	Londono et al. 2015
Aves	Passeriformes	Passeri	*Isleria hauxwelli*	10.80	38.18	Londono et al. 2015
Aves	Passeriformes	Passeri	*Junco hyemalis*	18.00	53.00	Lasiewski, Dawson 1967
Aves	Passeriformes	Passeri	*Knipolegus aterrimus*	34.00	56.36	Londono et al. 2015
Aves	Passeriformes	Passeri	*Kurochkinegramma hypogrammicum*	10.79	31.32	Bushuev et al. (unpublished)
Aves	Passeriformes	Passeri	*Lanio versicolor*	20.40	60.00	Londono et al. 2015
Aves	Passeriformes	Passeri	*Lanius collurio*	27.00	69.00	Kendeigh et al. 1977
Aves	Passeriformes	Passeri	*Lanius cristatus*	29.10	74.12	Bushuev et al. (unpublished)
Aves	Passeriformes	Passeri	*Lanius cristatus*	26.90	45.50	Hails 1983
Aves	Passeriformes	Passeri	*Lanius excubitor*	72.40	184.00	Kendeigh et al. 1977
Aves	Passeriformes	Passeri	*Larvivora cyane*	13.35	38.24	Bushuev et al. (unpublished)
Aves	Passeriformes	Passeri	*Larvivora cyane*	13.40	47.50	Hails 1983
Aves	Passeriformes	Passeri	*Lathrotriccus euleri*	9.30	38.18	Londono et al. 2015
Aves	Passeriformes	Passeri	*Legatus leucophaius*	26.70	29.09	Londono et al. 2015
Aves	Passeriformes	Passeri	*Leistes militaris*	38.20	79.50	Wiersma et al. 2007
Aves	Passeriformes	Passeri	*Lepidocolaptes lacrymiger*	32.60	40.00	Londono et al. 2015
Aves	Passeriformes	Passeri	*Lepidothrix coeruleocapilla*	9.60	43.64	Londono et al., 2015
Aves	Passeriformes	Passeri	*Lepidothrix coronata*	9.20	27.00	Wiersma et al. 2007
Aves	Passeriformes	Passeri	*Lepidothrix coronata*	10.60	36.36	Londono et al. 2015
Aves	Passeriformes	Passeri	*Leptocoma minima*	5.50	21.00	Seavy 2006
Aves	Passeriformes	Passeri	*Leptocoma sperata*	5.10	24.28	Bushuev et al. (unpublished)
Aves	Passeriformes	Passeri	*Leptopogon amaurocephalus*	14.10	40.00	Londono et al. 2015
Aves	Passeriformes	Passeri	*Leptopogon superciliaris*	12.90	45.45	Londono et al. 2015
Aves	Passeriformes	Passeri	*Lichmera indistincta*	10.10	38.00	Vitali et al. 1999
Aves	Passeriformes	Passeri	*Linaria cannabina*	16.90	61.00	Kendeigh et al. 1977
Aves	Passeriformes	Passeri	*Lipaugus vociferans*	86.60	138.18	Londono et al. 2015

Aves	Passeriformes	Passeri	*Lochmias nematura*	30.50	61.82	Londono et al. 2015
Aves	Passeriformes	Passeri	*Locustella lanceolata*	9.45	32.72	Bushuev et al. (unpublished)
Aves	Passeriformes	Passeri	*Lonchura cucullata*	10.60	19.50	Lovegrove & Smith 2003
Aves	Passeriformes	Passeri	*Lonchura fuscans*	9.50	18.00	Weathers 1977
Aves	Passeriformes	Passeri	*Lonchura maja*	12.80	27.50	Hails 1983
Aves	Passeriformes	Passeri	*Lonchura malacca*	11.80	25.50	Hails 1983
Aves	Passeriformes	Passeri	*Lonchura oryzivora*	25.40	55.50	Marschell & Prinzinger 1991
Aves	Passeriformes	Passeri	*Lonchura punctulata*	12.23	37.60	Bushuev et al. (unpublished)
Aves	Passeriformes	Passeri	*Lonchura striata*	9.74	33.20	Bushuev et al. (unpublished)
Aves	Passeriformes	Passeri	*Lonchura striata*	10.30	38.50	Kendeigh et al. 1977
Aves	Passeriformes	Passeri	*Lophorina superba*	74.60	130.00	McNab 2005
Aves	Passeriformes	Passeri	*Lophotriccus pileatus*	8.20	34.55	Londono et al. 2015
Aves	Passeriformes	Passeri	*Loxia curvirostra*	39.40	108.00	Kendeigh et al. 1977
Aves	Passeriformes	Passeri	*Loxia leucoptera*	29.80	83.50	Dawson & Tordoff 1964
Aves	Passeriformes	Passeri	*Loxia pytyopsittacus*	53.70	143.50	Kendeigh et al. 1977
Aves	Passeriformes	Passeri	*Loxioides bailleui*	34.80	80.50	Weathers & van Riper 1982
Aves	Passeriformes	Passeri	*Loxioides bailleui*	36.00	80.50	Weathers, van Riper 1982
Aves	Passeriformes	Passeri	*Lullula arborea*	25.50	103.00	Tieleman et al. 2002
Aves	Passeriformes	Passeri	*Luscinia svecica*	20.80	64.50	Kendeigh et al. 1977
Aves	Passeriformes	Passeri	*Machaerirhynchus nigripectus*	9.70	41.50	McNab, pers. obs.
Aves	Passeriformes	Passeri	*Machaeropterus pyrocephalus*	10.00	43.64	Londono et al. 2015
Aves	Passeriformes	Passeri	*Macronus gularis*	11.27	31.86	Bushuev et al. (unpublished)
Aves	Passeriformes	Passeri	*Macronus kelleyi*	9.47	30.48	Bushuev et al. (unpublished)
Aves	Passeriformes	Passeri	*Magumma parva*	7.90	25.50	MacMillen 1981
Aves	Passeriformes	Passeri	*Malacocincla abbotti*	25.63	53.55	Bushuev et al. (unpublished)
Aves	Passeriformes	Passeri	*Malacopteron cinereum*	14.33	36.87	Bushuev et al. (unpublished)
Aves	Passeriformes	Passeri	*Malacopteron cinereum*	15.80	38.00	Hails1983
Aves	Passeriformes	Passeri	*Malurus cyaneus*	8.70	31.80	Bech et al. 2016
Aves	Passeriformes	Passeri	*Malurus lamberti*	7.80	33.00	Bech et al., 2016
Aves	Passeriformes	Passeri	*Manacus vitellinus*	15.50	42.00	Bartholomew et al. 1983
Aves	Passeriformes	Passeri	*Manorina flavigula*	41.10	109.80	Bech et al. 2016
Aves	Passeriformes	Passeri	*Manucodia chalybatus*	177.20	247.00	McNab 2005
Aves	Passeriformes	Passeri	*Margarornis squamiger*	16.70	49.09	Londono et al. 2015
Aves	Passeriformes	Passeri	*Mecocerculus leucophrys*	13.90	27.27	Londono et al. 2015
Aves	Passeriformes	Passeri	*Mecocerculus stictopterus*	10.90	40.00	Londono et al. 2015
Aves	Passeriformes	Passeri	*Melanocharis versteri*	13.60	44.00	McNab 2009.
Aves	Passeriformes	Passeri	*Melanorectes nigrescens*	70.50	113.50	McNab 2009.
Aves	Passeriformes	Passeri	*Melidectes rufocrissalis*	62.80	140.00	McNab 2009.
Aves	Passeriformes	Passeri	*Meliphaga lewinii*	36.20	96.00	Bech et al. 2016
Aves	Passeriformes	Passeri	*Melipotes fumigatus*	57.10	122.00	McNab 2009.
Aves	Passeriformes	Passeri	*Melithreptus lunatus*	14.30	45.00	Vitali et al. 1999
Aves	Passeriformes	Passeri	*Melospiza georgiana*	14.90	38.00	Yarbrough 1971
Aves	Passeriformes	Passeri	*Melospiza melodia*	19.10	45.00	Yarbrough 1971
Aves	Passeriformes	Passeri	*Melozone aberti*	46.60	131.00	Dawson 1954
Aves	Passeriformes	Passeri	*Melozone fusca*	43.70	119.50	Dawson 1954
Aves	Passeriformes	Passeri	*Microcerculus marginatus*	19.40	60.00	Londono et al. 2015
Aves	Passeriformes	Passeri	*Mimus gilvus*	68.90	133.00	Wiersma et al. 2007
Aves	Passeriformes	Passeri	*Mimus polyglottos*	44.20	122.00	Wiersma et al. 2007
Aves	Passeriformes	Passeri	*Mionectes macconnelli*	11.40	29.09	Londono et al. 2015
Aves	Passeriformes	Passeri	*Mionectes oleagineus*	11.40	32.73	Londono et al. 2015
Aves	Passeriformes	Passeri	*Mionectes oleagineus*	10.30	29.50	Wiersma et al. 2007

Aves	Passeriformes	Passeri	*Mionectes olivaceus*	15.70	50.91	Londono et al. 2015
Aves	Passeriformes	Passeri	*Mionectes striaticollis*	14.70	49.09	Londono et al. 2015
Aves	Passeriformes	Passeri	*Molothrus ater*	42.50	90.50	Hinds, Calder 1973
Aves	Passeriformes	Passeri	*Monticola gularis*	27.00	49.20	Bushuev et al. (unpublished)
Aves	Passeriformes	Passeri	*Motacilla alba*	18.00	54.00	Gavrilov & Dolnik 1985
Aves	Passeriformes	Passeri	*Motacilla flava*	14.70	46.50	Kendeigh et al. 1977
Aves	Passeriformes	Passeri	*Muscicapa dauurica*	9.23	34.96	Bushuev et al. (unpublished)
Aves	Passeriformes	Passeri	*Muscicapa striata*	14.40	44.50	Kendeigh et al. 1977
Aves	Passeriformes	Passeri	*Muscisaxicola fluviatilis*	13.60	47.27	Londono et al. 2015
Aves	Passeriformes	Passeri	*Myadestes ralloides*	28.80	70.91	Londono et al. 2015
Aves	Passeriformes	Passeri	*Myiarchus crinitus*	33.90	69.00	Yarbrough 1971
Aves	Passeriformes	Passeri	*Myiobius villosus*	14.70	54.55	Londono et al. 2015
Aves	Passeriformes	Passeri	*Myioborus melanocephalus*	11.70	38.18	Londono et al. 2015
Aves	Passeriformes	Passeri	*Myioborus miniatus*	9.80	36.36	Londono et al. 2015
Aves	Passeriformes	Passeri	*Myiodynastes maculatus*	41.00	106.00	Wiersma et al. 2007
Aves	Passeriformes	Passeri	*Myiotheretes fuscorufus*	29.20	94.55	Londono et al. 2015
Aves	Passeriformes	Passeri	*Myiothlypis bivittata*	15.50	54.55	Londono et al. 2015
Aves	Passeriformes	Passeri	*Myiothlypis chrysogaster*	14.40	50.91	Londono et al. 2015
Aves	Passeriformes	Passeri	*Myiothlypis coronata*	17.40	60.00	Londono et al. 2015
Aves	Passeriformes	Passeri	*Myiothlypis fulvicauda*	12.80	63.64	Londono et al. 2015
Aves	Passeriformes	Passeri	*Myiothlypis luteoviridis*	14.70	43.64	Londono et al. 2015
Aves	Passeriformes	Passeri	*Myiothlypis signata*	14.30	40.00	Londono et al., 2015
Aves	Passeriformes	Passeri	*Myiozetetes similis*	27.10	52.73	Londono et al. 2015
Aves	Passeriformes	Passeri	*Myrmeciza longipes*	27.40	58.50	Wiesma et al. 2007
Aves	Passeriformes	Passeri	*Myrmelastes leucostigma*	17.80	32.73	Londono et al., 2015
Aves	Passeriformes	Passeri	*Myrmoborus leucophrys*	19.80	60.00	Londono et al. 2015
Aves	Passeriformes	Passeri	*Myrmoborus lophotes*	31.60	60.00	Londono et al. 2015
Aves	Passeriformes	Passeri	*Myrmoborus myotherinus*	21.20	50.91	Londono et al. 2015
Aves	Passeriformes	Passeri	*Myrmotherula axillaris*	8.90	34.55	Londono et al., 2015
Aves	Passeriformes	Passeri	*Myrmotherula axillaris*	9.60	39.00	Wiersma et al. 2007
Aves	Passeriformes	Passeri	*Myrmotherula longipennis*	9.60	40.00	Londono et al. 2015
Aves	Passeriformes	Passeri	*Myrmotherula menetriesii*	9.30	50.91	Londono et al. 2015
Aves	Passeriformes	Passeri	*Myrmotherula schisticolor*	8.30	45.45	Londono et al. 2015
Aves	Passeriformes	Passeri	*Nectarinia kilimensis*	16.20	47.00	Seavy 2006
Aves	Passeriformes	Passeri	*Nectarinia tacazze*	13.50	44.00	Prinzinger et al. 1989
Aves	Passeriformes	Passeri	*Neochmia temporalis*	9.60	31.80	Bech et al., 2016
Aves	Passeriformes	Passeri	*Neosericornis citreogularis*	16.90	65.40	Bech et al. 2016
Aves	Passeriformes	Passeri	*Nucifraga caryocatactes*	147.00	242.50	Kendeigh et al. 1977
Aves	Passeriformes	Passeri	*Ochthoeca rufipectoralis*	10.90	36.36	Londono et al. 2015
Aves	Passeriformes	Passeri	*Oneillornis salvini*	27.10	58.18	Londono et al. 2015
Aves	Passeriformes	Passeri	*Onychorhynchus coronatus*	14.00	54.55	Londono et al. 2015
Aves	Passeriformes	Passeri	*Oreocharis arfaki*	22.30	58.00	McNab, pers. obs.
Aves	Passeriformes	Passeri	*Oreostruthus fuliginosus*	16.00	29.50	McNab, pers. obs.
Aves	Passeriformes	Passeri	*Origma robusta*	17.30	45.50	McNab 2009
Aves	Passeriformes	Passeri	*Oriolus oriolus*	64.90	117.00	Kendeigh et al. 1977
Aves	Passeriformes	Passeri	*Oriolus xanthornus*	59.90	112.02	Bushuev et al. (unpublished)
Aves	Passeriformes	Passeri	*Orthotomus atrogularis*	6.73	27.35	Bushuev et al. (unpublished)
Aves	Passeriformes	Passeri	*Oryzoborus angolensis*	12.30	36.00	Wiersma et al. 2007
Aves	Passeriformes	Passeri	*Pachycephala pectoralis*	24.00	64.80	Bech et al. 2016
Aves	Passeriformes	Passeri	*Pachycephala schlegelii*	22.60	50.00	McNab, pers. obs.
Aves	Passeriformes	Passeri	*Pachycephala soror*	23.30	65.50	McNab, pers. obs.

Aves	Passeriformes	Passeri	*Paradisaea raggiana*	215.70	337.50	McNab 2005
Aves	Passeriformes	Passeri	*Paradisaea rudolphi*	156.10	274.00	McNab 2005
Aves	Passeriformes	Passeri	*Paramythia montium*	40.30	75.00	McNab, pers. obs.
Aves	Passeriformes	Passeri	*Pardalotus punctatus*	7.80	39.60	Bech et al. 2016
Aves	Passeriformes	Passeri	*Parkesia noveboracensis*	18.70	50.50	Yarbrough 1971
Aves	Passeriformes	Passeri	*Parotia lawesii*	144.90	206.50	McNab 2005
Aves	Passeriformes	Passeri	*Parotia wahnesi*	164.20	232.00	McNab 2005
Aves	Passeriformes	Passeri	*Parus major*	16.50	63.00	Reinertsen & Haftorn 1986
Aves	Passeriformes	Passeri	*Passer domesticus*	23.00	56.40	Bech et al. 2016
Aves	Passeriformes	Passeri	*Passer domesticus*	23.00	66.00	Kendeigh et al. 1977
Aves	Passeriformes	Passeri	*Passer montanus*	22.30	73.00	Kendeigh et al. 1977
Aves	Passeriformes	Passeri	*Passerculus sandwichensis*	15.90	40.00	Williams & Hansell 1981
Aves	Passeriformes	Passeri	*Passerella iliaca*	31.70	98.50	Lasiewski, Dawson 1967
Aves	Passeriformes	Passeri	*Pellorneum ruficeps*	21.85	53.32	Bushuev et al. (unpublished)
Aves	Passeriformes	Passeri	*Pellorneum tickelli*	15.80	39.29	Bushuev et al. (unpublished)
Aves	Passeriformes	Passeri	*Peneothello cyanus*	23.80	63.00	McNab, pers. obs.
Aves	Passeriformes	Passeri	*Peneothello sigillata*	21.60	56.00	McNab, pers. obs.
Aves	Passeriformes	Passeri	*Periparus ater*	10.80	42.50	Kendeigh et al. 1977
Aves	Passeriformes	Passeri	*Perisoreus canadensis*	71.20	122.00	Veghte 1964
Aves	Passeriformes	Passeri	*Pheugopedius fasciatoventris*	27.20	56.00	Wiersma et al. 2007
Aves	Passeriformes	Passeri	*Philemon buceroides*	140.50	253.00	Bonaccorso & McNab, 2003
Aves	Passeriformes	Passeri	*Philydor erythrocercum*	38.20	76.36	Londono et al. 2015
Aves	Passeriformes	Passeri	*Phlegopsis nigromaculata*	47.10	87.27	Londono et al. 2015
Aves	Passeriformes	Passeri	*Phoenicurus ochruros*	13.80	43.50	Kendeigh et al. 1977
Aves	Passeriformes	Passeri	*Phoenicurus phoenicurus*	13.00	42.00	Kendeigh et al. 1977
Aves	Passeriformes	Passeri	*Phonygammus keraudrenii*	170.70	215.50	McNab 2005
Aves	Passeriformes	Passeri	*Phyllastrephus hypochloris*	18.90	47.50	Seavy & McNab 2007
Aves	Passeriformes	Passeri	*Phylloscopus collybita*	8.20	29.50	Kendeigh et al. 1977
Aves	Passeriformes	Passeri	*Phylloscopus inornatus*	7.70	53.92	Bushuev et al. (unpublished)
Aves	Passeriformes	Passeri	*Phylloscopus plumbeitarsus*	5.95	21.08	Bushuev et al. (unpublished)
Aves	Passeriformes	Passeri	*Phylloscopus schwarzi*	7.80	28.17	Bushuev et al. (unpublished)
Aves	Passeriformes	Passeri	*Phylloscopus sibilatrix*	9.20	31.50	Kendeigh et al. 1977
Aves	Passeriformes	Passeri	*Phylloscopus tenellipes*	7.00	28.94	Bushuev et al. (unpublished)
Aves	Passeriformes	Passeri	*Phylloscopus trochilus*	10.70	37.50	Kendeigh et al. 1977
Aves	Passeriformes	Passeri	*Phytotoma rara*	41.60	103.00	Rezende et al. 2001
Aves	Passeriformes	Passeri	*Pica nuttalli*	151.90	264.00	Hayworth & Weathers 1984
Aves	Passeriformes	Passeri	*Pica pica*	158.90	215.50	Hayworth & Weathers 1984
Aves	Passeriformes	Passeri	*Pinicola enucleator*	78.40	195.50	Gavrilov & Dolnik 1985
Aves	Passeriformes	Passeri	*Pipra fasciicauda*	16.40	47.27	Londono et al. 2015
Aves	Passeriformes	Passeri	*Pipraeidea melanonota*	20.30	69.09	Londono et al. 2015
Aves	Passeriformes	Passeri	*Pipreola arcuata*	93.00	109.09	Londono et al. 2015
Aves	Passeriformes	Passeri	*Pipreola intermedia*	49.60	110.91	Londono et al., 2015
Aves	Passeriformes	Passeri	*Pitta moluccensis*	74.38	96.62	Bushuev et al. (unpublished)
Aves	Passeriformes	Passeri	*Pitta versicolor*	83.10	102.00	McNab 2009
Aves	Passeriformes	Passeri	*Platyrinchus coronatus*	10.80	29.09	Londono et al. 2015
Aves	Passeriformes	Passeri	*Platyrinchus platyrhynchos*	14.00	54.55	Londono et al. 2015
Aves	Passeriformes	Passeri	*Plectrophenax nivalis*	41.80	99.50	Scholander et al. 1950
Aves	Passeriformes	Passeri	*Plesiodryas albonotata*	36.40	81.00	McNab 2009.
Aves	Passeriformes	Passeri	*Ploceus philippinus*	22.06	59.89	Bushuev et al. (unpublished)
Aves	Passeriformes	Passeri	*Poecile atricapillus*	10.30	45.50	Rising & Hudson 1974
Aves	Passeriformes	Passeri	*Poecile montanus*	11.60	49.50	Reinertsen & Haftorn 1986

Aves	Passeriformes	Passeri	*Pogonotriccus ophthalmicus*	8.80	43.64	Londono et al. 2015
Aves	Passeriformes	Passeri	*Poliocrania exsul*	28.30	52.00	Wiesma et al. 2007
Aves	Passeriformes	Passeri	*Pomatorhinus hypoleucos*	83.80	148.65	Bushuev et al. (unpublished)
Aves	Passeriformes	Passeri	*Pomatorhinus schisticeps*	36.00	75.16	Bushuev et al. (unpublished)
Aves	Passeriformes	Passeri	*Pomatostomus ruficeps*	45.40	79.20	Bech et al. 2016
Aves	Passeriformes	Passeri	*Pomatostomus superciliosus*	36.10	61.80	Bech et al. 2016
Aves	Passeriformes	Passeri	*Pooecetes gramineus*	21.50	49.00	Yarbrough 1971
Aves	Passeriformes	Passeri	*Premnoplex brunnescens*	15.70	58.18	Londono et al. 2015
Aves	Passeriformes	Passeri	*Prinia flaviventris*	7.16	26.78	Bushuev et al. (unpublished)
Aves	Passeriformes	Passeri	*Prinia hodgsonii*	7.21	33.58	Bushuev et al. (unpublished)
Aves	Passeriformes	Passeri	*Prinia inornata*	10.25	41.04	Bushuev et al. (unpublished)
Aves	Passeriformes	Passeri	*Progne chalybea*	34.90	84.00	Wiersma et al. 2007
Aves	Passeriformes	Passeri	*Prosthemadera novaeseelandiae*	144.20	199.00	McNab 2009.
Aves	Passeriformes	Passeri	*Protonotaria citrea*	12.80	36.00	Yarbrough 1971
Aves	Passeriformes	Passeri	*Prunella modularis*	16.80	58.50	Kendeigh et al. 1977
Aves	Passeriformes	Passeri	*Psaltriparus minimus*	5.50	23.00	Chaplin 1982
Aves	Passeriformes	Passeri	*Pseudocolaptes boissonneautii*	42.90	100.00	Londono et al. 2015
Aves	Passeriformes	Passeri	*Psophodes olivaceus*	61.00	127.20	Bech et al. 2016
Aves	Passeriformes	Passeri	*Ptiloprora guisei*	20.70	52.50	McNab 2009.
Aves	Passeriformes	Passeri	*Ptiloris magnificus*	179.40	270.00	McNab 2005
Aves	Passeriformes	Passeri	*Pycnonotus atriceps*	21.54	52.37	Bushuev et al. (unpublished)
Aves	Passeriformes	Passeri	*Pycnonotus aurigaster*	27.00	57.02	Bushuev et al. (unpublished)
Aves	Passeriformes	Passeri	*Pycnonotus barbatus*	40.30	86.00	Seavy & McNab 2007
Aves	Passeriformes	Passeri	*Pycnonotus blanfordi*	28.83	55.29	Bushuev et al. (unpublished)
Aves	Passeriformes	Passeri	*Pycnonotus finlaysoni*	25.91	54.70	Bushuev et al. (unpublished)
Aves	Passeriformes	Passeri	*Pycnonotus finlaysoni*	26.30	40.50	Hails 1983
Aves	Passeriformes	Passeri	*Pycnonotus goiavier*	28.60	44.00	Hails 1983
Aves	Passeriformes	Passeri	*Pycnonotus jocosus*	32.90	64.20	Bech et al. 2016
Aves	Passeriformes	Passeri	*Pycnonotus melanicterus*	22.93	52.85	Bushuev et al. (unpublished)
Aves	Passeriformes	Passeri	*Pyriglena leuconota*	32.10	100.00	Londono et al. 2015
Aves	Passeriformes	Passeri	*Pyrrhocorax graculus*	206.40	308.50	Kendeigh et al. 1977
Aves	Passeriformes	Passeri	*Pyrrhomyias cinnamomeus*	10.70	36.36	Londono et al. 2015
Aves	Passeriformes	Passeri	*Pyrrhula pyrrhula*	30.40	99.50	Kendeigh et al. 1977
Aves	Passeriformes	Passeri	*Quiscalus mexicanus*	137.30	203.00	Wiersma et al. 2007
Aves	Passeriformes	Passeri	*Quiscalus quiscula*	92.20	178.00	Wiersma et al. 2007
Aves	Passeriformes	Passeri	*Ramphocelus carbo*	26.50	52.73	Londono et al. 2015
Aves	Passeriformes	Passeri	*Ramphocelus dimidiatus*	26.40	66.00	Wiersma et al. 2007
Aves	Passeriformes	Passeri	*Ramphocelus flammigerus*	32.00	75.00	Wiersma et al. 2007
Aves	Passeriformes	Passeri	*Ramphotrigon ruficauda*	17.40	50.91	Londono et al. 2015
Aves	Passeriformes	Passeri	*Regulus regulus*	5.50	33.00	Kendeigh et al. 1977
Aves	Passeriformes	Passeri	*Rhegmatorhina melanosticta*	42.20	74.55	Londono et al., 2015
Aves	Passeriformes	Passeri	*Rhipidura albolimbata*	9.70	29.50	McNab 2009.
Aves	Passeriformes	Passeri	*Rhipidura atra*	11.00	55.50	McNab 2009.
Aves	Passeriformes	Passeri	*Rhipidura fuliginosa*	8.90	29.40	Bech et al. 2016
Aves	Passeriformes	Passeri	*Rhynchocyclus fulvipectus*	26.00	70.91	Londono et al. 2015
Aves	Passeriformes	Passeri	*Rhynchocyclus olivaceus*	21.00	54.00	Wiersma et al. 2007
Aves	Passeriformes	Passeri	*Riparia riparia*	13.60	42.00	Kendeigh et al. 1977
Aves	Passeriformes	Passeri	*Rupicola peruvianus*	246.50	196.36	Londono et al., 2015
Aves	Passeriformes	Passeri	*Saltator coerulescens*	47.00	70.00	Bosque et al. 1999
Aves	Passeriformes	Passeri	*Saltator grossus*	49.50	80.00	Londono et al. 2015
Aves	Passeriformes	Passeri	*Saltator maximus*	44.80	98.50	Wiersma et al. 2007

Aves	Passeriformes	Passeri	*Saltator orenocensis*	32.70	56.50	Bosque et al. 1999
Aves	Passeriformes	Passeri	*Saltator striatipectus*	42.10	91.50	Wiersma et al. 2007
Aves	Passeriformes	Passeri	*Saxicola caprata*	13.45	36.80	Bushuev et al. (unpublished)
Aves	Passeriformes	Passeri	*Saxicola rubetra*	14.30	43.50	Kendeigh et al. 1977
Aves	Passeriformes	Passeri	*Saxicola stejnegeri*	11.56	48.59	Bushuev et al. (unpublished)
Aves	Passeriformes	Passeri	*Saxicola torquatus*	16.50	46.00	Klaassen 1995
Aves	Passeriformes	Passeri	*Sayornis phoebe*	21.60	62.00	Yarbrough 1971
Aves	Passeriformes	Passeri	*Sciaphylax hemimelaena*	18.00	45.45	Londono et al. 2015
Aves	Passeriformes	Passeri	*Sclerurus caudacutus*	36.20	83.64	Londono et al. 2015
Aves	Passeriformes	Passeri	*Sclerurus mexicanus*	28.80	80.00	Londono et al. 2015
Aves	Passeriformes	Passeri	*Scytalopus atratus*	21.50	69.09	Londono et al. 2015
Aves	Passeriformes	Passeri	*Scytalopus parvirostris*	17.00	65.45	Londono et al. 2015
Aves	Passeriformes	Passeri	*Seiurus aurocapilla*	19.00	43.50	Yarbrough 1971
Aves	Passeriformes	Passeri	*Sericornis frontalis*	11.00	33.00	Ambrose & Bradshaw 1988
Aves	Passeriformes	Passeri	*Sericornis frontalis*	13.20	57.60	Bech et al. 2016
Aves	Passeriformes	Passeri	*Sericornis humilis*	14.60	37.00	Bradshaw 1988 McNab, 2009
Aves	Passeriformes	Passeri	*Sericornis magnirostra*	8.70	36.60	Bech et al. 2016
Aves	Passeriformes	Passeri	*Sericornis nouhuysi*	16.60	50.50	McNab 2009.
Aves	Passeriformes	Passeri	*Serinus canaria*	13.30	41.00	Gavrilov, Dolnik 1985
Aves	Passeriformes	Passeri	*Setophaga americana*	7.00	22.00	Yarbrongh 1971
Aves	Passeriformes	Passeri	*Setophaga citrina*	12.00	38.50	Yarbrough 1971
Aves	Passeriformes	Passeri	*Setophaga coronata*	11.50	34.00	Yarbrough 1971
Aves	Passeriformes	Passeri	*Setophaga dominica*	9.80	29.00	Yarbrough 1971
Aves	Passeriformes	Passeri	*Setophaga palmarum*	9.80	28.00	Yarbrough 1971
Aves	Passeriformes	Passeri	*Setophaga petechia*	12.20	25.00	Wiersma et al. 2007
Aves	Passeriformes	Passeri	*Setophaga pinus*	12.00	32.00	Yarbrough 1971
Aves	Passeriformes	Passeri	*Sialia mexicana*	27.50	76.00	Mock 1991
Aves	Passeriformes	Passeri	*Silvicultrix pulchella*	12.00	30.91	Londono et al. 2015
Aves	Passeriformes	Passeri	*Silvicultrix spodionota*	10.70	30.91	Londono et al. 2015
Aves	Passeriformes	Passeri	*Sitta canadensis*	11.20	42.00	Mugas, Templeton 1970
Aves	Passeriformes	Passeri	*Sitta carolinensis*	18.30	52.00	Liknes & Swanson 1996
Aves	Passeriformes	Passeri	*Sittasomus griseicapillus*	14.40	49.09	Londono et al. 2015
Aves	Passeriformes	Passeri	*Smicrornis brevirostris*	5.90	29.40	Bech et al. 2016
Aves	Passeriformes	Passeri	*Spinus magellanicus*	11.60	36.36	Londono et al. 2015
Aves	Passeriformes	Passeri	*Spinus spinus*	14.00	52.50	Kendeigh et al. 1977
Aves	Passeriformes	Passeri	*Spinus tristis*	12.80	54.50	Dawson & Carey 1976
Aves	Passeriformes	Passeri	*Spizella passerina*	11.90	35.00	Yarbrough 1971
Aves	Passeriformes	Passeri	*Spizelloides arborea*	16.60	59.50	Losiewski, Dawson 1967
Aves	Passeriformes	Passeri	*Sporophila americana*	10.20	35.50	Wiersma et al. 2007
Aves	Passeriformes	Passeri	*Sporophila castaneiventris*	8.50	36.36	Londono et al. 2015
Aves	Passeriformes	Passeri	*Sporophila nigricollis*	8.90	36.36	Londono et al. 2015
Aves	Passeriformes	Passeri	*Stelgidopteryx ruficollis*	14.00	40.00	Londono et al. 2015
Aves	Passeriformes	Passeri	*Stelgidopteryx ruficollis*	11.90	28.50	Wiersma et al. 2007
Aves	Passeriformes	Passeri	*Strepera graculina*	306.70	339.00	Bech et al. 2016
Aves	Passeriformes	Passeri	*Sturnus vulgaris*	57.50	105.60	Bech et al. 2016
Aves	Passeriformes	Passeri	*Sturnus vulgaris*	75.00	158.00	Kendeigh et al. 1977
Aves	Passeriformes	Passeri	*Sylvia atricapilla*	21.90	75.00	Kendeigh et al. 1977
Aves	Passeriformes	Passeri	*Sylvia borin*	24.80	75.00	Kendeigh et al. 1977
Aves	Passeriformes	Passeri	*Sylvia curruca*	10.60	36.00	Kendeigh et al. 1977
Aves	Passeriformes	Passeri	*Sylvia nisoria*	21.30	69.00	Kendeigh et al. 1977
Aves	Passeriformes	Passeri	*Symposiachrus axillaris*	17.50	90.50	McNab, pers. obs.

Aves	Passeriformes	Passeri	*Synallaxis azarae*	13.60	41.82	Londono et al. 2015
Aves	Passeriformes	Passeri	*Synallaxis cabanisi*	19.30	54.55	Londono et al. 2015
Aves	Passeriformes	Passeri	*Syndactyla ucayalae*	51.20	110.91	Londono et al. 2015
Aves	Passeriformes	Passeri	*Tachycineta albilinea*	11.70	45.00	Wiersma et al. 2007
Aves	Passeriformes	Passeri	*Tachycineta bicolor*	16.40	51.50	Wiersma et al. 2007
Aves	Passeriformes	Passeri	*Tachyphonus luctuosus*	12.50	58.18	Londono et al. 2015
Aves	Passeriformes	Passeri	*Taeniopygia guttata*	11.70	38.40	Bech et al. 2016
Aves	Passeriformes	Passeri	*Taeniopygia guttata*	11.70	37.00	Calder 1964
Aves	Passeriformes	Passeri	*Tangara arthus*	22.40	67.27	Londono et al. 2015
Aves	Passeriformes	Passeri	*Tangara cyanicollis*	16.30	63.64	Londono et al. 2015
Aves	Passeriformes	Passeri	*Tangara larvata*	16.20	42.50	Wiersma et al. 2007
Aves	Passeriformes	Passeri	*Tangara vassorii*	18.50	41.82	Londono et al. 2015
Aves	Passeriformes	Passeri	*Tangara xanthogastra*	15.10	38.18	Londono et al. 2015
Aves	Passeriformes	Passeri	*Tarsiger cyanurus*	14.80	42.50	Gavrilov & Dolnik 1985
Aves	Passeriformes	Passeri	*Telespiza cantans*	31.60	76.50	Weathers & van Riper 1982
Aves	Passeriformes	Passeri	*Telespiza cantans*	32.80	79.50	Weathers, van Riper 1982
Aves	Passeriformes	Passeri	*Tephrodornis virgatus*	35.13	88.38	Bushuev et al. (unpublished)
Aves	Passeriformes	Passeri	*Terenotriccus erythrurus*	9.30	43.64	Londono et al. 2015
Aves	Passeriformes	Passeri	*Terpsiphone affinis*	17.00	43.35	Bushuev et al. (unpublished)
Aves	Passeriformes	Passeri	*Thamnomanes ardesiacus*	19.30	49.09	Londono et al. 2015
Aves	Passeriformes	Passeri	*Thamnomanes schistogynus*	19.70	61.82	Londono et al., 2015
Aves	Passeriformes	Passeri	*Thamnophilus atrinucha*	20.50	50.50	Wiersma et al. 2007
Aves	Passeriformes	Passeri	*Thamnophilus doliatus*	26.20	51.50	Wiersma et al. 2007
Aves	Passeriformes	Passeri	*Thamnophilus palliatus*	27.80	89.09	Londono et al., 2015
Aves	Passeriformes	Passeri	*Thamnophilus punctatus*	21.00	62.00	Vleck & Vleck 1979
Aves	Passeriformes	Passeri	*Thlypopsis ruficeps*	11.90	41.82	Londono et al. 2015
Aves	Passeriformes	Passeri	*Thlypopsis sordida*	14.90	56.36	Londono et al. 2015
Aves	Passeriformes	Passeri	*Thraupis bonariensis*	34.80	101.82	Londono et al. 2015
Aves	Passeriformes	Passeri	*Thraupis cyanocephala*	38.20	83.64	Londono et al. 2015
Aves	Passeriformes	Passeri	*Thraupis episcopus*	30.40	72.00	Wiersma et al. 2007
Aves	Passeriformes	Passeri	*Thraupis palmarum*	32.60	71.00	Wiersma et al. 2007
Aves	Passeriformes	Passeri	*Thripadectes holostictus*	42.50	69.09	Londono et al. 2015
Aves	Passeriformes	Passeri	*Thripadectes melanorhynchus*	47.50	98.18	Londono et al., 2015
Aves	Passeriformes	Passeri	*Thryophilus rufalbus*	22.60	53.50	Wiersma et al. 2007
Aves	Passeriformes	Passeri	*Thryothorus ludovicianus*	14.90	64.50	Eberhardt 1994
Aves	Passeriformes	Passeri	*Tiaris canorus*	7.60	28.00	Gavrilov 1997
Aves	Passeriformes	Passeri	*Tiaris obscurus*	12.10	29.09	Londono et al. 2015
Aves	Passeriformes	Passeri	*Timalia pileata*	20.62	43.50	Bushuev et al. (unpublished)
Aves	Passeriformes	Passeri	*Todirostrum cinereum*	7.30	30.50	Wiersma et al. 2007
Aves	Passeriformes	Passeri	*Toxorhamphus poliopterus*	13.20	34.50	McNab, pers. obs.
Aves	Passeriformes	Passeri	*Trichothraupis melanops*	24.90	81.82	Londono et al. 2015
Aves	Passeriformes	Passeri	*Troglodytes aedon*	10.20	37.50	Wiersma et al. 2007
Aves	Passeriformes	Passeri	*Troglodytes aedon*	13.30	38.00	Wiersma et al. 2007
Aves	Passeriformes	Passeri	*Troglodytes solstitialis*	13.00	49.09	Londono et al. 2015
Aves	Passeriformes	Passeri	*Troglodytes troglodytes*	8.90	30.00	Lindstrom & Kvist 1995
Aves	Passeriformes	Passeri	*Turdus albicollis*	51.30	83.64	Londono et al. 2015
Aves	Passeriformes	Passeri	*Turdus chiguanco*	87.00	98.18	Londono et al. 2015
Aves	Passeriformes	Passeri	*Turdus fuscater*	138.00	174.55	Londono et al. 2015
Aves	Passeriformes	Passeri	*Turdus grayi*	77.90	122.00	Wiersma et al. 2007
Aves	Passeriformes	Passeri	*Turdus hauxwelli*	63.30	90.91	Londono et al. 2015
Aves	Passeriformes	Passeri	*Turdus ignobilis*	56.60	74.55	Londono et al. 2015

Aves	Passeriformes	Passeri	*Turdus iliacus*	58.00	130.00	Kendeigh et al. 1977
Aves	Passeriformes	Passeri	*Turdus lawrencii*	55.30	121.82	Londono et al. 2015
Aves	Passeriformes	Passeri	*Turdus leucops*	62.20	129.09	Londono et al. 2015
Aves	Passeriformes	Passeri	*Turdus merula*	85.10	172.80	Bech et al. 2016
Aves	Passeriformes	Passeri	*Turdus merula*	82.60	167.00	Kendeigh et al. 1977
Aves	Passeriformes	Passeri	*Turdus migratorius*	62.40	137.00	Wiermsa et al. 2007
Aves	Passeriformes	Passeri	*Turdus nigriceps*	51.10	96.36	Londono et al. 2015
Aves	Passeriformes	Passeri	*Turdus philomelos*	62.80	130.50	Gavrilov & Dolnik 1985
Aves	Passeriformes	Passeri	*Turdus poliocephalus*	66.10	128.00	McNab, pers. ob.
Aves	Passeriformes	Passeri	*Turdus serranus*	81.20	176.36	Londono et al., 2015
Aves	Passeriformes	Passeri	*Turdus viscivorus*	108.20	198.50	Kendeigh et al. 1977
Aves	Passeriformes	Passeri	*Tyrannus melancholicus*	38.00	65.50	Wiersma et al. 2007
Aves	Passeriformes	Passeri	*Tyrannus tyrannus*	35.70	78.50	Yarbrough 1971
Aves	Passeriformes	Passeri	*Uraeginthus bengalus*	9.20	29.50	Kendeigh et al. 1977
Aves	Passeriformes	Passeri	*Vermivora cyanoptera*	7.80	27.00	Yarbrough 1971
Aves	Passeriformes	Passeri	*Vidua paradisaea*	10.50	35.00	Terroine, Trautman 1927
Aves	Passeriformes	Passeri	*Vireo flavoviridis*	15.90	50.50	Wiersma et al. 2007
Aves	Passeriformes	Passeri	*Vireo olivaceus*	16.20	43.00	Wiersma et al. 2007
Aves	Passeriformes	Passeri	*Willisornis poecilinotus*	22.30	70.91	Londono et al. 2015
Aves	Passeriformes	Passeri	*Xenops minutus*	12.70	32.73	Londono et al. 2015
Aves	Passeriformes	Passeri	*Xenops minutus*	9.90	41.00	Wiersma et al. 2007
Aves	Passeriformes	Passeri	*Xiphorhynchus elegans*	41.40	92.73	Londono et al. 2015
Aves	Passeriformes	Passeri	*Xiphorhynchus guttatus*	60.10	94.55	Londono et al. 2015
Aves	Passeriformes	Passeri	*Xiphorhynchus guttatus*	45.20	80.50	Vleck & Vleck 1979
Aves	Passeriformes	Passeri	*Xiphorhynchus triangularis*	44.10	109.09	Londono et al. 2015
Aves	Passeriformes	Passeri	*Zimmerius bolivianus*	11.00	50.91	Londono et al. 2015
Aves	Passeriformes	Passeri	*Zonotrichia albicollis*	20.20	50.00	Yarbrough 1971
Aves	Passeriformes	Passeri	*Zonotrichia capensis*	22.20	56.36	Londono et al., 2015
Aves	Passeriformes	Passeri	*Zonotrichia leucophrys*	26.10	60.50	Yarbrough 1971
Aves	Passeriformes	Passeri	*Zonotrichia querula*	33.30	88.50	Yarbrough 1971
Aves	Passeriformes	Passeri	*Zoothera dauma*	107.00	143.11	Bushuev et al. (unpublished)
Aves	Passeriformes	Passeri	*Zoothera lunulata*	113.20	184.20	Bech et al. 2016
Aves	Passeriformes	Passeri	*Zosterops lateralis*	10.40	37.80	Bech et al. 2016

References to Table 2 Avian BMR

Adams N.J., Brown C.R. 1984. Metabolic rates of sub-antarctic Procellariiformes: a comparative study. Comparative Biochemistry and Physiology Part A: Physiology 77: 169-173.

Ambrose S.J., Bradshaw S.D. 1988. Seasonal changes in standard metabolic rates in the white-browed scrubwren *Sericornis frontalis* (Acanthizidae) from arid, semi-arid and mesic environments. Comparative Biochemistry and Physiology Part A: Physiology 89: 79-83.

Bartholomew G.A., Hudson J.W., Howell T.R. 1962. Body temperature, oxygen consumption, evaporative water loss and heart rate in the Poor-will. The Condor 64: 117-125.

Bartholomew G.A., Vleck C.M., Bucher T.L. 1983. Energy metabolism and nocturnal hypothermia in two tropical passerine frugivores, *Manacus vitellinus* and *Pipra mentalis*. Physiological Zoology 56: 370-379.

Baudinette R., Gill P., O'driscoll M. 1986. Energetics of the Little Penguin, *Eudyptula minor*: temperature regulation, the calorigenic effect of food, and moulting. Australian Journal of Zoology 34: 35-45.

Bech C. 1980. Body temperature, metabolic rate, and insulation in winter and summer acclimatized mute swans (*Cygnus olor*). Journal of Comparative Physiology 136: 61-66.

Bech C., Chappell M.A., Astheimer L.B., Londoño G.A., Buttemer W.A. 2016. A ‘slow pace of life’ in Australian old-endemic passerine birds is not accompanied by low basal metabolic rates. Journal of Comparative Physiology. B: Biochemical, Systemic, and Environmental Physiology 186: 503-512.

Bech C., Nicol S.C. 1999. Thermoregulation and ventilation in the tawny frogmouth, *Podargus strigoides*: a low-metabolic avian species. Australian Journal of Zoology 47: 143-153.
Benedict F.G., Fox E.L. 1927. The gaseous metabolism of large wild birds under aviary life. Proceedings of the American Philosophical Society 66: 511-534.
Benedict F.G., Riddle O. 1929. The measurement of the basal heat production of pigeons: II. Physiological technique. The Journal of Nutrition 1: 497-536.
Boismenu C., Gauthier G., Larochelle J. 1992. Physiology of prolonged fasting in greater snow geese (*Chen caerulescens atlantica*). The Auk 109: 511-521.
Boix-Hinzen C., Lovegrove B.G. 1998. Circadian metabolic and thermoregulatory patterns of red-billed woodhoopoes (*Phoeniculus purpureus*): the influence of huddling. Journal of Zoology 244: 33-41.
Bonaccorso F.J., McNab B.K. 2003. Standard energetics of leaf-nosed bats (Hipposideridae): its relationship to intermittent- and protracted-foraging tactics in bats and birds. Journal of Comparative Physiology B 173: 43-53.
Bosque C., Pacheco M.A., Siegel R.B. 1999. Maintenance energy costs of two partially folivorous tropical passerines. The Auk 116: 246-252.
Brown C.R. 1984. Resting metabolic rate and energetic cost of incubation in macaroni penguins *Eudyptes chrysolophus* and rockhopper penguins *E. chrysocome*. Comparative Biochemistry and Physiology Part A: Physiology 77: 345-350.
Brown C.R. 1988. Energy expenditure during incubation in four species of sub-Antarctic burrowing petrels. Ostrich 59: 67-70.
Brown C.R., Adams N.J. 1984. Basal metabolic rate and energy expenditure during incubation in the Wandering Albatross (*Diomedea exulans*). The Condor 86: 182-186.
Bryant D.M., Furness R.W. 1995. Basal metabolic rates of North Atlantic seabirds. Ibis 137: 219-226.
Bryant D.M., Hails C.J., Tatner P. 1984. Reproductive energetics of two tropical bird species. The Auk 101: 25-37.
Bucher T.L. 1981. Oxygen consumption, ventilation and respiratory heat loss in a parrot, *Bolborhynchus lineola*, in relation to ambient temperature. Journal of Comparative Physiology 142: 479-488.
Bushuev A, Tolstenkov O, Zubkova E, Solovyeva E, & Kerimov A (2018). Basal metabolic rate in free-living tropical birds: the influence of phylogenetic, behavioral, and ecological factors. *Current Zoology,* **64**, 33-43.
Bushuev A, Tolstenkov O, Zubkova E, Solovyeva E, & Kerimov A (in press). Basal metabolic rate in free-living tropical birds: the influence of phylogenetic, behavioral, and ecological factors.
Buttemer W.A., Nicol S.C., Sharman A. 2003. Thermoenergetics of pre-moulting and moulting kookaburras (*Dacelo novaeguineae*): they're laughing. Journal of Comparative Physiology B 173: 223-230.
Calder W.A. 1964. Gaseous metabolism and water relations of the zebra finch, *Taeniopygia castanotis*. Physiological Zoology 37: 400-413.
Carpenter F.L. 1976. Ecology and Evolution of an Andean Hummingbird (*Oreotrochilus estella*). University of California Publications in Zoology
Chaplin S.B. 1982. The energetic significance of huddling behavior in common bush tits (*Psaltriparus minimus*). The Auk 99: 424-430.
Collins B.G., Cary G., Payne S. 1980. Metabolism, thermoregulation and evaporative water loss in two species of Australian nectar-feeding birds (family Meliphagidae). Comparative Biochemistry and Physiology Part A: Physiology 67: 629-635.
Coulombe H.N. 1970. Physiological and physical aspects of temperature regulation in the burrowing owl *Speotyto cunicularia*. Comparative Biochemistry and Physiology 35: 307-337.
Crawford E.C., Jr., Lasiewski R.C. 1968. Oxygen consumption and respiratory evaporation of the emu and rhea. The Condor 70: 333-339.
Daan S., Masman D., Groenewold A. 1990. Avian basal metabolic rates: their association with body composition and energy expenditure in nature. American Journal of Physiology 259: R333-R340.
Dawson W. 1954. Temperature regulation and water requirements of the brown and abert towhees, *Pipilo fuscus* and *Pipilo aberti*. University of California Publications in Zoology 59: 81-124.
Dawson W.R., Bennett A.F. 1973. Roles of metabolic level and temperature regulation in the adjustment of Western plumed pigeons (*Lophophaps ferruginea*) to desert conditions. Comparative Biochemistry and Physiology Part A: Physiology 44: 249-266.
Dawson W.R., Carey C. 1976. Seasonal acclimatization to temperature in cardueline finches. Journal of Comparative Physiology 112: 317-333.
Dawson W.R., Fisher C.D. 1969. Responses to temperature by the spotted nightjar (*Eurostopodus guttatus*). The Condor 71: 49-53.

Dawson W.R., Tordoff H.B. 1964. Relation of oxygen consumption to temperature in the red and white-winged crossbills. The Auk 81: 26-35.
Drent R.H., Stonehouse B. 1971. Thermoregulatory responses of the peruvian penguin, *Spheniscus humboldti*. Comparative Biochemistry and Physiology Part A: Physiology 40: 689-710.
Eberhardt L.S. 1994. Oxygen consumption during singing by male Carolina Wrens (*Thryothorus ludovicianus*). The Auk 111: 124-130.
Edwards T.C. 1987. Standard rate of metabolism in the Common Barn-Owl (*Tyto alba*). The Wilson Bulletin 99: 704-706.
Ehlers R., Morton M.L. 1982. Metabolic rate and evaporative water loss in the least seed-snipe, *Thinocorus rumicivorus*. Comparative Biochemistry and Physiology Part A: Physiology 73: 233-235.
Ellis H. 1980. Metabolism and solar radiation in dark and white herons in hot climates. Physiological Zoology 53: 358-372.
Ellis H.I., Jehl J.R. 2003. Temperature regulation and the constraints of climate in the Eared Grebe. Waterbirds: The International Journal of Waterbird Biology 26: 275-279.
Enger P.S. 1957. Heat regulation and metabolism in some tropical mammals and birds. Acta Physiologica Scandinavica 40: 161-166.
Gabrielsen G.W., Taylor J.R.E., Konarzewski M., Mehlum F. 1991. Field and laboratory metabolism and thermoregulation in Dovekies (*Alle alle*). The Auk 108: 71-78.
Ganey J.L., Balda R.P., King R.M. 1993. Metabolic rate and evaporative water loss of Mexican Spotted and Great Horned Owls. Wilson Bulletin 105: 645-656.
Gavrilov V.M. 1977. Energetics of penguins [in Russian]. In: Il'ichev V.D. (ed) Adaptatsii Pingvinov (Adaptations of Penguins). Nauka, Moscow, pp. 102-110.
Gavrilov V.M. 1997. Energetics and avian behavior (Physiology and General Biology Reviews. Vol. 11). Harwood Academic Publishers, Amsterdam.
Gavrilov V.M., Dolnik V.R. 1985: Basal metabolic rate, thermoregulation and existence energy in birds: world data. pp. 421-466 in Ilyichov V.D., Gavrilov V.M., eds. Acta XVIII Congressus Internationalis Ornithologici. Nauka, Moscow. Gavrilov V.M., Dolnik V.R. 1985. Basal metabolic rate, thermoregulation and existence energy in birds: world data. Nauka, Moscow.
Gavrilov V.V. 1996. Standard metabolism, energy of life and energy cost of non-flight activities in the Cuneate-tailed Gull *Rhodostethia rosea* (Laridae, Aves). Biology Bulletin 23: 55-59.
Gerald R., Robert B. 1973. Standard metabolic rate and lower critical temperature for the Ruffed Grouse. The Wilson Bulletin 85: 223-229.
Gessaman J.A. 1972. Bioenergetics of the snowy owl (*Nyctea scandiaca*). Arctic and Alpine Research 4: 223-238.
Giaja J., Males B. 1928. Sur le valeur du métabolisme de base de quelques animaux en fonction de leur surface. Annales de Physiologie et de Physicochemie Biologique 4: 875-904.
Goldstein R.B. 1974. Relation of metabolism to ambient temperature in the Verdin. The Condor 76: 116-119.
Graber R.R. 1962. Food and oxygen consumption in three species of owls (Strigidae). The Condor 64: 473-487.
Grajal A. 1991: Nutritional ecology and digestive physiology of the hoatzin, *Opisthocomus hoazin*, a folivorous bird with foregut fermentation. University of Florida, Gainesville, Florida, US. Grajal A. 1991. Nutritional ecology and digestive physiology of the hoatzin, *Opisthocomus hoazin*, a folivorous bird with foregut fermentation, University of Florida, Gainesville, Florida, US.
Grant G.S., Whittow G.C. 1983. Metabolic cost of incubation in the Laysan albatross and Bonin petrel. Comparative Biochemistry and Physiology, Part A: Physiology 74: 77-82.
Hails C.J. 1983. The metabolic rate of tropical birds. The Condor 85: 61-65.
Hainsworth F.R., Wolf L.L. 1970. Regulation of oxygen consumption and body temperature during torpor in a hummingbird, *Eulampis jugularis*. Science 168: 368-369.
Hayworth A.M., Weathers W.W. 1984. Temperature regulation and climatic adaptation in black-billed and yellow-billed magpies. The Condor 86: 19-26.
Hennemann III W.W. 1983. Environmental influences on the energetics and behavior of anhingas and double-crested cormorants. Physiological Zoology 56: 201-216.
Herzog D. 1930. Untersuchungen über den Grundumsatz der Vögel. Wissenschaftliches Archiv für Landwirtschaft 3: 601-626.
Hinds D.S., Calder W.A. 1973. Temperature regulation of the Pyrrhuloxia and the Arizona Cardinal. Physiological Zoology 46: 55-71.
Hinsley S.A., Ferns P.N., Thomas D.H., Pinshow B. 1993. Black-Bellied Sandgrouse (*Pterocles orientalis*) and Pin-Tailed Sandgrouse (Pterocles alchata): closely related species with differing bioenergetic adaptations to arid zones. Physiological Zoology 66: 20-42.

Hoffmann R., Prinzinger R. 1984. Torpor und Nahrungsausnutzung bei 4 Mausvogelarten (Coliiformes). Journal für Ornithologie 125: 225-237.

Hohtola E., Pyörnilä A., Rintamäki H. 1994. Fasting endurance and cold resistance without hypothermia in a small predatory bird: the metabolic strategy of Tengmalm's owl, *Aegolius funereus*. Journal of Comparative Physiology B 164: 430-437.

Holmes R.T., Sawyer R.H. 1975. Oxygen consumption in relation to ambient temperature in five species of forest-dwelling thrushes (*Hylocichla* and *Catharus*). Comparative Biochemistry and Physiology Part A: Physiology 50: 527-531.

Hudson J.W., Brush A.H. 1964. A comparative study of the cardiac and metabolic performance of the dove, *Zenaidura macroura*, and the quail, *Lophortyx californicus*. Comparative Biochemistry and Physiology 12: 157-170.

Irving L., Krog H., Manson M. 1955. The metabolism of some Alaskan animals in winter and summer. Physiological Zoology 28: 173-185.

Iversen J.A., Krog J. 1972. Body temperature and resting metabolism rates in small petrels. Norwegian Journal of Zoology 20: 141-144.

Jenssen B.M., Ekker M., Bech C. 1989. Thermoregulation in winter-acclimatized common eiders (*Somateria mollissima*) in air and water. Canadian Journal of Zoology 67: 669-673.

Johnson R.E. 1968. Temperature regulation in the white-tailed ptarmigan, *Lagopus leucurus*. Comparative Biochemistry and Physiology 24: 1003-1014.

Johnson S.R., Cowan I.M. 1974. Thermal adaptation as a factor affecting colonizing success of introduced Sturnidae (Aves) in North America. Canadian Journal of Zoology 52: 1559-1576.

Johnson W.D., Collins C.T. 1975. Notes on the metabolism of the Cuckoo Owlet and Hawk Owl. Bulletin of the Southern California Academy of Sciences 74: 44-45.

Jos B.W. 1999. Heat production and evaporative water loss of Dune Larks from the Namib desert. The Condor 101: 432-438.

Kahl Jr. M.P. 1963. Thermoregulation in the Wood Stork, with special reference to the role of the legs. Physiological Zoology 36: 141-151.

Kendeigh S.C., Dolnik V.R., Gavrilov V.M. 1977. Avian energetics. In: Pinowski J., Kendeigh S.C. (eds). Granivorous Birds in Ecosystems. Cambridge University Press, Cambridge, pp. 127-204.

Kersten M., Piersma T. 1987. High levels of energy expenditure in shorebirds: metabolic adaptations to an energetically expensive way of life. Ardea 75: 175-187.

Klaassen M. 1995. Molt and basal metabolic costs in males of two subspecies of stonechats: the European *Saxicola torquata rubicula* and the East African *S. t. axillaris*. Oecologia 104: 424-432.

Kooyman G.L., Gentry R.L., Bergman W.P., Hammel H.T. 1976. Heat loss in penguins during immersion and compression. Comparative Biochemistry and Physiology Part A: Physiology 54: 75-80.

Larochelle J., Delson J., Schmidt-Nielsen K. 1982. Temperature regulation in the Black Vulture. Canadian Journal of Zoology 60: 491-494.

Lasiewski R.C. 1963. Oxygen consumption of torpid, resting, active, and flying hummingbirds. Physiological Zoology 36: 122-140.

Lasiewski R.C., Dawson W.R. 1967. A re-examination of the relation between standard metabolic rate and body weight in birds. The Condor 69: 13-23.

Lasiewski R.C., Dawson W.R., Bartholomew G.A. 1970. Temperature regulation in the little Papuan frogmouth, *Podargus ocellatus*. The Condor 72: 332-338.

Lasiewski R.C., Hubbard S.H., Moberly W.R. 1964. Energetic relationships of a very small passerine bird. The Condor 66: 212-220.

Lasiewski R.C., Richard J.L. 1967. Physiological responses of the blue-throated and Rivoli's hummingbirds. The Auk 84: 34-48.

Lasiewski R.C., Weathers W.W., Bernstein M.H. 1967. Physiological responses of the giant hummingbird, *Patagona gigas*. Comparative Biochemistry and Physiology 23: 797-813.

Ligon J.D. 1968. The biology of the elf owl, *Micrathene whitneyi* (University of Michigan. Museum of Zoology. Miscellaneous publications. Museum of Zoology, University of Michigan.

Ligon J.D. 1969. Some aspects of temperature relations in small owls. The Auk 86: 458-472.

Liknes E.T., Swanson D.L. 1996. Seasonal variation in cold tolerance, basal metabolic rate, and maximal capacity for thermogenesis in White-breasted Nuthatches *Sitta carolinensis* and Downy Woodpeckers *Picoides pubescens*, two unrelated arboreal temperate residents. Journal of Avian Biology 27: 279-288.

Lindström Å., Kvist A. 1995. Maximum energy intake rate is proportional to basal metabolic rate in passerine birds. Proceedings of the Royal Society of London. Series B: Biological Sciences 261: 337-343.

Londoño G.A., Chappell M.A., Castañeda M.d.R., Jankowski J.E., Robinson S.K. 2015. Basal metabolism in tropical birds: latitude, altitude, and the “pace of life”. Functional Ecology 29: 338-346.
López-Calleja M.V., Bozinovic F. 1995. Maximum metabolic rate, thermal insulation and aerobic scope in a small-sized Chilean hummingbird (*Sephanoides sephanoides*). The Auk 112: 1034-1036.
Lovegrove B.G., Smith G.A. 2003. Is ‘nocturnal hypothermia’ a valid physiological concept in small birds?: a study on Bronze Mannikins *Spermestes cucullatus*. Ibis 145: 547-557.
MacMillen R.E. 1974. Bioenergetics of Hawaiian honeycreepers: the Amakihi (*Loxops virens*) and the Anianiau (*L. parva*). The Condor 76: 62-69.
MacMillen R.E. 1981. Nonconformance of standard metabolic rate with body mass in Hawaiian honeycreepers. Oecologia 49: 340-343.
MacMillen R.E., Carpenter F.L. 1977. Daily energy costs and body weight in nectarivorous birds. Comparative Biochemistry and Physiology Part A: Physiology 56: 439-441.
MacMillen R.E., Trost C.H. 1965. Oxygen consumption and water loss in the Inca dove, *Scardafella inca*. American Zoologist 5 (Abstracts of the summer meeting of the American Society of Zoologists. August 17-20, 1965. University of Illinois, Urbana, Illinois): 208-209.
MacMillen R.E., Trost C.H. 1967. Thermoregulation and water loss in the Inca dove. Comparative Biochemistry and Physiology 20: 263-273.
Maloney S.K., Dawson T.J. 1994. Thermoregulation in a large bird, the emu (*Dromaius novaehollandiae*). Journal of Comparative Physiology B 164: 464-472.
Marder J., Bernstein R. 1983. Heat balance of the partridge *Alectoris chukar* exposed to moderate, high and extreme thermal stress. Comparative Biochemistry and Physiology Part A: Physiology 74: 149-154.
Marschall U., Prinzinger R. 1991. Vergleichende Ökophysiologie von fünf Prachtfinkenarten (Estrildidae). Journal für Ornithologie 132: 319-323.
McKechnie A., Lovegrove B. 2001a. Heterothermic responses in the speckled mousebird (*Colius striatus*). Journal of Comparative Physiology B 171: 507-518.
McKechnie A.E., Körtner G., Lovegrove B.G. 2004. Rest-phase thermoregulation in free-ranging white-backed mousebirds. The Condor 106: 143-149.
McKechnie A.E., Lovegrove B.G. 2001b. Thermoregulation and the energetic significance of clustering behavior in the white-backed mousebird (*Colius colius*). Physiological and Biochemical Zoology 74: 238-249.
McKechnie A.E., Lovegrove B.G. 2003. Facultative hypothermic responses in an Afrotropical arid-zone passerine, the red-headed finch (*Amadina erythrocephala*). Journal of Comparative Physiology B 173: 339-346.
McNab B.K. 1996. Metabolism and temperature regulation of kiwis (Apterygidae). The Auk 113: 687-692.
McNab B.K. 2000. The influence of body mass, climate, and distribution on the energetics of South Pacific pigeons. Comparative Biochemistry and Physiology. Part A: Molecular & Integrative Physiology 127: 309-329.
McNab B.K. 2001. Energetics of toucans, a barbet, and a hornbill: implications for avian frugivory. The Auk 118: 916-933.
McNab B.K. 2003. The energetics of New Zealand's ducks. Comparative Biochemistry and Physiology Part A: Molecular & Integrative Physiology 135: 229-247.
McNab B.K. 2005. Food habits and the evolution of energetics in birds of paradise (Paradisaeidae). Journal of Comparative Physiology. B: Biochemical, Systemic, and Environmental Physiology 175: 117-132.
McNab B.K. 2009. Ecological factors affect the level and scaling of avian BMR. Comparative Biochemistry and Physiology. Part A: Molecular & Integrative Physiology 152: 22-45.
McNab B.K., Bonaccorso F.J. 1995. The energetics of Australasian swifts, frogmouths, and nightjars. Physiological Zoology 68: 245-261.
McNab B.K., Ellis H.I. 2006. Flightless rails endemic to islands have lower energy expenditures and clutch sizes than flighted rails on islands and continents. Comparative Biochemistry and Physiology Part A: Molecular & Integrative Physiology 145: 295-311.
McNab B.K., Salisbury C.A. 1995. Energetics of New Zealand's temperate parrots. New Zealand Journal of Zoology 22: 339-349.
Merola-Zwartjes M. 1998. Metabolic rate, temperature regulation, and the energetic implications of roost nests in the bananaquit (*Coereba flaveola*). The Auk 115: 780-786.
Merola-Zwartjes M., Ligon J.D. 2000. Ecological energetics of the Puerto Rican tody: heterothermy, torpor, and intra-island variation. Ecology 81: 990-1003.
Misch M.S. 1960. Heat regulation in the northern Blue Jay, *Cyanocitta cristata bromia* Oberholser. Physiological Zoology 33: 252-259.
Mock P.J. 1991. Daily allocation of time and energy of western bluebird feeding nestlings. The Condor 93: 598-611.

Mortensen A., Blix A.S. 1986. Seasonal changes in resting metabolic rate and mass specific conductance in Svalbard ptarmigan, Norwegian rock ptarmigan and Norwegian willow ptarmigan. Ornis Scandinavica 17: 8-13.
Mugaas J.N., Templeton J.R. 1970. Thermoregulation of the red-breasted nuthatch (*Sitta canadensis*). The Condor 72: 125-132.
Murrish D.E. 1970. Responses to temperature in the dipper, *Cinclus mexicanus*. Comparative Biochemistry and Physiology 34: 859-869.
Ohmart R.D., Lasiewski R.C. 1971. Roadrunners: energy conservation by hypothermia and absorption of sunlight. Science 172: 67-69.
Pekins P.J., Gessaman J.A., Lindzey F.G. 1992. Winter energy requirements of blue grouse. Canadian Journal of Zoology 70: 22-24.
Piersma T., Cadée N., Daan S. 1995. Seasonality in basal metabolic rate and thermal conductance in a long-distance migrant shorebird, the knot (*Calidris canutus*). Journal of Comparative Physiology B 165: 37-45.
Pinshow B., Fedak M.A., Battles D.R., Schmidt-Nielsen K. 1976. Energy expenditure for thermoregulation and locomotion in emperor penguins. American Journal of Physiology 231: 903-912.
Pinshow B., Fedak M.A., Schmidt-Nielsen K. 1977. Terrestrial locomotion in penguins: it costs more to waddle. Science 195: 592-594.
Prinzinger R. 1988. Energy metabolism, body-temperature and breathing parameters in nontorpid blue-naped mousebirds *Urocolius macrourus*. Journal of Comparative Physiology B 157: 801-806.
Prinzinger R., Göppel R., Lorenz A., Kulzer E. 1981. Body temperature and metabolism in the red-backed mousebird (*Colius castanotus*) during fasting and torpor. Comparative Biochemistry and Physiology Part A: Physiology 69: 689-692.
Prinzinger R., Hänssler I. 1980. Metabolism-weight relationship in some small nonpasserine birds. Experientia 36: 1299-1300.
Prinzinger R., Lübben I., Schuchmann K.L. 1989. Energy metabolism and body temperature in 13 sunbird species (Nectariniidae). Comparative Biochemistry and Physiology Part A: Physiology 92: 393-402.
Prinzinger R., Misovic A., Schleucher E. 1993. Energieumsatz und Körpertemperatur bei der Zwergwachtel (*Coturnix chinensis*) und beim Bindenlaufhühnchen (*Turnix suscitator*). Journal für Ornithologie 134: 79-84.
Reinertsen R.E., Haftorn S. 1986. Different metabolic strategies of northern birds for nocturnal survival. Journal of Comparative Physiology B 156: 655-663.
Rezende E.L., López-Calleja M.V., Bozinovic F. 2001. Standard and comparative energetics of a small avian herbivore (*Phytotoma rara*). The Auk 118: 781-785.
Ricklefs R.E., Matthew K.K. 1983. Rates of oxygen consumption in four species of seabird at Palmer Station, Antarctic Peninsula. Comparative Biochemistry and Physiology Part A: Physiology 74: 885-888.
Ricklefs R.E., White S.C., Cullen J. 1980. Energetics of postnatal growth in Leach's storm-petrel. The Auk 97: 566-575.
Rising J.D. 1969. A comparison of metabolism and evaporative water loss of Baltimore and Bullock orioles. Comparative Biochemistry and Physiology 31: 915-925.
Rising J.D., Hudson J.W. 1974. Seasonal variation in the metabolism and thyroid activity of the black-capped chickadee (*Parus atricapillus*). The Condor 76: 198-203.
Roberts J.R., Baudinette R.V. 1986. Thermoregulation, oxygen consumption and water turnover in stubble quail, *Coturnix pectoralis*, and king quail, *Coturnix chinensis*. Australian Journal of Zoology 34: 25-33.
Ryan P.G., Watkins B.P., Siegfried W.R. 1989. Morphometrics, metabolic rate and body temperature of the smallest flightless bird: the Inaccessible island rail. The Condor 91: 465-467.
Schleucher E. 2001. Heterothermia in pigeons and doves reduces energetic costs. Journal of Thermal Biology 26: 287-293.
Schleucher E. 2002. Metabolism, body temperature and thermal conductance of fruit-doves (Aves: Columbidae, Treroninae). Comparative Biochemistry and Physiology Part A: Molecular & Integrative Physiology 131: 417-428.
Schleucher E., Withers P.C. 2002. Metabolic and thermal physiology of pigeons and doves. Physiological and Biochemical Zoology: Ecological and Evolutionary Approaches 75: 439-450.
Scholander P.F. 1940. Experimental Investigations On the Respiratory Function In Diving Mammals and Birds: With 88 Figures In the Text. I kommisjon hos Jacob Dybwad, Oslo.
Scholander P.F., Hock R., Walters V., Johnson F., Irving L. 1950. Heat regulation in some arctic and tropical mammals and birds. Biological Bulletin 99: 237-258.
Seavy N.E. 2006. Physiological correlates of habitat association in East African sunbirds (Nectariniidae). Journal of Zoology 270: 290-297.
Seavy N.E., McNab B.K. 2007. Energetics of East African Pycnonotids. Biotropica 39: 114-119.

Terroine E.F., Trautmann S. 1927. Influence de la température extérieure sur la production calorique des homéothermes et loi des surfaces. Annales de Physiologie et de Physicochimie Biologique 3: 422-457.
Tieleman B.I., Williams J.B., Bloomer P. 2003. Adaptation of metabolism and evaporative water loss along an aridity gradient. Proceedings of the Royal Society of London. Series B: Biological Sciences 270: 207-214.
Trost C.H. 1972. Adaptations of horned larks (*Eremophila alpestris*) to hot environments. The Auk 89: 506-527.
Vander Haegen W.M., Owen R.B., Krohn W.B. 1994. Metabolic rate of american woodcock. The Wilson Bulletin 106: 338-343.
Veghte J.H. 1964. Thermal and metabolic responses of the gray jay to cold stress. Physiological Zoology 37: 316-328.
Vitali S., Withers P., Richardson K. 1999. Standard metabolic rates of three nectarivorous meliphagid passerine birds. Australian Journal of Zoology 47: 385-391.
Vleck C.M., Kenagy G.J. 1980. Embryonic metabolism of the Fork-tailed Storm Petrel: physiological patterns during prolonged and interrupted incubation. Physiological Zoology 53: 32-42.
Vleck C.M., Vleck D. 1979. Metabolic rate in five tropical bird species. The Condor 81: 89-91.
Wasser J.S. 1986. The relationship of energetics of falconiform birds to body mass and climate. The Condor 88: 57-62.
Weathers W.W. 1977. Temperature regulation in the dusky munia, *Lonchura fuscans* (Cassin) (Estrildidae). Australian Journal of Zoology 25: 193-199.
Weathers W.W. 1981. Physiological thermoregulation in heat-stressed birds: consequences of body size. Physiological Zoology 54: 345-361.
Weathers W.W., Caccamise D.F. 1978. Seasonal acclimatization to temperature in monk parakeets. Oecologia 35: 173-183.
Weathers W.W., Schoenbaechler D.C. 1976. Regulation of body temperature in the Budherygah, *Melopsittacus undulatus*. Australian Journal of Zoology 24: 39-47.
Weathers W.W., van Riper C., III. 1982. Temperature regulation in two endangered Hawaiian honeycreepers: the Palila (*Psittirostra bailleui*) and the Laysan finch (*Psittirostra cantans*). The Auk 99: 667-674.
Weathers W.W., Weathers D.L., van Riper C., III. 1983. Basal metabolism of the apapane: comparison of freshly caught birds with long-term captives. The Auk 100: 977-978.
West G.C., Hart J.S. 1966. Metabolic responses of evening grosbeaks to constant and to fluctuating temperatures. Physiological Zoology 39: 171-184.
Wiersma P., Muñoz-Garcia A., Walker A., Williams J.B. 2007. Tropical birds have a slow pace of life. Proceedings of the National Academy of Sciences of the United States of America 104: 9340-9345.
Wijnandts H. 1984. Ecological energetics of the long-eared owl (*Asio otus*). Ardea 55: 1-92.
Williams J.B., Hansell H. 1981. Bioenergetics of captive Belding's savannah sparrows (*Passerculus sanwichensis beldingi*). Comparative Biochemistry and Physiology Part A: Physiology 69: 783-787.
Williams J.B., Withers P.C., Bradshaw S.D., Nagy K.A. 1991. Metabolism and water flux of captive and free-living Australian parrots. Australian Journal of Zoology 39: 131-142.
Willoughby E.J. 1969. Evaporative water loss of a small xerophilous finch, *Lonchura malabarica.* Comparative Biochemistry and Physiology 28: 655-664.
Withers P.C. 1983. Energy, water, and solute balance of the ostrich *Struthio camelus*. Physiological Zoology 56: 568-579.
Withers P.C., Forbes R.B., Hedrick M.S. 1987. Metabolic, water and thermal relations of the Chilean tinamou. The Condor 89: 424-426.
Withers P.C., Williams J.B. 1990. Metabolic rate and respiratory physiology of an arid-adapted Australian bird, the Spinifex Pigeon. The Condor 92: 961-969.
Wunder B.A. 1979. Evaporative water loss from birds: effects of artificial radiation. Comparative Biochemistry and Physiology Part A: Physiology 63: 493-494.
Wunder B.A., Trebella J.J. 1976. Effects of nasal tufts and nasal respiration on thermoregulation and evaporative water loss in the common crow. The Condor 78: 564-567.
Yarbrough C.G. 1971. The influence of distribution and ecology on the thermoregulation of small birds. Comparative Biochemistry and Physiology 39A: 235-266.
