# Supplementary material for: ﻿Metabolic rate, sleep duration, and body temperature in evolution of mammals and birds: the influence of geological time of principal groups divergence
Source: Zookeys. 2023 Feb 14;1148:1–27. doi: 10.3897/zookeys.1148.93458 (PMC10208811; doi:10.3897/zookeys.1148.93458)
Supplement: Supplementary material 3 — Sleep duration for endothermic species (by review Cambell & Tobler, 1984) and others [file zookeys-1148-001_article-93458__-s003.pdf]

Supplementary information Table 3

Sleep duration for endothermic species (by review Cambell & Tobler, 1984) and others

Species	Body mass, g	Sleep duration, hour/day	
**Mammalia**			
**Monotremata**			
Tachyglossus aculeatus	2725.0	8.60	
Tachyglossus aculeatus	2600.0	12.30	Siegel 1997
Ornithorhynchus anatinus	2000.0	14.30	Siegel et al. 1999
Mean		11.73	
Standard Error		2.89	
**Marsupialia**			
Didelphis marsupialis	1530.0	19.40	
Lutreolina erassicaudata	750.0	19.40	
Trichosurus vulpecula	1993.5	13.70	
Megaleia rufa	30000.0	15.50	
Potorous apicalis	1250.0	11.60	
Mean		15.92	
Standard Error		3.46	
**Eutheria**			
Eutheria			
Erinaceus europaeus	750.0	10.10	
Paraechinus hypomelas	550.0	10.30	
Scalopus aquaticus	74.6	8.40	
Condylura cristata	55.0	10.30	
Talpa europaea	100.0	10.50	
Tupaia glis	123.0	8.90	
Cryptotis parva	6300.0	9.10	
Blarina brevicauda	20.9	14.90	
Suncus murinus	39.7	12.80	
Sorex araneus	8.4	7.80	
Sorex minutus	3.0	8.50	
Neomys fodiens	16.0	13.60	
Centetes ecaudatus	650.0	15.60	
Myotis lucifugus	6.5	19.90	
Eptesicus fuscus	13.3	19.50	
Nycticebus coucang	1128.6	11.00	
Galago senegalensis	171.5	7.80	
Lemur macaco fulvus	850.0	9.40	
Phaner furcifer	400.0	11.50	
Aotus trivirgatus	914.5	17.00	
Saimiri sciureus	836.7	9.90	
Cercopithecus aethiops	6000.0	10.30	
Erythrocebus patas	3000.0	10.80	

Macaca mulatta	8000.0	11.80
Macaca nemestrina	9600.0	7.80
Macaca radiata	5500.0	9.30
Papio papio	19500.0	8.80
Papio anubis	19500.0	9.80
Papio hamadryas	12671.0	9.80
Papio cynocephalus	17500.0	6.20
Pan troglodytes	50000.0	9.70
Bradypus tridactylus	3875.0	15.80
Choloepus hoffmanni	6000.0	16.40
Priodontes giganteus	20500.0	18.10
Dasypus novemcinctus	3414.0	17.40
Oryctolagus cuniculus	2168.0	8.80
Aplodontia rufa	707.0	14.40
Citellus tridecemlineatus	125.0	13.90
Citellus undulatus	750.0	16.60
Citellus lateralis	260.0	14.50
Tamias striatus	89.6	16.30
Eutamias dorsalis	70.0	14.90
Perognathus longimembris	16.5	6.60
Peromyscus erimicus	21.0	10.90
P. gossypinus	31.0	7.80
P. leucopus	22.3	7.80
P. maniculatus	20.5	12.90
P. polionotus	12.0	13.10
Onychomys leucogaster	32.5	14.50
Microtus canicaudus	20.0	10.80
M. montanus	20.0	11.40
Microtus ochrogaster	48.2	15.90
M. pennsylvanicus	42.0	13.70
Neofiber affeni	275.0	14.60
Mesocricetus auratus	108.2	14.40
Sigmodon hispidus	162.0	11.30
Merionis unguiculatus	64.8	15.30
Rattus norvegicus	207.0	13.20
Rattus norvegicus	218.0	13.70
Rattus norvegicus	221.0	13.20
Rhabdomys prinulus	31.0	8.50
Mus musculus	18.0	12.80
Cavia porcellus	640.0	12.60
Chinchilla laniger	437.0	12.50
Octodon degu	200.0	6.90
Platanista indi	84000.0	7.00
Phocoenoides dalli	175000.0	1.50
Tursiops truncatus	400000.0	10.40
Lagenorhynchus obliquidens	103000.0	7.00
Delphinapterus leucas	1400000.0	5.20
Orcinus orca	7200000.0	1.30

Globicephala scammoni	2900000.0	5.30	
Canis domesticus	10000.0	12.90	
Canis domesticus	27000.0	8.40	
Canis lupus	50000.0	13.00	
Alopex lagopus	3600.0	12.50	
Vulpes vulpes	4580.0	9.80	
Felis domestica	4000.0	13.20	
Panthera onca	50400.0	10.00	
Halichoerus grypus	180000.0	14.00	
Gallerinus ursinus	55000.0	3.60	
Elephus maximus	3672000.0	3.30	
Loxodonto africano	6000000.0	8.00	
Procavia johnstoni	2458.0	4.90	
Heterohyrax brucei	1604.4	5.70	
Dendrohyrax validus	3000.0	4.90	
Equus caballus	600000.0	2.90	
Equus asinus	177500.0	3.10	
Tapirus terrestris	200000.0	4.40	
Ovis aries	90000.0	3.80	
Bos taurus	347000.0	4.00	
Sus domesticus	135000.0	7.80	
Capra aegagrus hircus	45000.0	5.40	
Mean		10.49	
Standard Error		4.17	
**Aves**			
**Paleognathae**			
Eudromia elegans	600.0	13.00	Tisdale et al. 2017
Strutio camelus	82600.0	8.00	Lesku et al. 2011
Dromiceus novae-hollandiae	39000.0	8.70	Moscow Zoo (pers.com)
Mean		9.90	
Standard Error		2.71	
**Neognathae, Non-Passeriformes**			
Gallus domesticus	2200.0	11.70	
Anser anser	3250.0	6.20	
Anas platyrhynchos domesticus	1500.0	10.80	
Buteo jatnaicensis arborealis	1100.0	4.50	
Herpetotheres cachinnans chapmanni	550.0	4.50	
Aptenodytes forsteri	24000.0	10.70	
Eudyptula minor	1200.0	8.30	
Columba livia	300.0	10.60	
Streptopelia capicola	150.0	10.20	
Aratinga canicularis	80.0	9.40	
Speotyto cunicularis hypugaea	140.0	14.30	
Strix aluco	520.0	16.00	
Nyctea scandiaca	2100.0	7.90	
Anas acuta	720.0	8.10	
Anas crecca carolinensis	250.0	8.00	

Anas crecca crecca	260.0	8.20
Anas rubripes	1500.0	6.90
Apus apus	50.0	10.60
Aythia ferina	1000.0	12.80
Aythia fuligula	650.0	13.20
Bucephala clangula	1100.0	6.40
Calidris m. marttima	80.0	3.50
Calidris pusilla	28.0	2.70
Calypte anna	4.8	11.10
Catharus minimus	30.0	3.30
Cygnus c. buccinator	12500.0	5.70
Cygnus c. cygnus	9000.0	12.00
Larus argentatus	1200.0	5.00
Meleagris gallapavo	3100.0	3.90
Mergus albellus	700.0	13.40
Phalaropus fulicarius	55.0	3.10
Plectrophenax nivalis	36.0	4.60
Rissa tridactyla	420.0	6.30
Somateria mollissinta	2400.0	4.30
Spheniscus mendiculus	2500.0	12.80
Sterna paradisaea	400.0	3.50
Strix varia	750.0	3.90
Mean		8.06
Standard Error		3.74
**Neognathae, Passeriformes**		
Sturnus vulgaris	70.0	4.80
Fringilla coelebs	21.0	7.00
Acanthis hornemanni	14.0	3.40
Delichon urbica	20.5	3.30
Oenanthe oenanthe	25.0	4.80
Parus major major	17.5	8.00
Parus rufescens	11.0	11.70
Thryomanes bewickii	10.0	11.30
Turdus migratorius	75.0	3.70
Mean		6.44
Standard Error		3.28

References to Table 3 Sleep duration

Campbell S, Tobler I, (1984). Animal sleep: A review of sleep duration across phylogeny. Neuroscience and Biobehavioral Reviews 8: 269–300.

Siegel J. M. 1997. Sleep in Monotremes; implications for the evolution of rem sleep Pages 113-128 In: Sieep and Sleep Disorders: Takeda Science Foundation From Molecule to Behavior.

Siegel, J.M.; P.R. Manger; R. Nienhuis; H.M. Fahringer; T. Shalita; J.D. Pettigrew (1999). "Sleep in the platypus". Neuroscience. Elsevier. 91 (1): 391–400. doi:10.1016/S0306-4522(98)00588-0. PMID 10336087.

Tisdale R. K., Vyssotski A.L., Lesku J.A., Rattenborg N.C. 2017. Sleep-Related Electrophysiology and Behavior of Tinamous (Eudromia elegans): Tinamous Do Not Sleep Like Ostriches. Brain Behav Evol: 1-13, DOI: 10.1159/000475590
